# Data regarding the computational fluid dynamics simulations of an airfoil with plasma actuator in unsteady flow

**DOI:** 10.1016/j.dib.2020.105286

**Published:** 2020-02-13

**Authors:** Maria Grazia De Giorgi, Valentina Motta, Antonio Suma

**Affiliations:** University of Salento, Department of Engineering for Innovation, GE Renewable Energy, Advanced Technology & Sciences, Germany

**Keywords:** Load alleviation, Plasma actuation, Active flow control, Oscillating airfoils, Actuation law, Stability control

## Abstract

The data regard the analysis reported in the research article “Influence of actuation parameters of multi-DBD plasma actuators on the static and dynamic behavior of an airfoil in unsteady flow” [1]. The data are related to the study focused on the evaluation of the effects of an active flow control system on the performance of an airfoil in an unsteady flow, with particular focus on the influence of actuation parameters on the global performances.

**Table:** Specifications Table

Subject area	Aerospace Engineering, Mechanical Engineering
More specific subject area	Flow control techniques, Aeroelasticity, Active Flow Control
Type of data	PNG images (graphs), text file (Excels data)
How data was acquired	Ansys Fluent Release 19.2
Data format	Raw, Analyzed
Experimental factors	Numerical analysis involves a NACA23012 airfoil in an unsteady free-stream flow. Flow conditions, as performed in Motta et al. [2], are: velocity of 31.16 m/s, pressure equal to 1 atm and temperature equal to 288.15 K, which brings to a Reynolds number Re = 600′000. Unsteadiness are reproduced through a time-variable farfield velocity. Two groups of Dielectric Barrier Discharge Plasma Actuators (DBD-PAs) are applied at the airfoil trailing edge. One of the groups has the effect to increase lift, while the other one decreases its. The influence of the actuation law has been analyzed through changing the force phase θ that regulates turning ON and turning OFF timing of the actuators.$\begin{array}{c} {\text{F}}_{0,\text{UP}}=\{\begin{array}{c} 0,\;\;\text{if}\left[\sin\left(\text{ωt}-\theta \right)<0\right]\\ {\text{F}}_{0},\;\text{if}\left[\sin\left(\text{ωt}-\theta \right)\geq 0\right] \end{array}\\ {\text{F}}_{0,\text{DOWN}}=\{\begin{array}{c} {\text{F}}_{0},\;\text{if}\left[\sin\left(\text{ωt}-\theta \right)<0\right]\\ 0,\;\text{if}\left[\sin\left(\text{ωt}-\theta \right)\geq 0\right] \end{array} \end{array}$ (1)
Experimental features	Numerical simulation were performed by using the software Ansys Fluent Release 19.2. Due to the low Mach number (M = 0.09), and to the slight thermal effect of DBDs, the Navier-Stokes equations have been resolved with the hypothesis of incompressible flow. Unsteady simulations have been carried out by using a fixed time step of 1/200 of the pitching period of the highest simulated frequency condition, which corresponds to a time step of 3.14 × 10^−4^ s. Turbulence has been modelled through the Spalart-Allmaras turbulence model.
Data source location	Lecce, Italy
Data accessibility	Data of current article
Related research article	“Influence of actuation parameters of multi-DBD plasma actuators on the static and dynamic behavior of an airfoil in unsteady flow” [1].

**Table:** 

**Value of the Data** • Simulations allow to evaluate the effect of the application of an active flow control technique based on Dielectric Barrier Discharge Plasma Actuator to control loads on an airfoil in an unsteady flow; • data can be used to evaluate the best actuation switching frequency for different unsteadiness reduced frequencies; • the numerical data about the temporal evolution of lift and moment coefficients could be used to verify modeling predictions of unsteady flow over an airfoil; • the role of actuation switching law for flow control in pitching airfoil has been highlighted; • the data can be used for comparing with the results of the application of other active flow control techniques for load alleviation of an airfoil under unsteady flow; • the data can serve as a benchmark for future research on active flow control on unsteady flow over an airfoil.

## 1 Data

Temporal evolution of lift (C_L_) and moment coefficients (C_M_) are provided for three different unsteadiness reduced frequencies and eight different actuation switching laws phases. Moreover, summary tables for significant parameters of Fourier Series approximation of C_L_ and C_M_ have been presented.

The evaluated parameters for the C_L_ involve the phase shift, the mean value and the standard deviation in a cycle of fluctuations. About the C_M_, instead, calculation have included to the parameters already calculated for the C_L_, also the imaginary part of the first coefficient of Fourier series, the hysteresis area, the static and the dynamic pitching coefficients.

For all the reduced frequencies end the actuation law phases, the following data are provided:

1. Tables with temporal evolution of lift, drag and moment coefficient as calculated by Ansys Fluent© software (file “Force_wXXX_a1_YY.xlsx”, in which XXX is the pitching pulse and YY the phase of the applied actuation law);
2. Comparison plots between clean and actuated temporal evolution of both lift and moment coefficients (“Lift Coefficient comparison.png”, “Moment Coefficient comparison.png”);
3. Comparison plots of clean and actuated moment coefficient hysteresis area, with the line on which is calculated the static pitch coefficient.
4. Performance comparison between the different actuation conditions analyzed in synthetic tables (section “summary tables”).

## 2 Experimental design, materials, and methods

An unsteady flow around a NACA23012 airfoil has been analyzed. The flow is directed with an average relative angle with respect to the airfoil of 7.7 deg, and a velocity magnitude of 31.16 m/s. The horizontal and vertical components of the flow free-stream velocity, change during time with a sinusoidal law, bringing to an unsteadiness in the real angle of attack.

The fluid flow is considered as incompressible, due to the Mach <0.3 and the absence of heating source or regions with strongly different pressure. Temperature has been assumed constant in all the domain, with a value of 288.15K. Pressure is set at 1 atm, so the density has been taken equal to 1.1835 kg/m^3^. The airfoil chord is 0.30 m and the Reynolds number is 600′000.

Simulation have been carried out by using the software Ansys Fluent Release 19.2. Turbulence has been modelled by using the Spalart-Allmaras RANS, while the pressure-velocity coupling equations have been solved through a Semi-Implicit Method for Pressure Linked Equations (SIMPLE) scheme. Time step have been fixed as 1/200 of the pitching period of the highest reduced frequency simulated, which corresponds to a time step of 3.14 × 10^−4^ s.

Computational domain consists of a 99,604 quadrangular elements C-Mesh, which reproduces a single NACA23012 airfoil in a free-stream flow. External domain boundaries are located more than 30 chords away from the airfoil.

Load response, evaluated in terms of lift and moment coefficients have been analyzed. Moment coefficient is considered positive when induces a nose-down torsion on the airfoil.

Based on the lift and moment coefficient temporal evolution, the phase lag between loads and instantaneous angle of attack, the mean value and the signal standard deviation have been calculated. Moreover, the moment coefficient imaginary part of the first Fourier series coefficient and hysteresis area have been computed for analysis. At the end, airfoil stability is evaluated through the static and dynamic pitch stability coefficients.

The phase lag, reported in degrees, represents the difference between the pulses at which correspond respectively the maximum peak of the instantaneous angle of attack and the maximum peak of the lift or moment signal.

The signal standard deviation is calculated for both the lift and the moment coefficients with the formula:

(2) $$\sigma =\sqrt{\frac{1}{N-1}\sum_{t=0}^{T}{\left(C_{L}\left(t\right)-C_{L,\;mean}\right)}^{2}}$$

Signals are approximated through a Fourier series with complex-valued coefficients, obtaining a filtered sinusoidal signal of the load coefficients. The imaginary part of the first coefficient of Fourier series$\text{ Im}\left({\text{a}}_{1,C_{M}}\right)$ can suggest if the blade is aerolastically stable or unstable. In particular $\text{Im}\left({\text{a}}_{1,C_{M}}\right)$ <0 represents a stable behavior.

(3) $$\{\begin{array}{c} {\text{C}}_{L}\left(\text{t}\right)={\text{a}}_{0,C_{L}}+\text{Re}\left({\text{a}}_{1,C_{L}}\right)\cdot {\text{e}}^{\text{i}\cdot \frac{2\text{π}\cdot \text{nt}}{\text{T}}}+\text{i}\cdot \text{Im}\left({\text{a}}_{1,C_{L}}\right)\cdot {\text{e}}^{\text{i}\cdot \frac{2\text{π}\cdot \text{nt}}{\text{T}}}\\ {\text{C}}_{M}\left(\text{t}\right)={\text{a}}_{0,C_{M}}+\text{Re}\left({\text{a}}_{1,C_{M}}\right)\cdot {\text{e}}^{\text{i}\cdot \frac{2\text{π}\cdot \text{nt}}{\text{T}}}+\text{i}\cdot \text{Im}\left({\text{a}}_{1,C_{M}}\right)\cdot {\text{e}}^{\text{i}\cdot \frac{2\text{π}\cdot \text{nt}}{\text{T}}} \end{array}$$

The hysteresis area represents the energy exchanged between flow and airfoil. A positive value for hysteresis area means that the profile works on the flow, having a damping effect on profile oscillation and movements.

(4) $$\text{Area }\;{\text{H}}_{C_{M}}=-\int_{{\text{α}}_{\text{max,i}}}^{{\text{α}}_{\text{max,i}+1}}{\text{C}}_{M,\;{\text{Fourier}}_{1}}\cdot d\text{α}$$

The static pitch stability coefficient is defined as the slope of the line that connects the two extremes angle of attack in a C_M_-α plot. The relevance of this coefficient is that shows the tendency of airfoil to move back to equilibrium position after small disturbances.

The dynamic pitch stability coefficient, instead, evaluates the damping behavior of the airfoil. It is obtained as half of the difference of the C_M_ signal during the pitch up and pitch down phases, divided by the reduced frequency multiplied for the pitching amplitude.

(5) $$\text{Dynamic}\;\text{Pitch}\;\text{Stability}\;\text{coeff}.\;=\frac{\left({\text{C}}_{\text{M},\;\text{pitch}\;\text{up}}-{\text{C}}_{\text{M},\;\text{pitch}\;\text{down}}\right)}{2\cdot {\text{k}}_{\text{q}}}$$

## 3 Summary tables

The value of the analyzed parameters for all the reduced frequencies and force phases tested (27 cases) are summarized by the following tables (see Table 1, Table 2, Table 3).

**Table 1:**
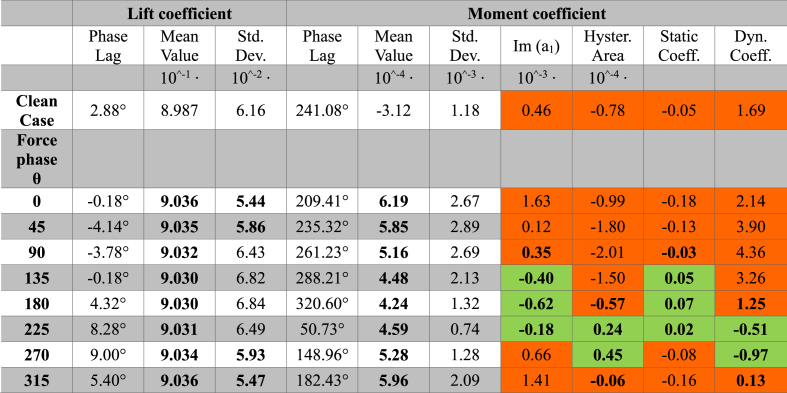
Aerodynamic performance parameters for an external flow with a pulse of 20 rad/s for clean case (baseline case without actuation) and the cases with actuation and different force phase values. Bold characters indicate a parameter of actuated case better than the clean case. For stability coefficients, a green is used when the coefficient suggests a stable behavior, while orange is used for unstable behavior.

**Table 2:**
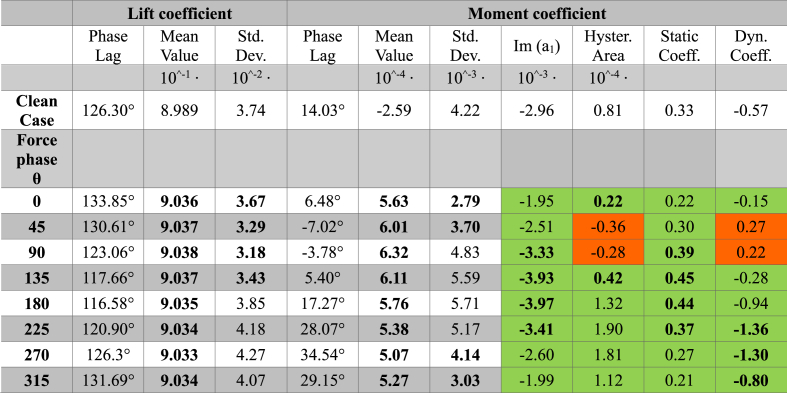
Aerodynamic performance parameters for an external flow with a pulse of 60 rad/s for clean case (baseline case without actuation) and the cases with actuation and different force phase values. Bold characters indicate a parameter of actuated case better than the clean case. For stability coefficients, a green is used when the coefficient suggests a stable behavior, while orange is used for unstable behavior.

**Table 3:**
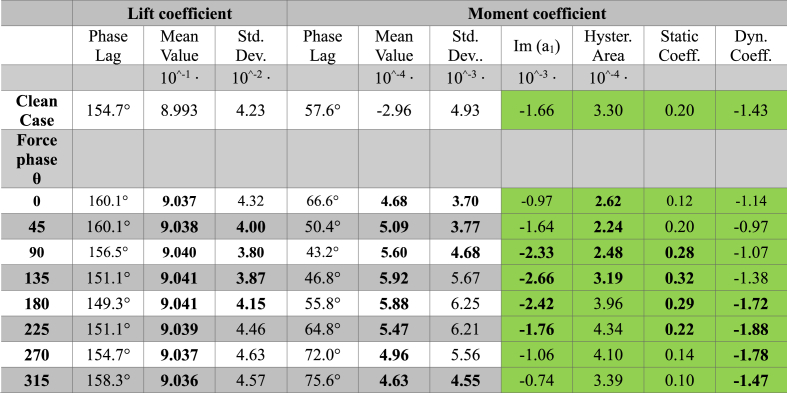
Aerodynamic performance parameters for an external flow with a pulse of 100 rad/s for clean case (baseline case without actuation) and the cases with actuation and different force phase values. Bold characters indicate a parameter of actuated case better than the clean case. For stability coefficients, a green is used when the coefficient suggests a stable behavior, while orange is used for unstable behavior.
