# Supplementary figures and images for: Data regarding the computational fluid dynamics simulations of an airfoil with plasma actuator in unsteady flow
Source: Data Brief. 2020 Feb 13;29:105286. doi: 10.1016/j.dib.2020.105286 (PMC7044653; doi:10.1016/j.dib.2020.105286)

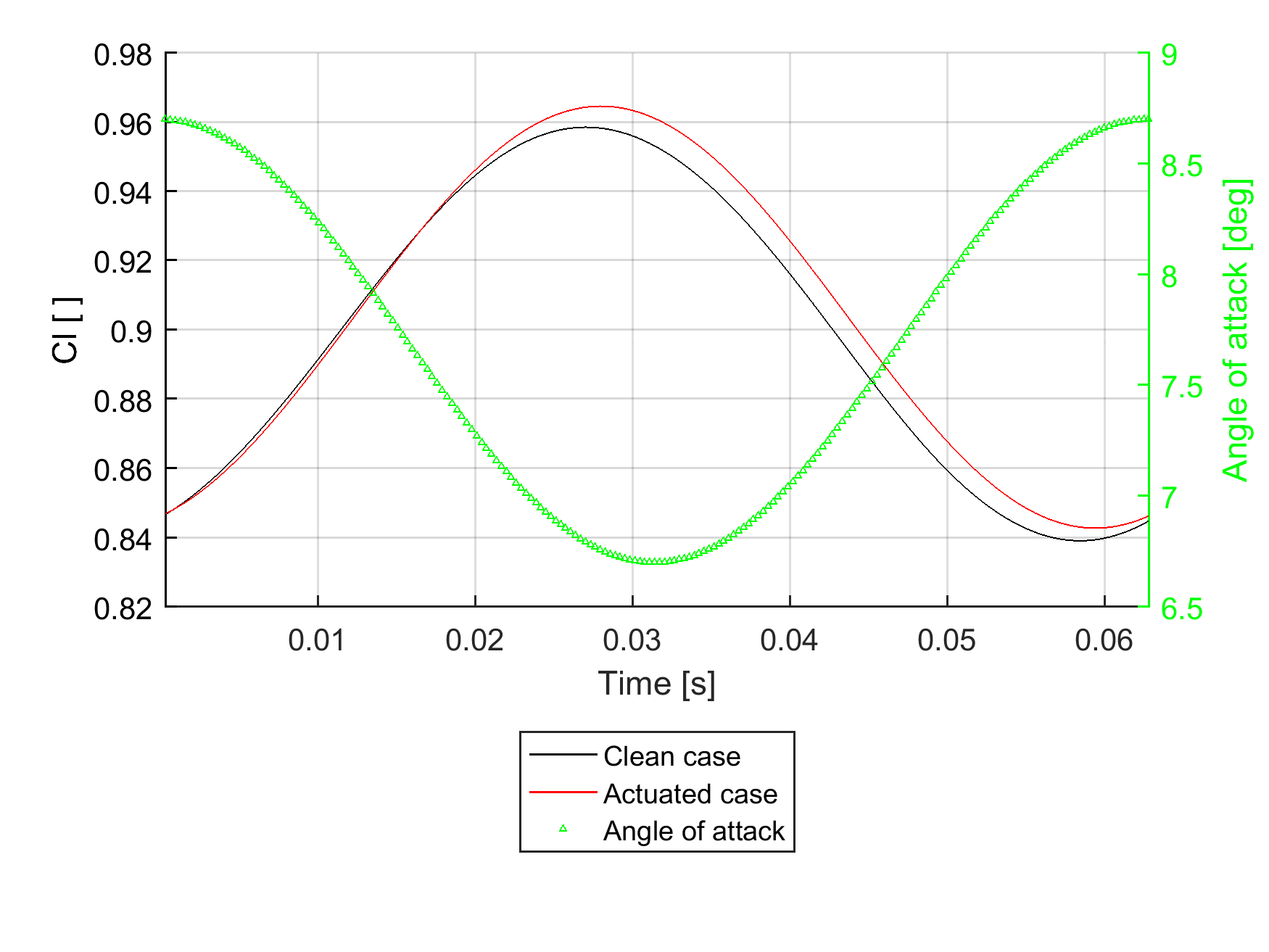

Supplement: Multimedia component 1 [file mmc1.zip › Allegati/w100_a1/Force_w100_a1_0/Lift Coefficient comparison.png]

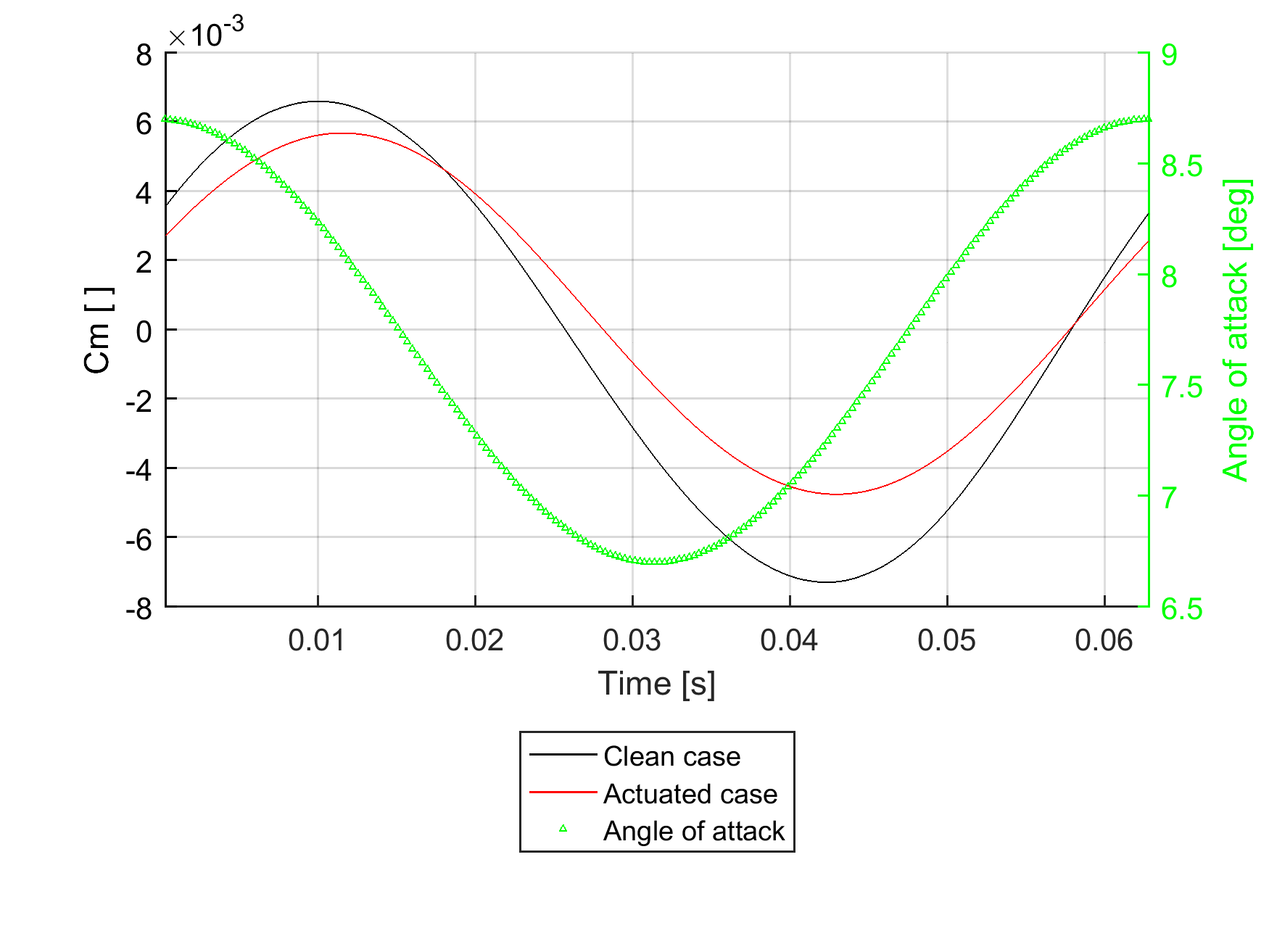

Supplement: Multimedia component 1 [file mmc1.zip › Allegati/w100_a1/Force_w100_a1_0/Moment Coefficient comparison.png]

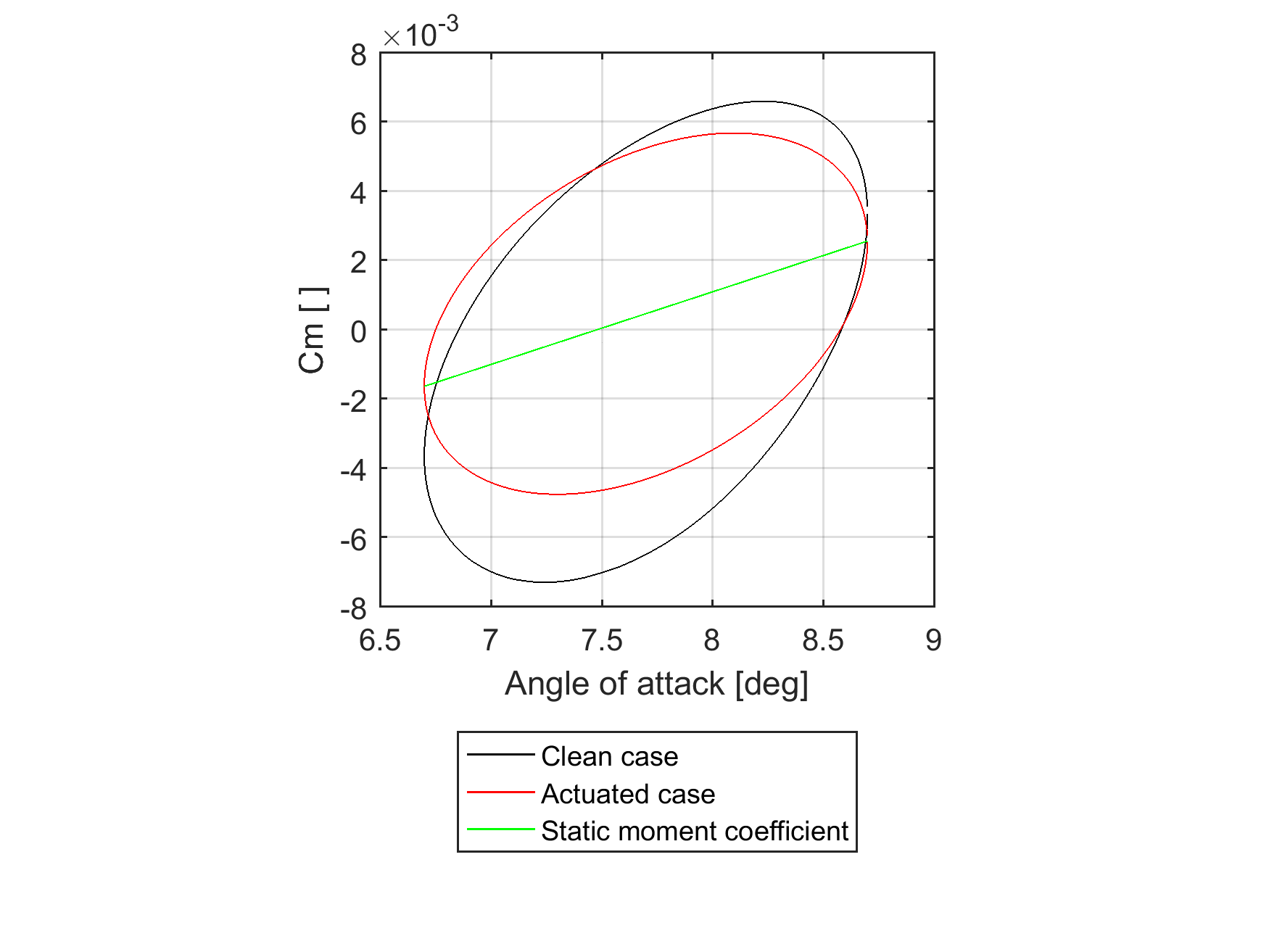

Supplement: Multimedia component 1 [file mmc1.zip › Allegati/w100_a1/Force_w100_a1_0/Moment Coefficient Hysteresis curve.png]

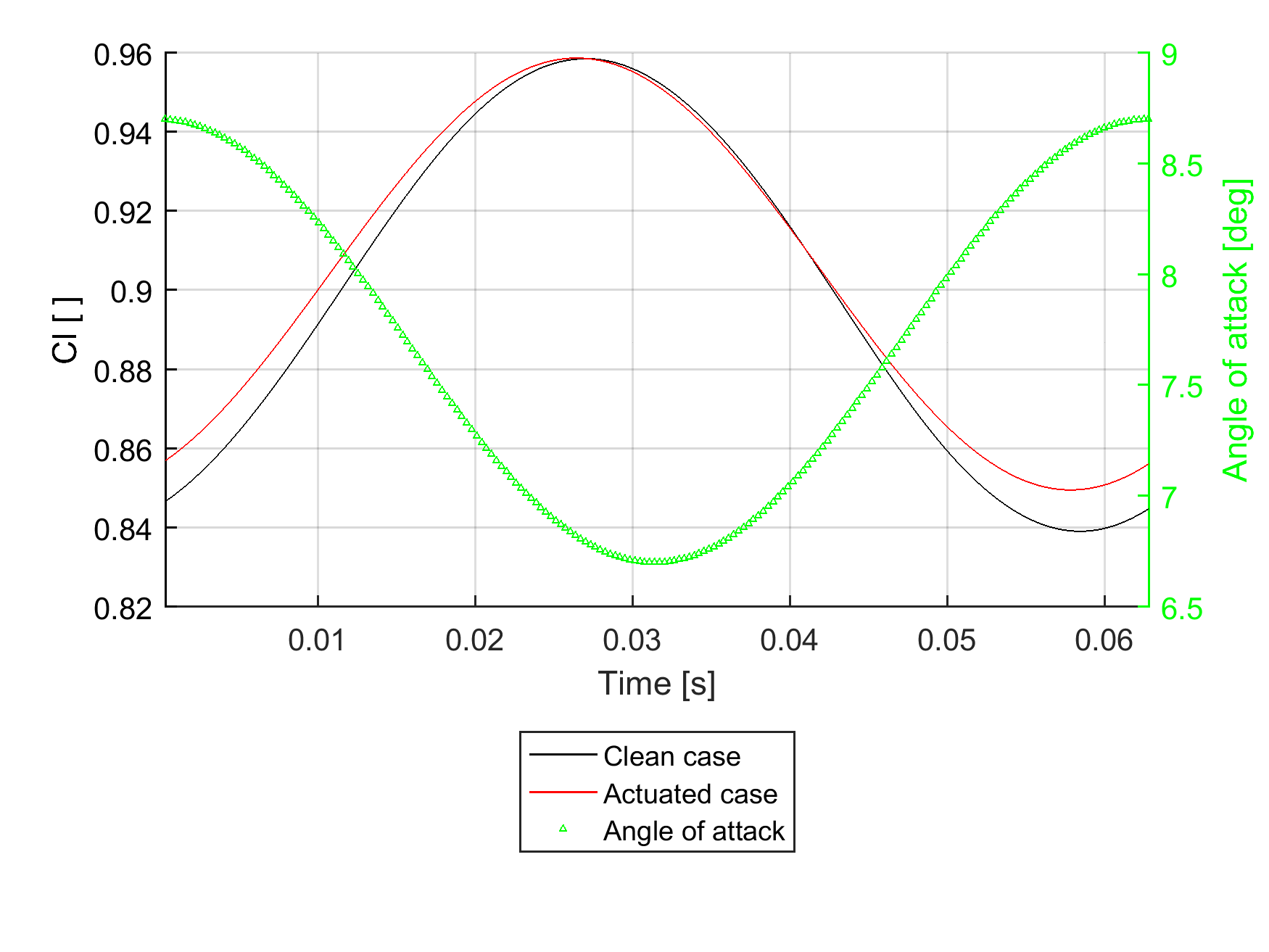

Supplement: Multimedia component 1 [file mmc1.zip › Allegati/w100_a1/Force_w100_a1_135/Lift Coefficient comparison.png]

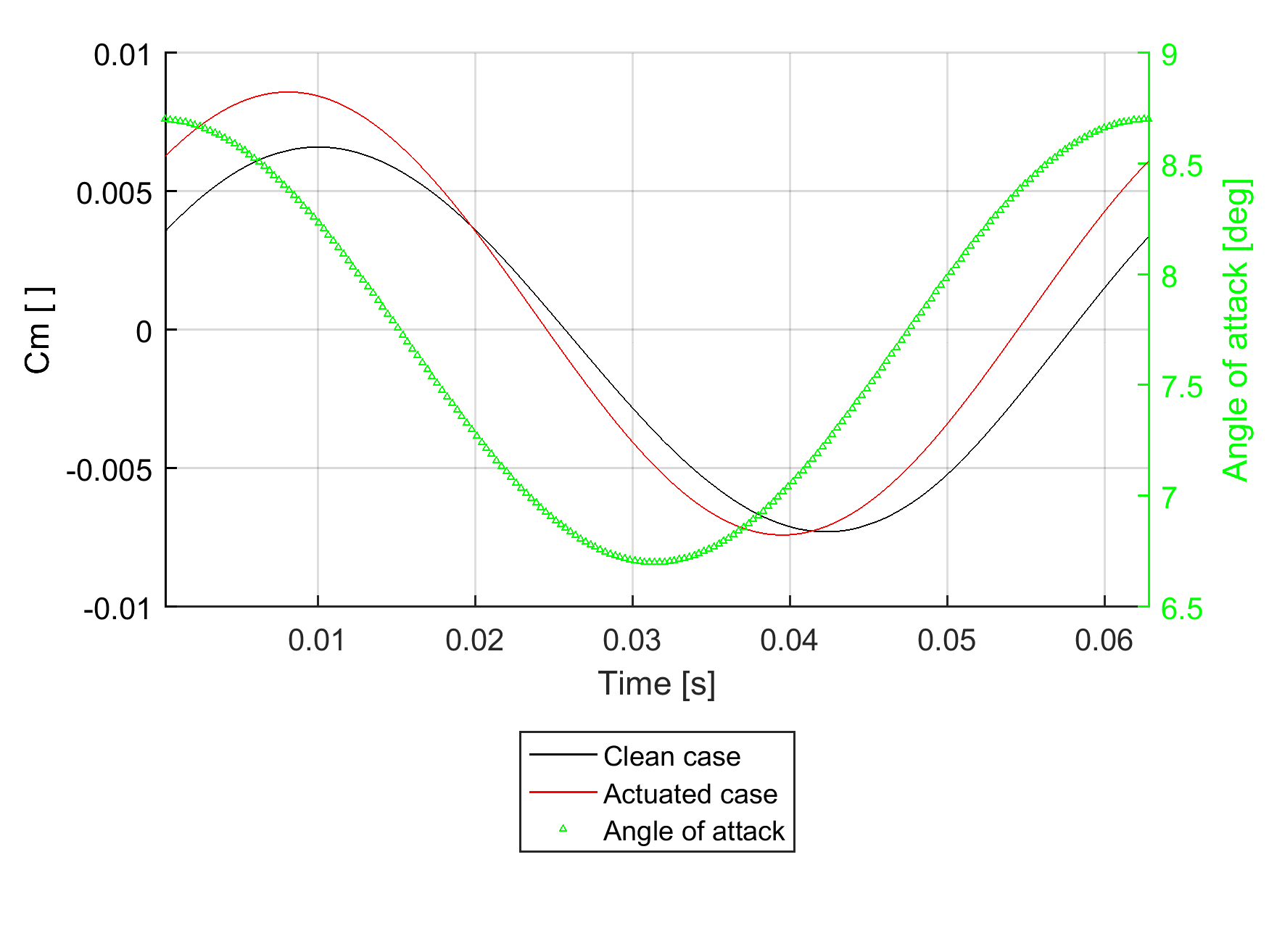

Supplement: Multimedia component 1 [file mmc1.zip › Allegati/w100_a1/Force_w100_a1_135/Moment Coefficient comparison.png]

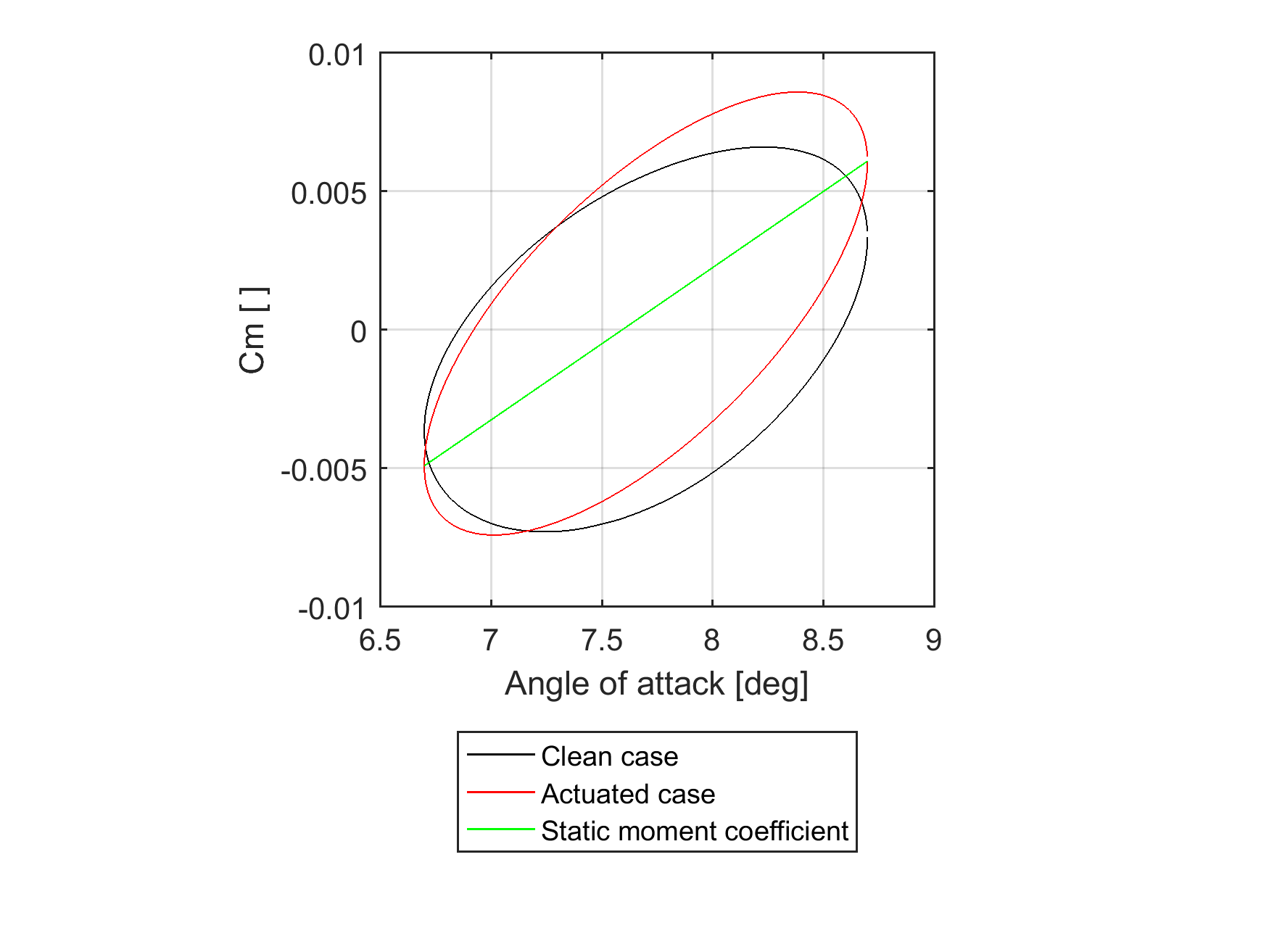

Supplement: Multimedia component 1 [file mmc1.zip › Allegati/w100_a1/Force_w100_a1_135/Moment Coefficient Hysteresis curve.png]

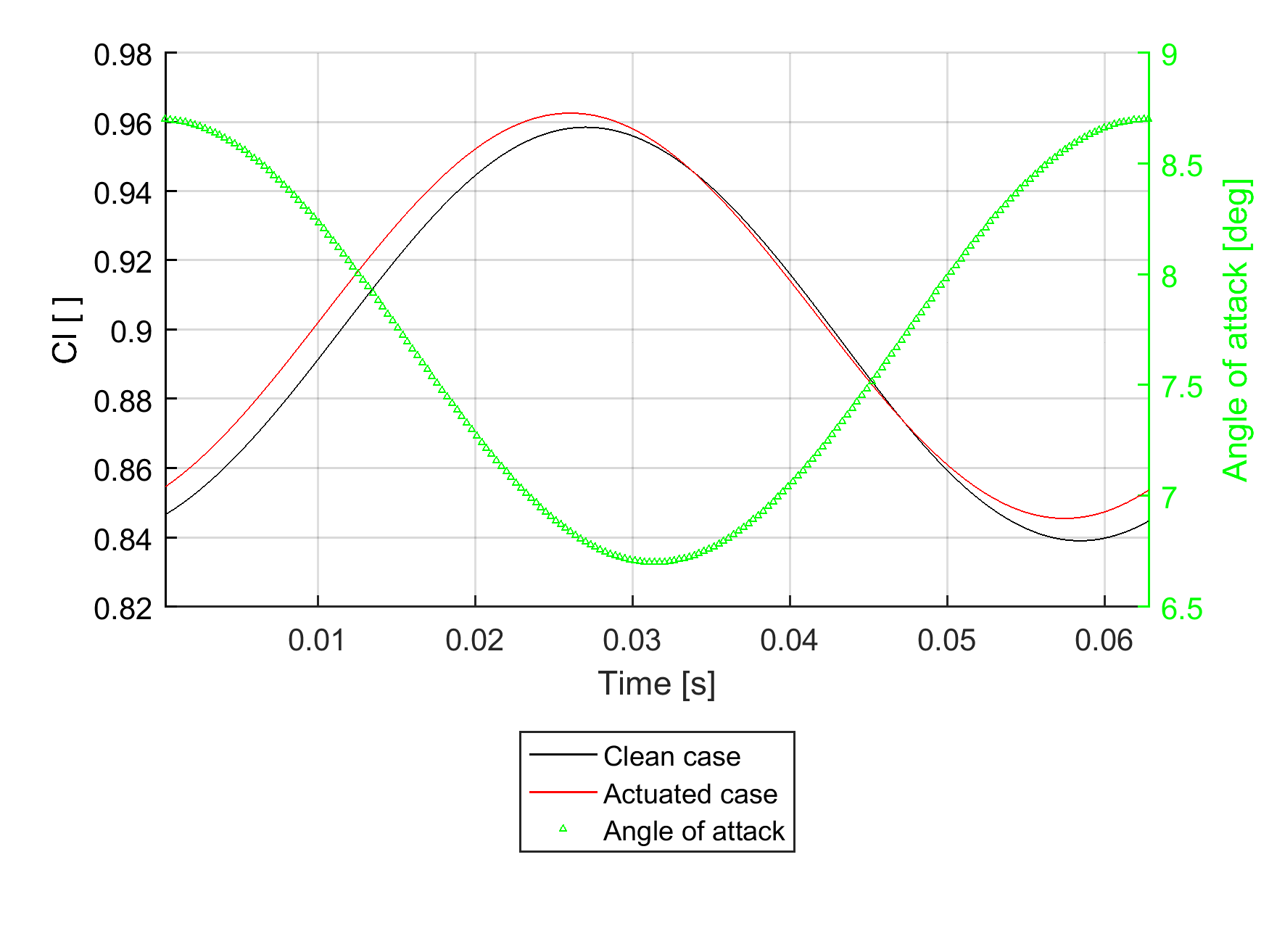

Supplement: Multimedia component 1 [file mmc1.zip › Allegati/w100_a1/Force_w100_a1_180/Lift Coefficient comparison.png]

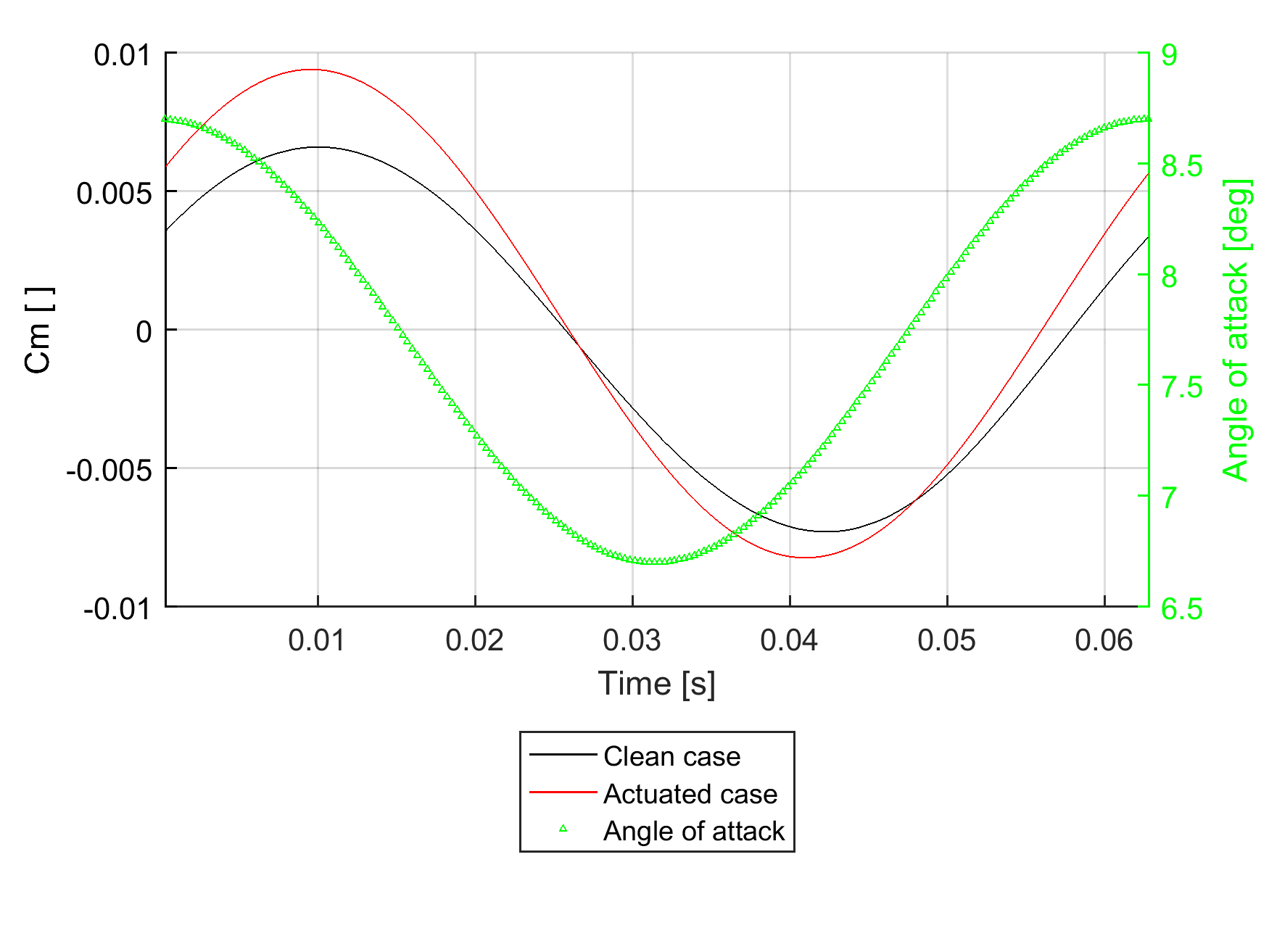

Supplement: Multimedia component 1 [file mmc1.zip › Allegati/w100_a1/Force_w100_a1_180/Moment Coefficient comparison.png]

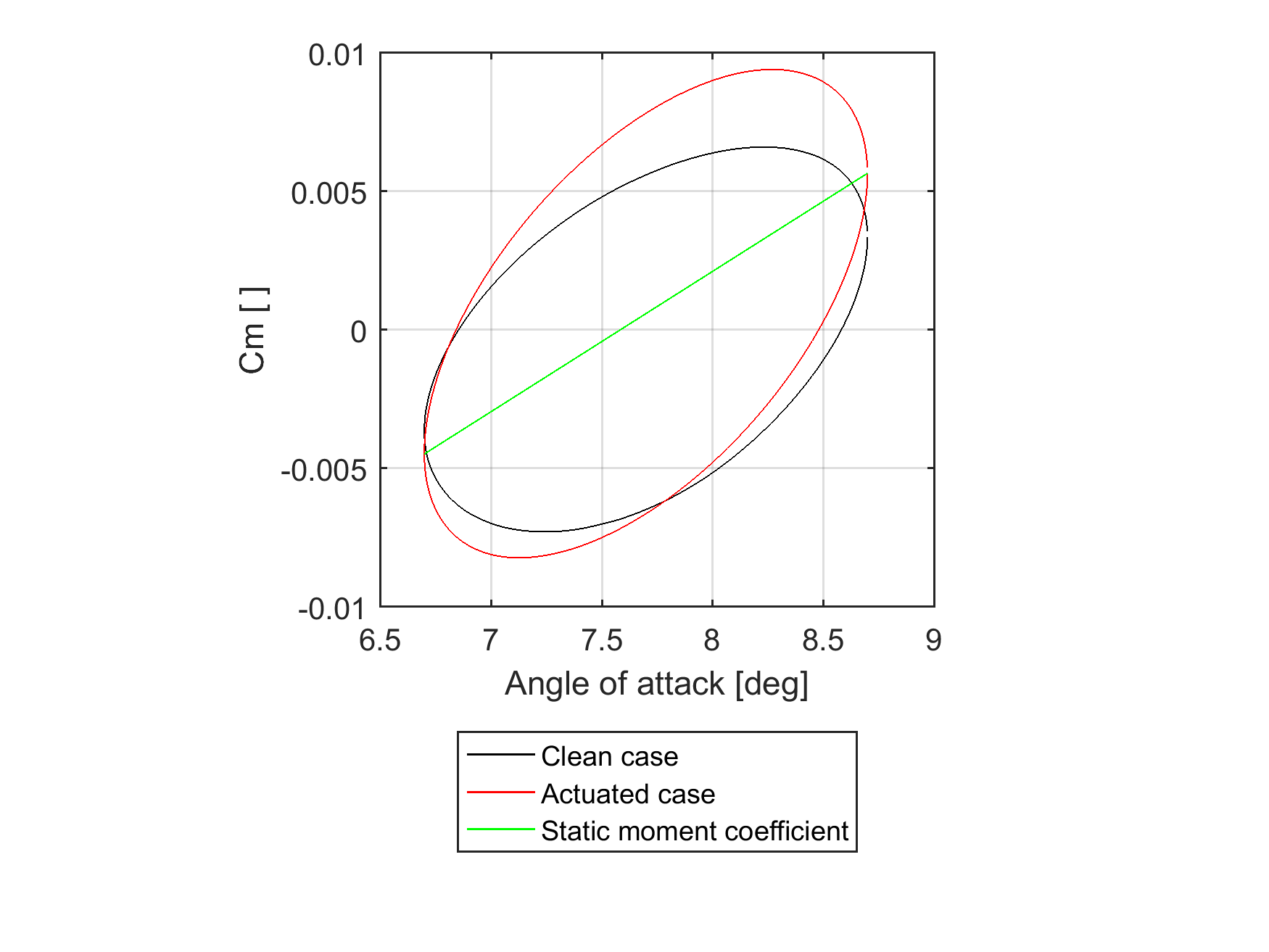

Supplement: Multimedia component 1 [file mmc1.zip › Allegati/w100_a1/Force_w100_a1_180/Moment Coefficient Hysteresis curve.png]

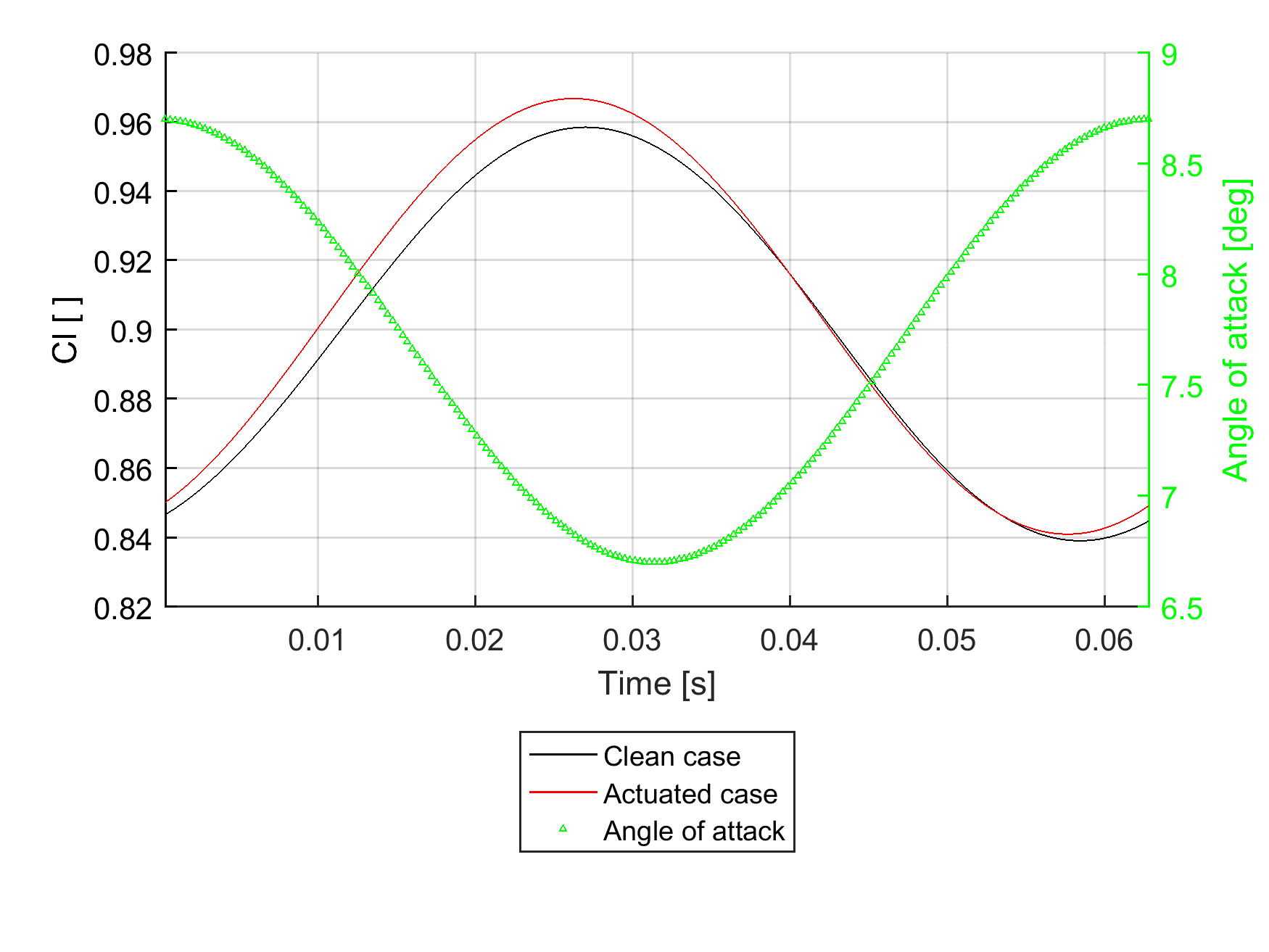

Supplement: Multimedia component 1 [file mmc1.zip › Allegati/w100_a1/Force_w100_a1_225/Lift Coefficient comparison.png]

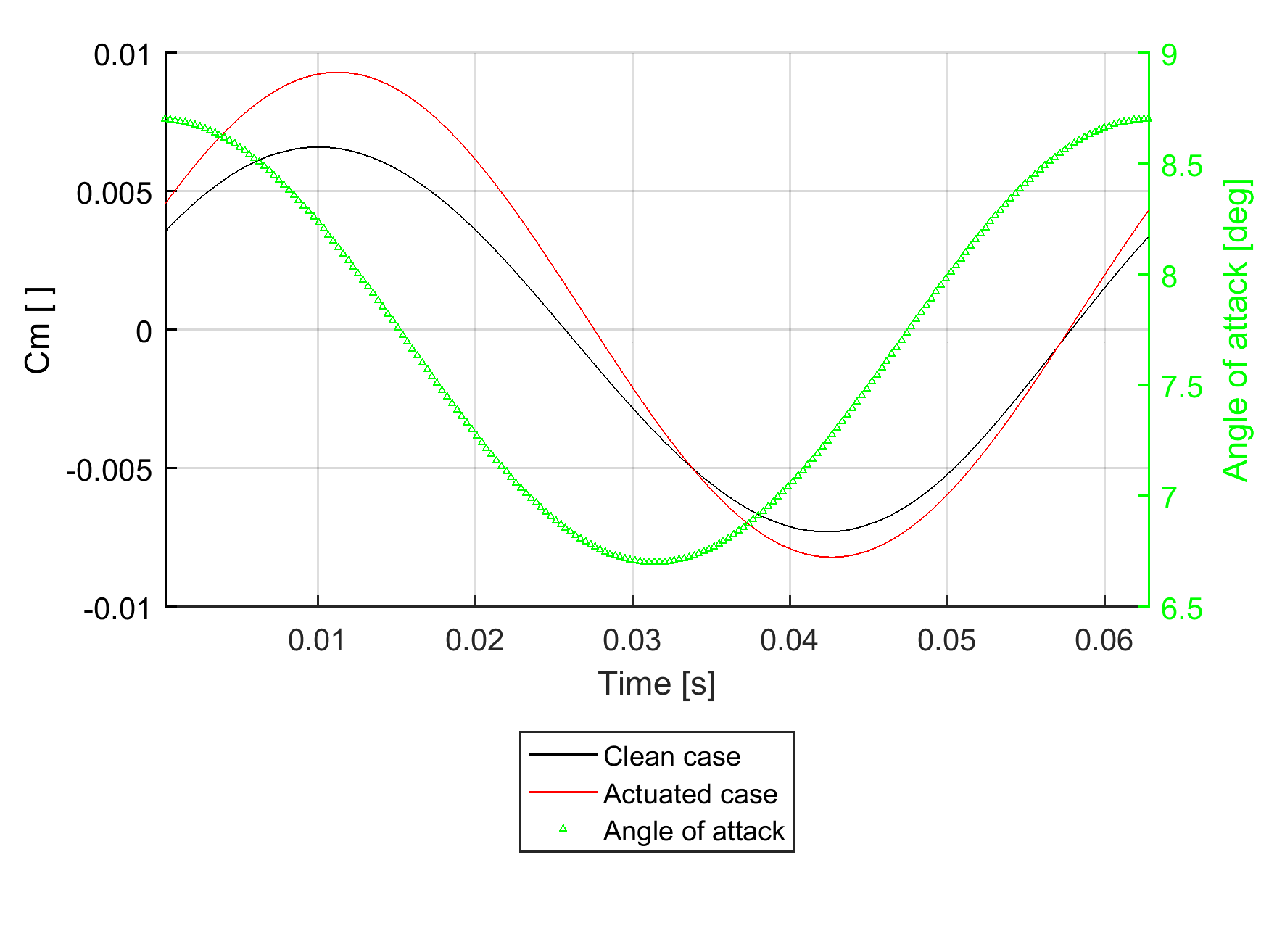

Supplement: Multimedia component 1 [file mmc1.zip › Allegati/w100_a1/Force_w100_a1_225/Moment Coefficient comparison.png]

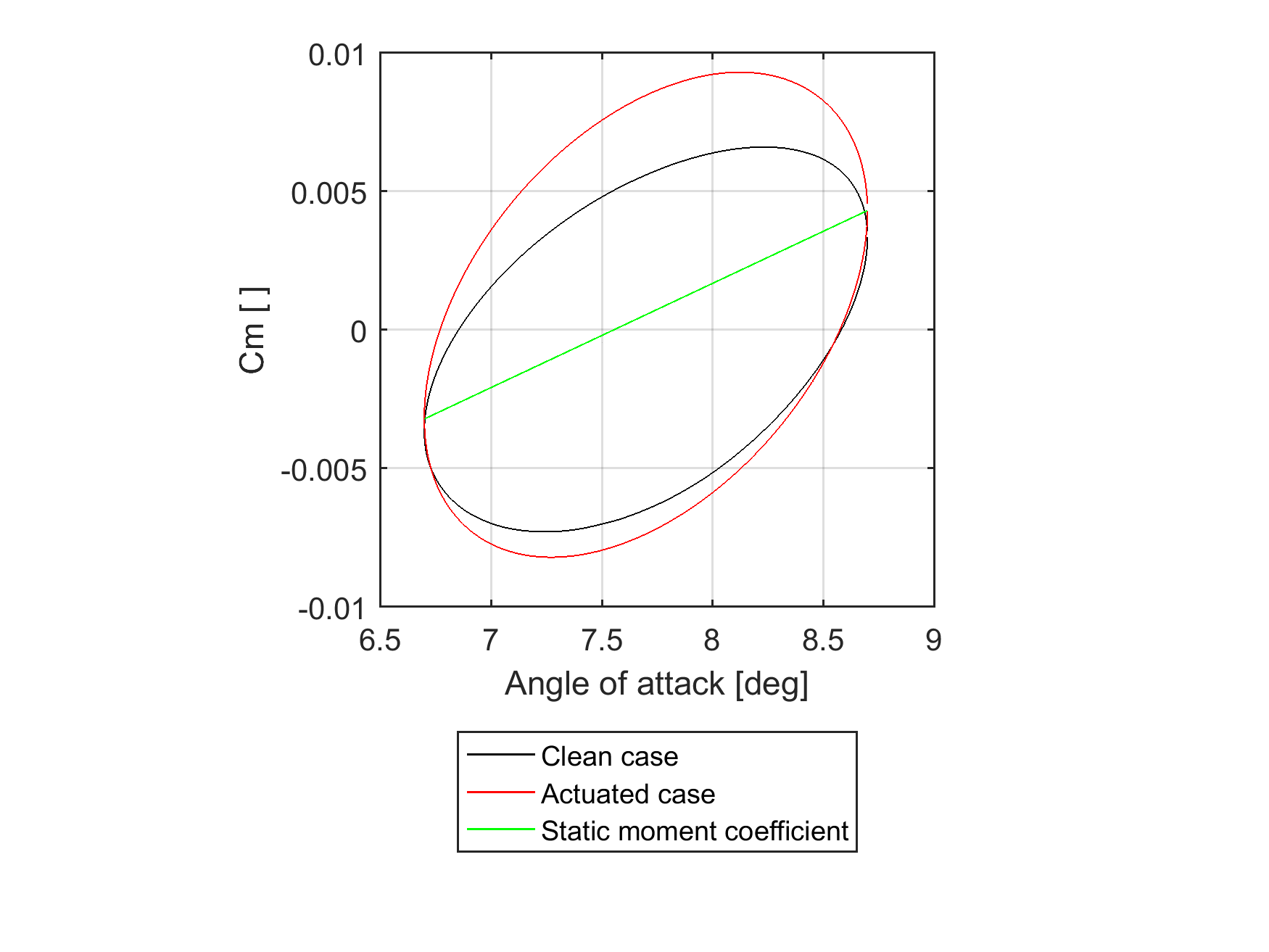

Supplement: Multimedia component 1 [file mmc1.zip › Allegati/w100_a1/Force_w100_a1_225/Moment Coefficient Hysteresis curve.png]

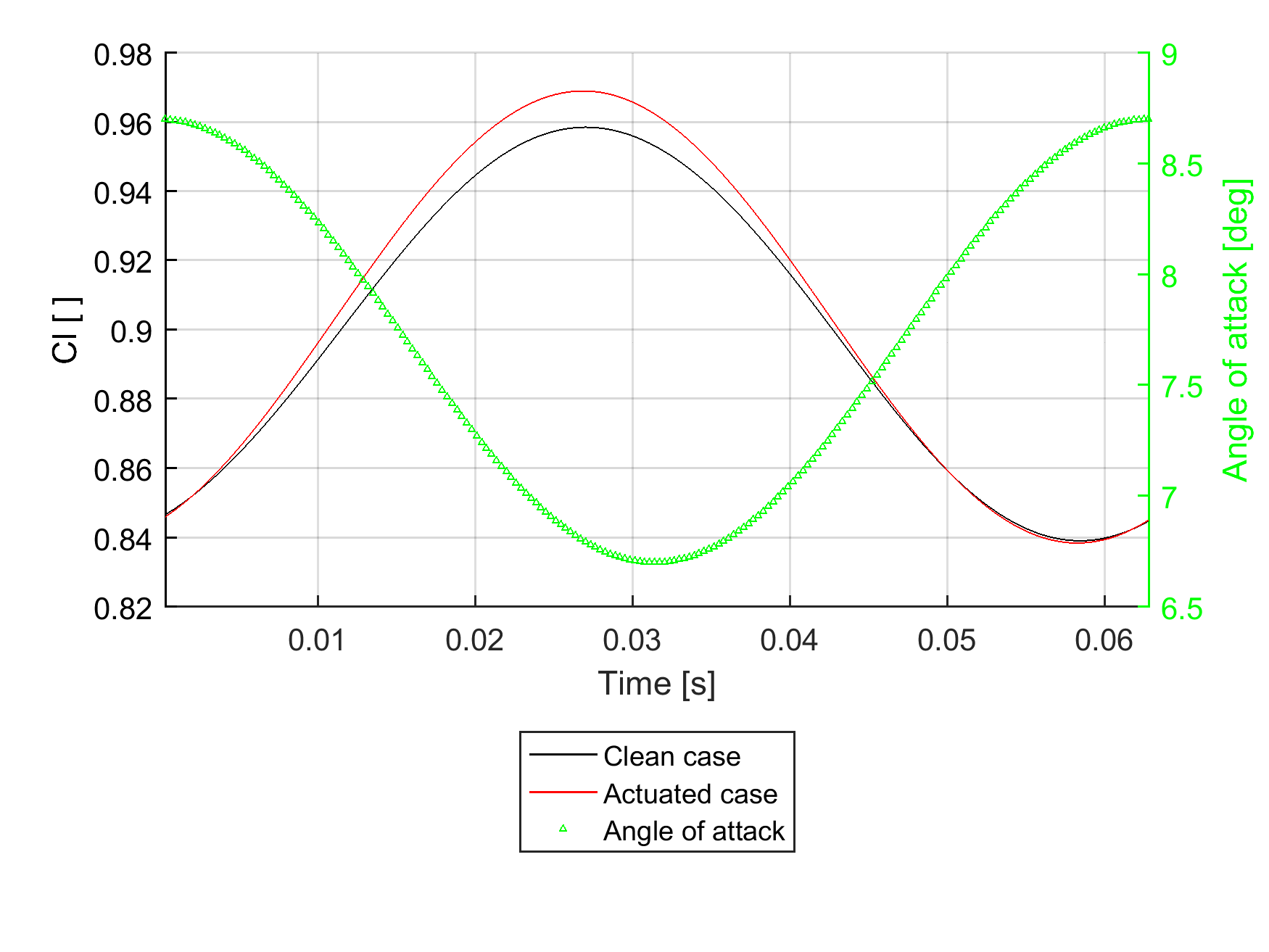

Supplement: Multimedia component 1 [file mmc1.zip › Allegati/w100_a1/Force_w100_a1_270/Lift Coefficient comparison.png]

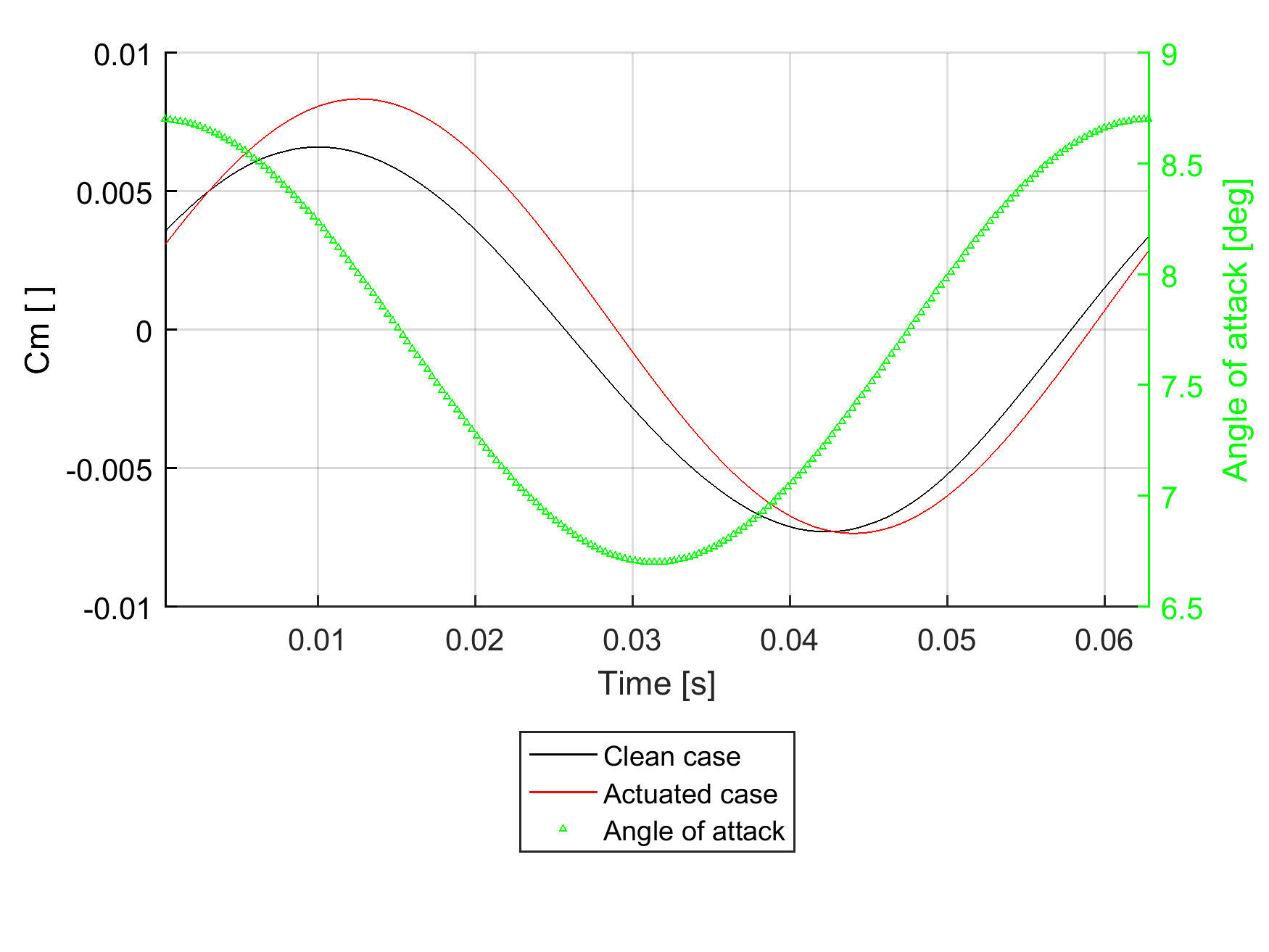

Supplement: Multimedia component 1 [file mmc1.zip › Allegati/w100_a1/Force_w100_a1_270/Moment Coefficient comparison.png]

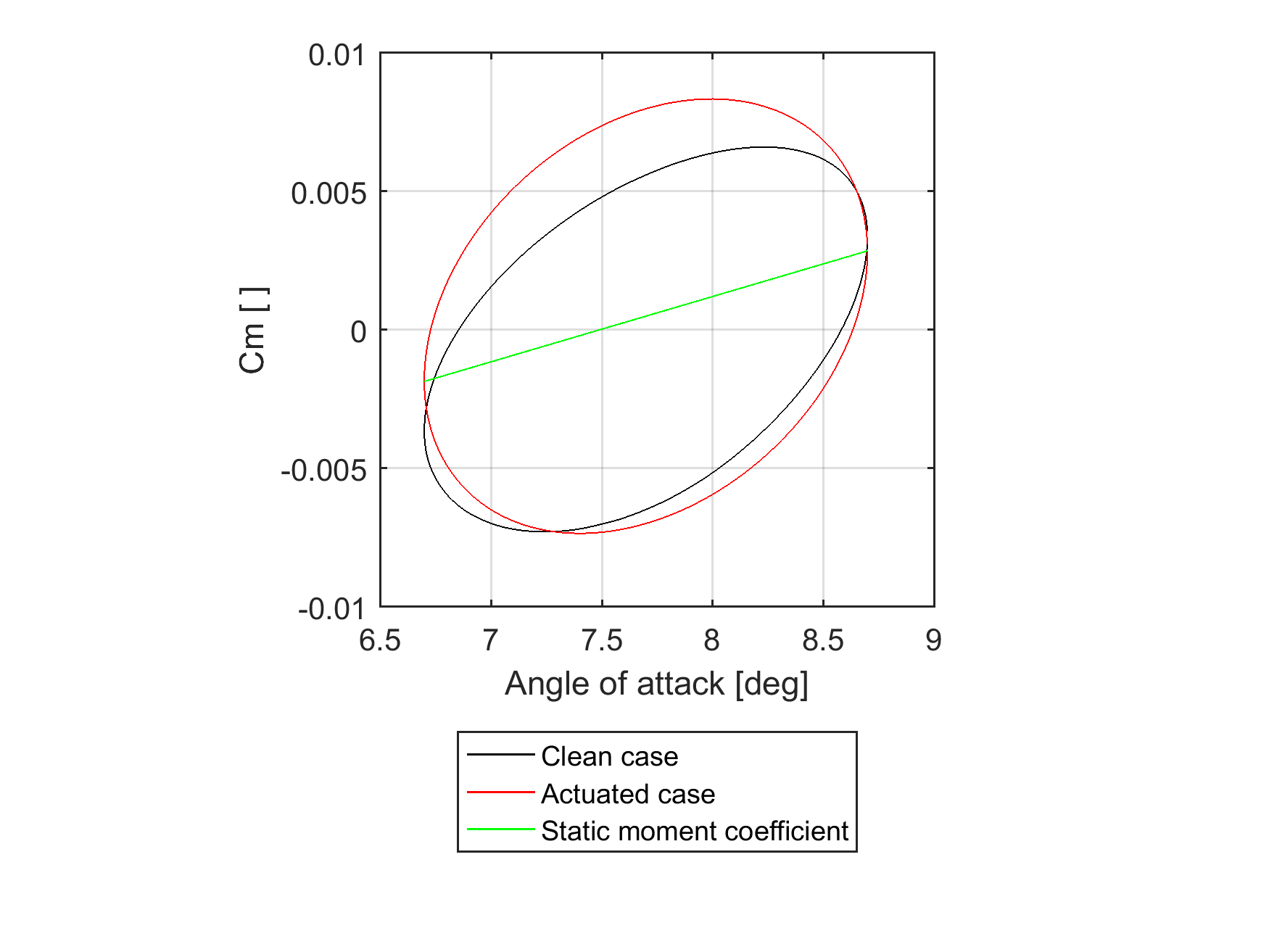

Supplement: Multimedia component 1 [file mmc1.zip › Allegati/w100_a1/Force_w100_a1_270/Moment Coefficient Hysteresis curve.png]

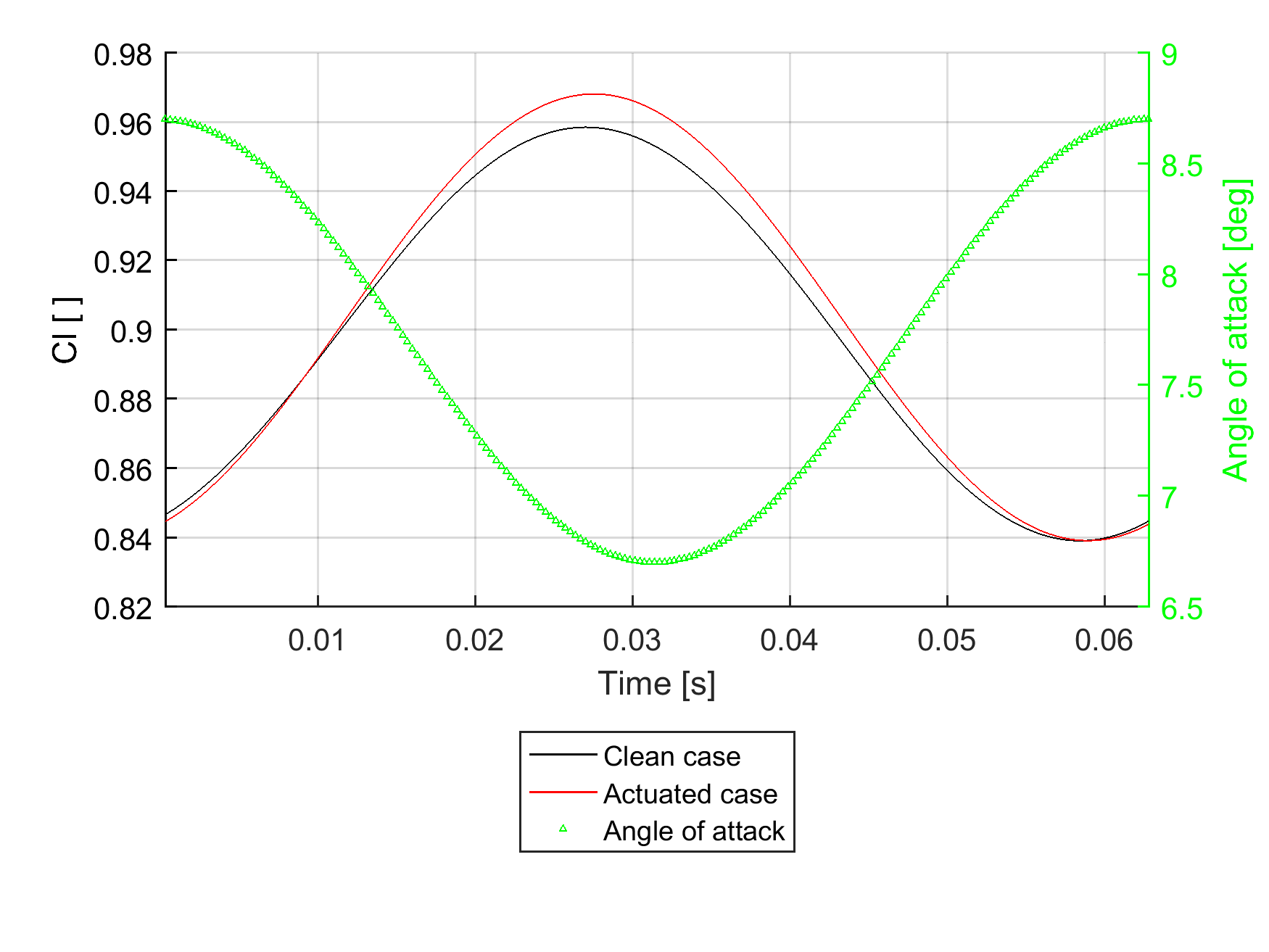

Supplement: Multimedia component 1 [file mmc1.zip › Allegati/w100_a1/Force_w100_a1_315/Lift Coefficient comparison.png]

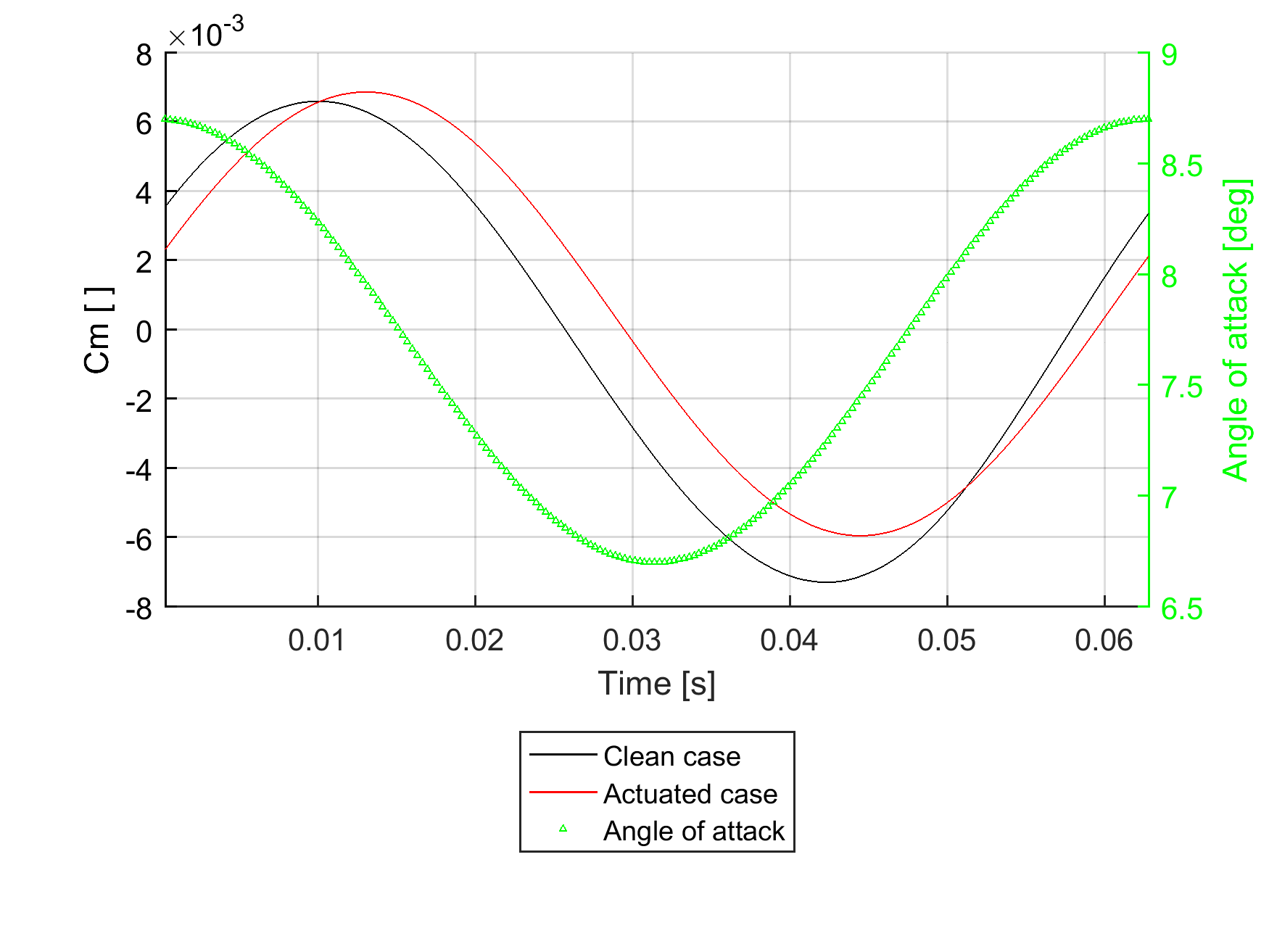

Supplement: Multimedia component 1 [file mmc1.zip › Allegati/w100_a1/Force_w100_a1_315/Moment Coefficient comparison.png]

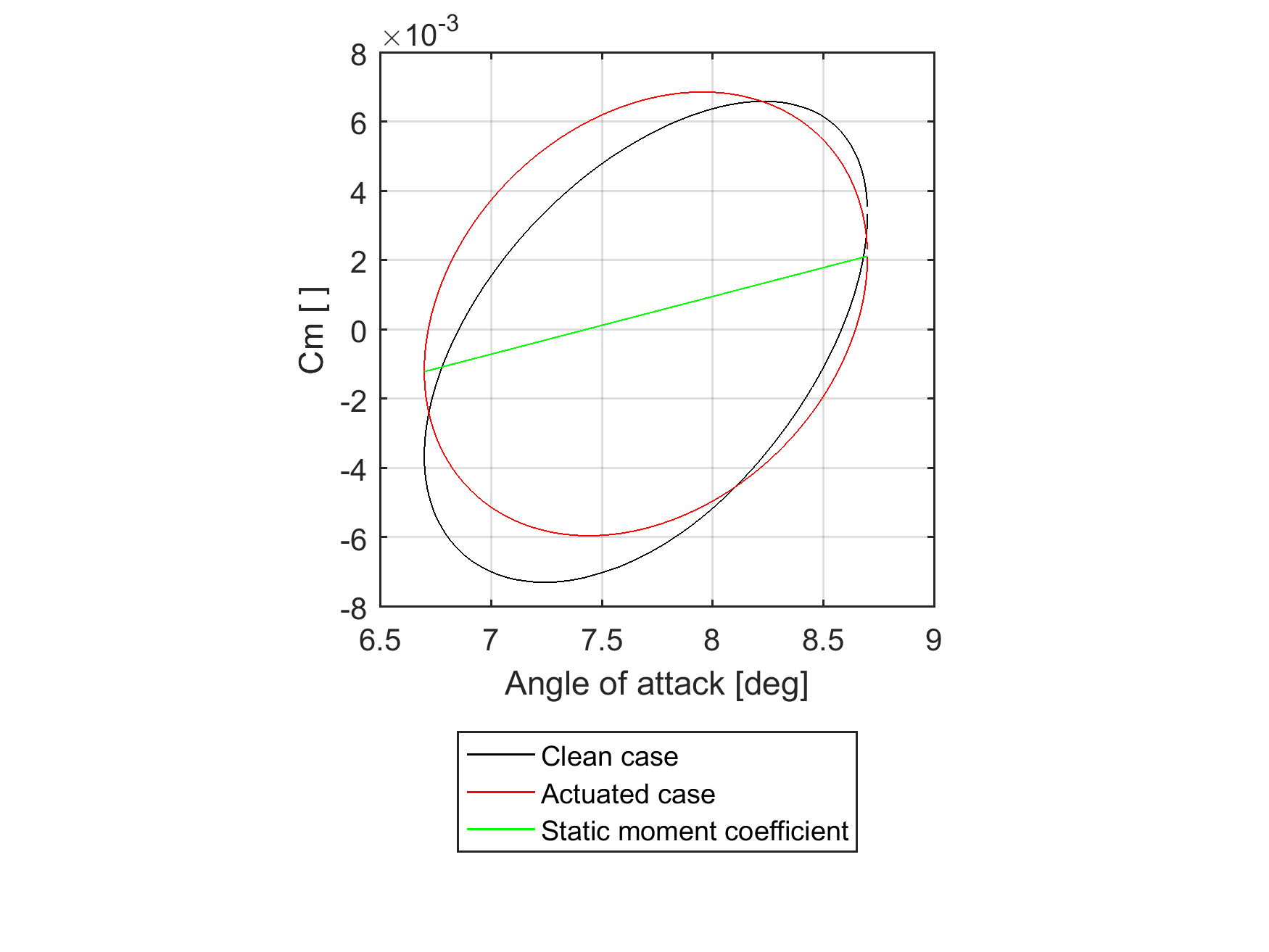

Supplement: Multimedia component 1 [file mmc1.zip › Allegati/w100_a1/Force_w100_a1_315/Moment Coefficient Hysteresis curve.png]

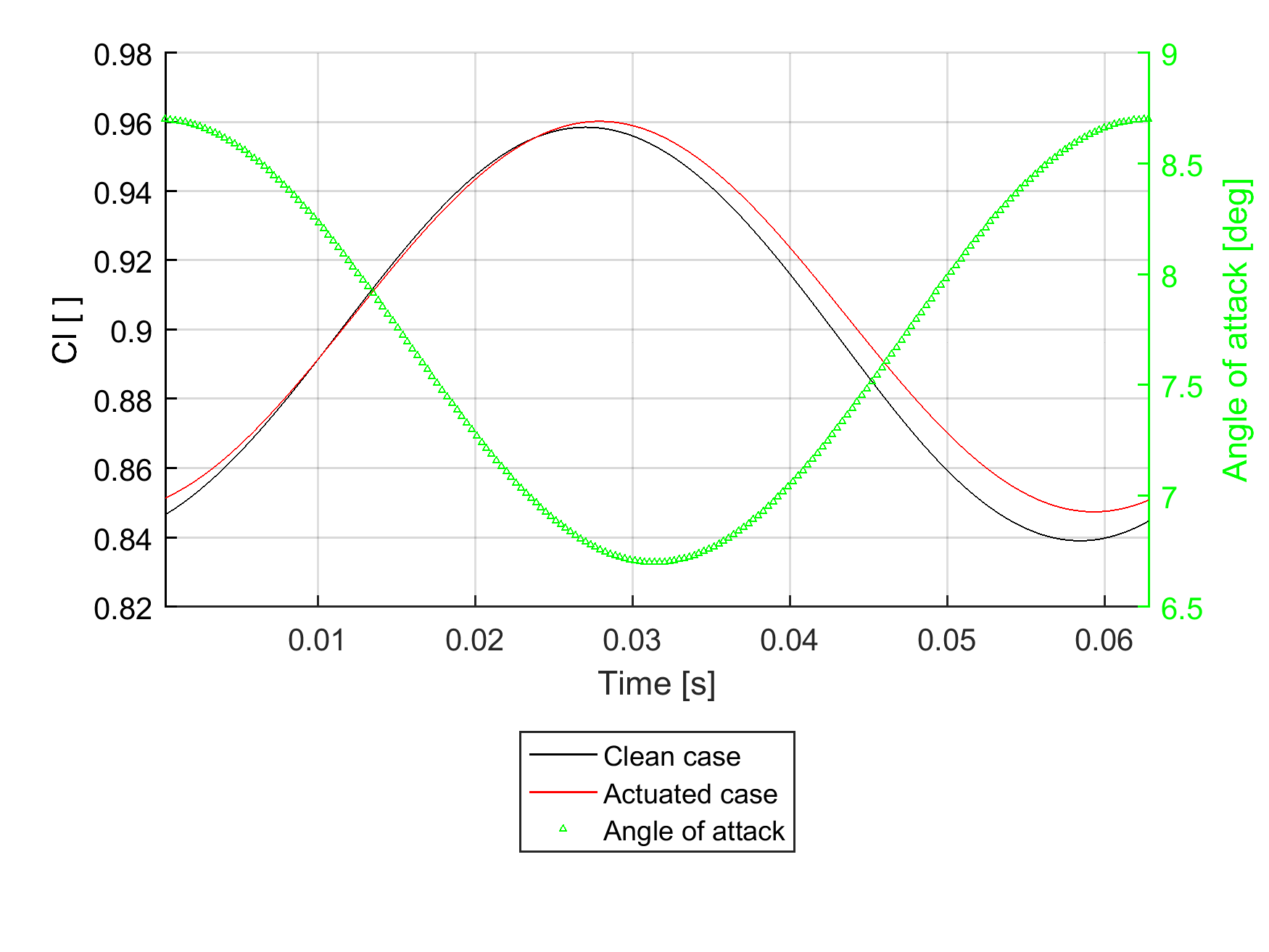

Supplement: Multimedia component 1 [file mmc1.zip › Allegati/w100_a1/Force_w100_a1_45/Lift Coefficient comparison.png]

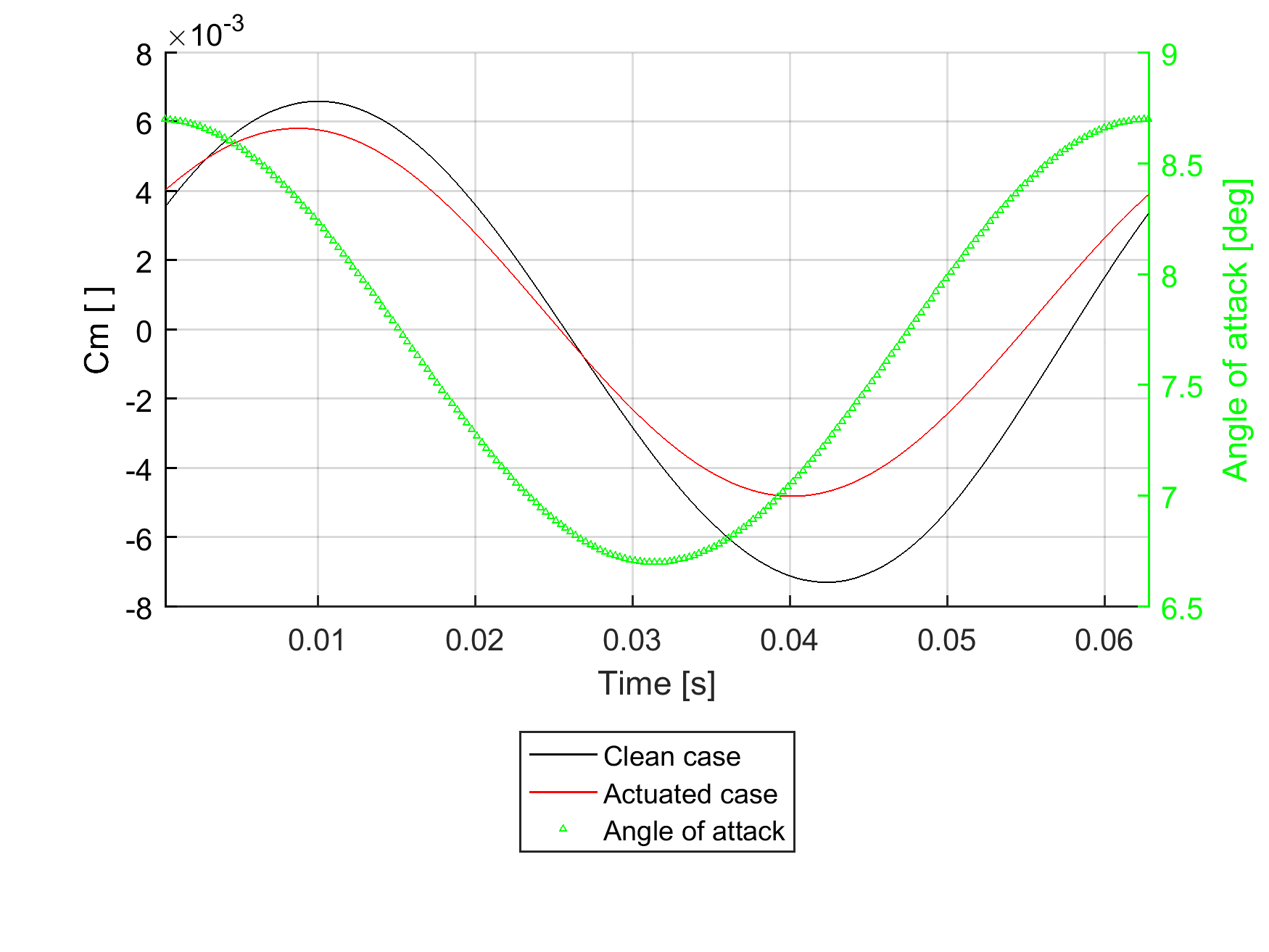

Supplement: Multimedia component 1 [file mmc1.zip › Allegati/w100_a1/Force_w100_a1_45/Moment Coefficient comparison.png]

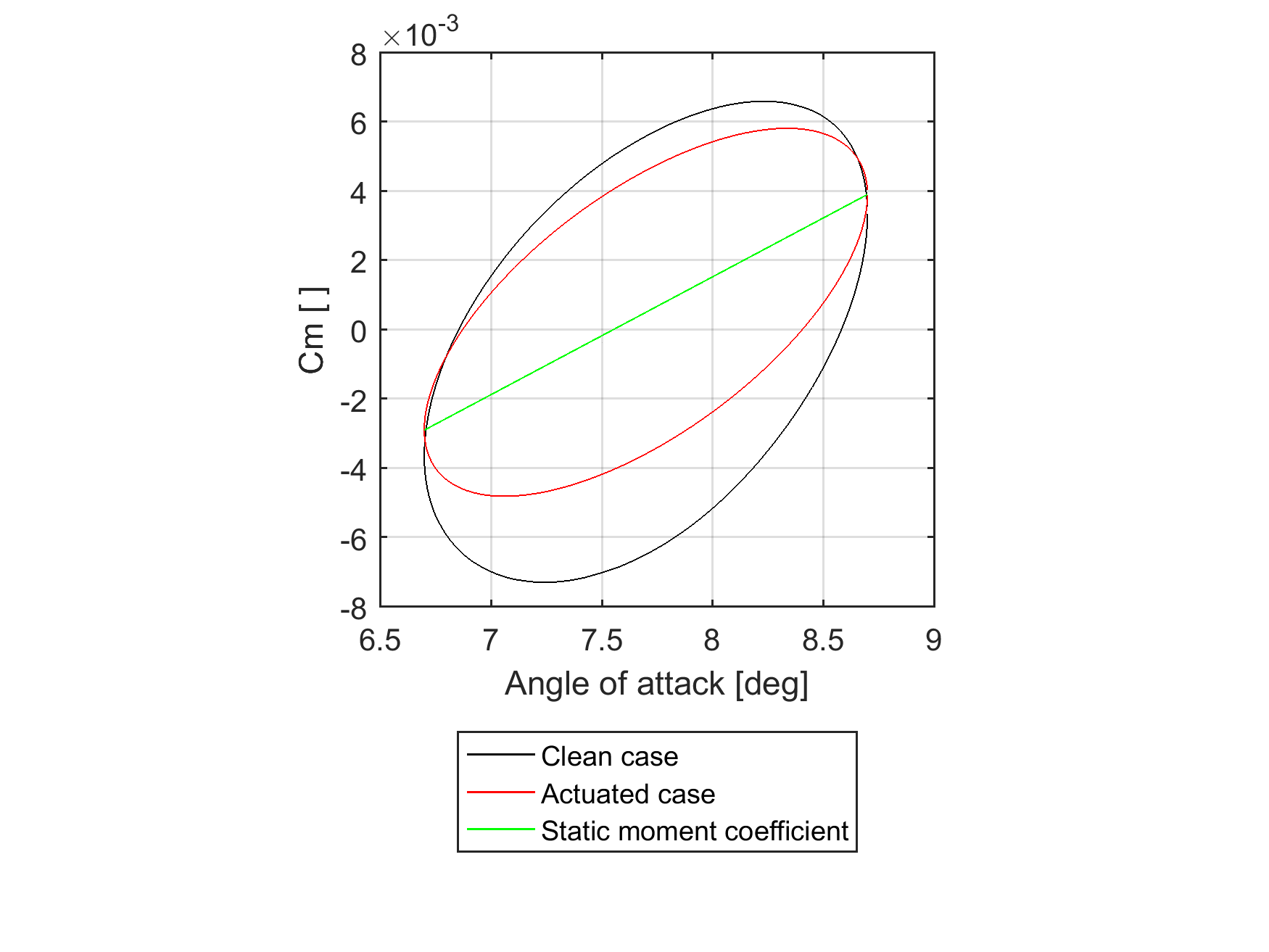

Supplement: Multimedia component 1 [file mmc1.zip › Allegati/w100_a1/Force_w100_a1_45/Moment Coefficient Hysteresis curve.png]

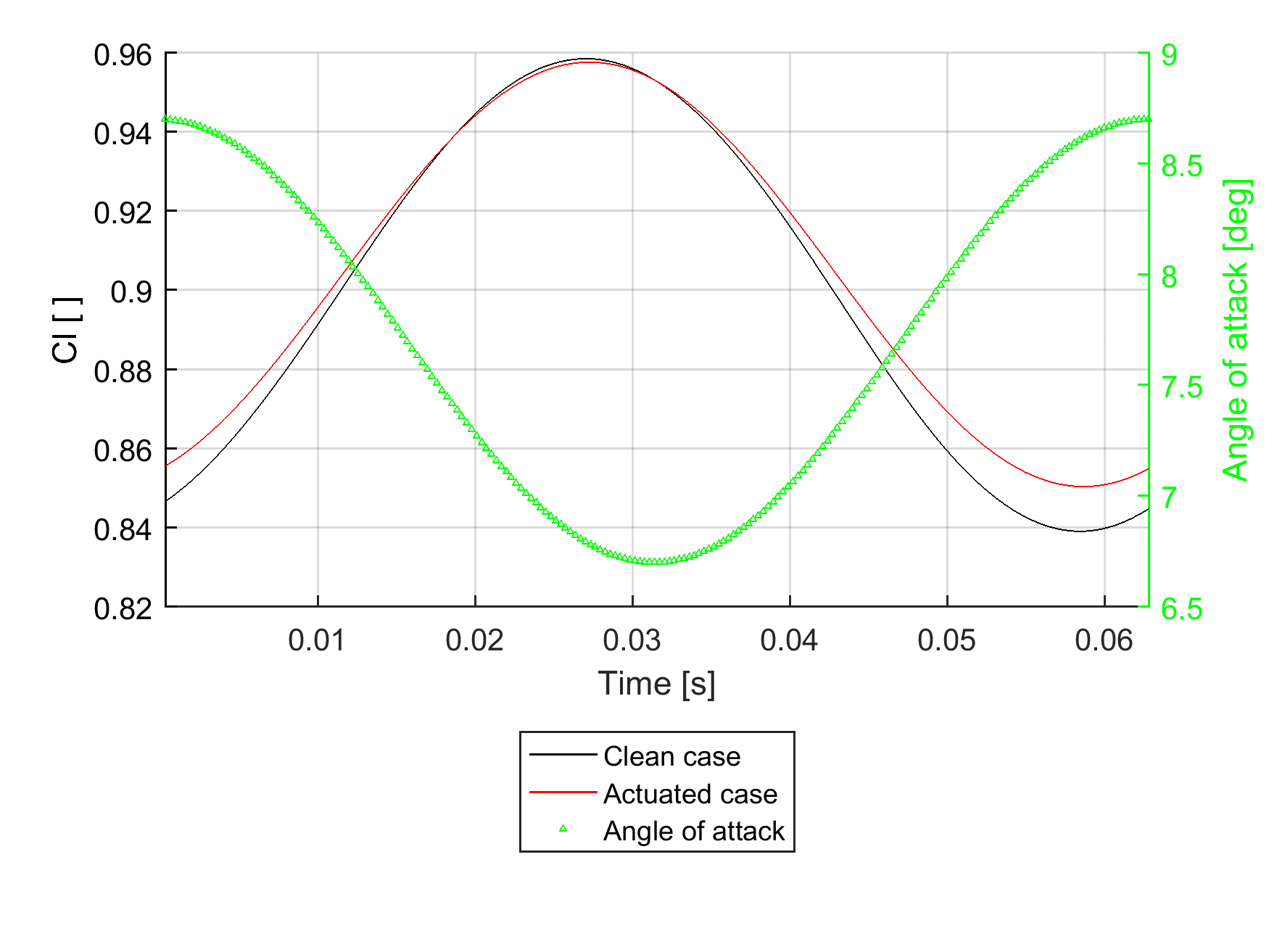

Supplement: Multimedia component 1 [file mmc1.zip › Allegati/w100_a1/Force_w100_a1_90/Lift Coefficient comparison.png]

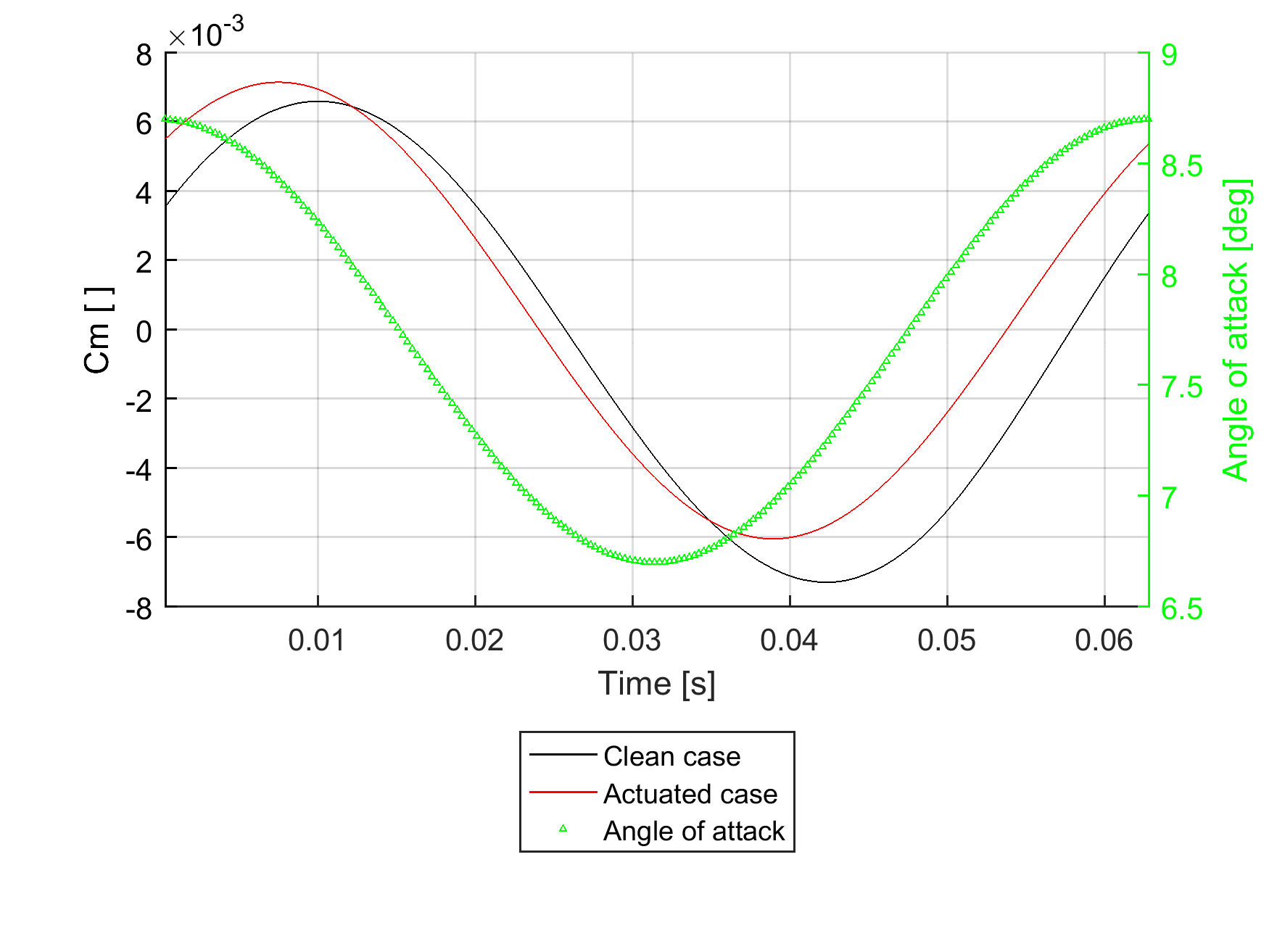

Supplement: Multimedia component 1 [file mmc1.zip › Allegati/w100_a1/Force_w100_a1_90/Moment Coefficient comparison.png]

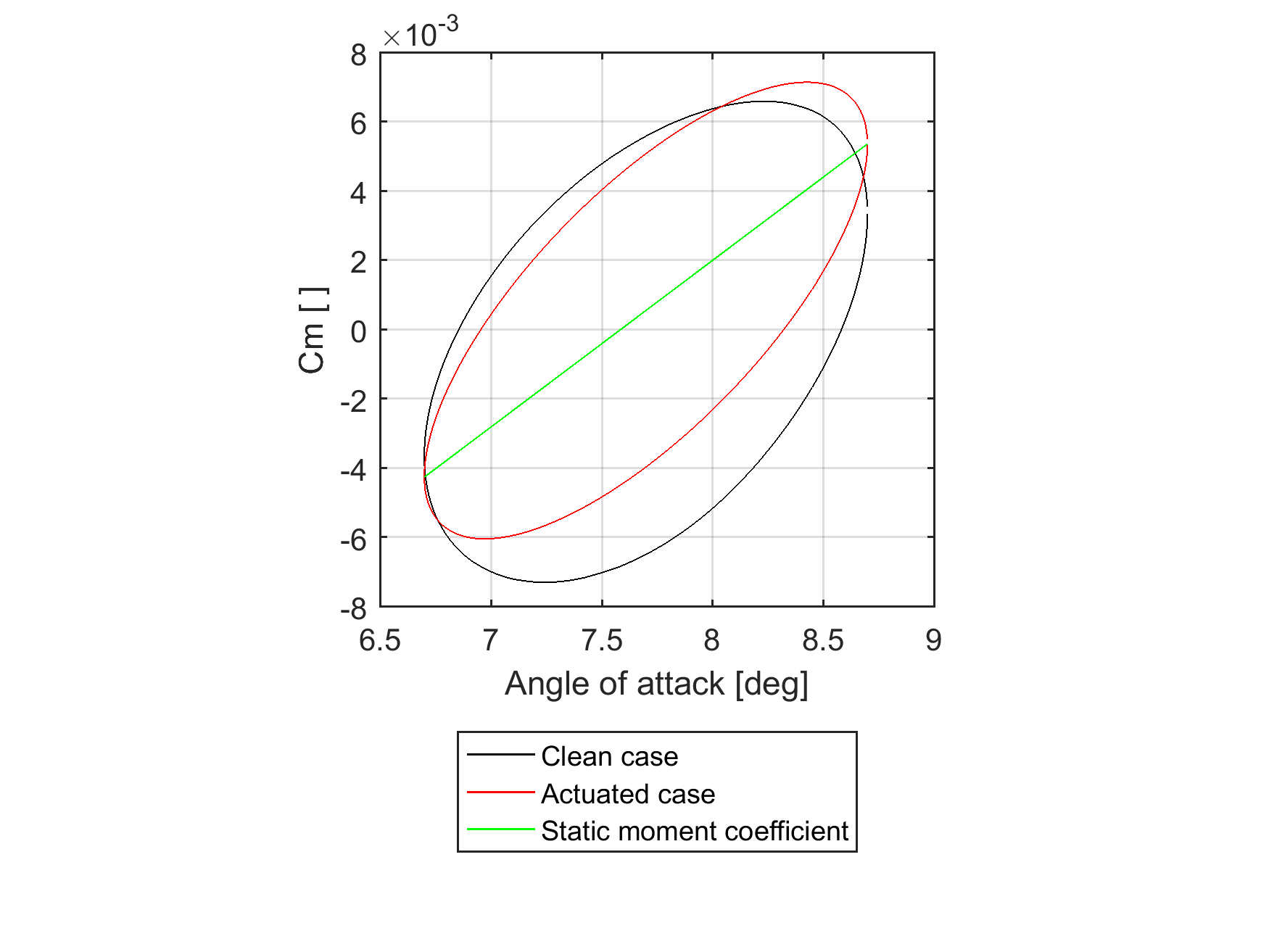

Supplement: Multimedia component 1 [file mmc1.zip › Allegati/w100_a1/Force_w100_a1_90/Moment Coefficient Hysteresis curve.png]

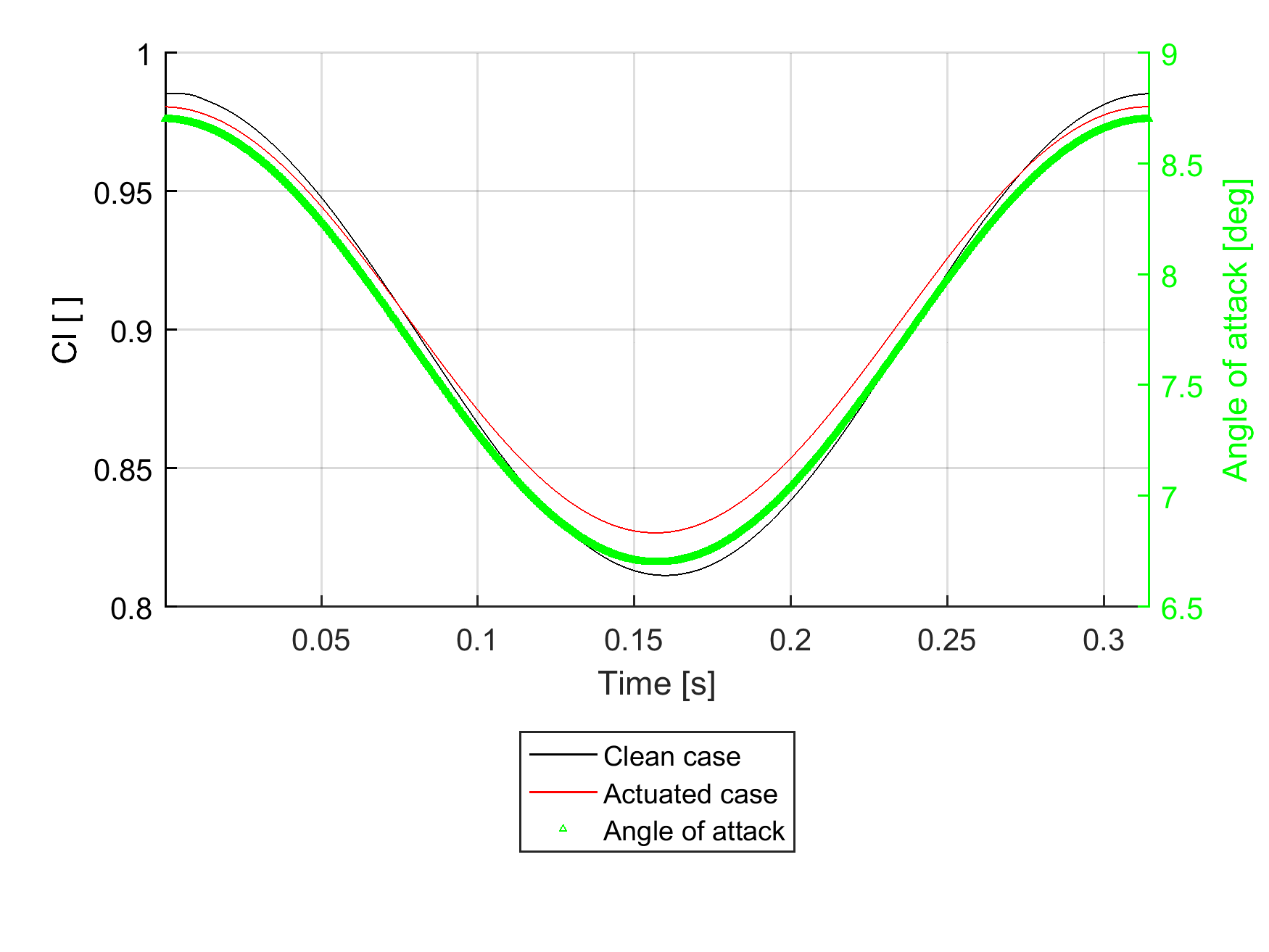

Supplement: Multimedia component 1 [file mmc1.zip › Allegati/w20_a1/Force_w20_a1_0/Lift Coefficient comparison.png]

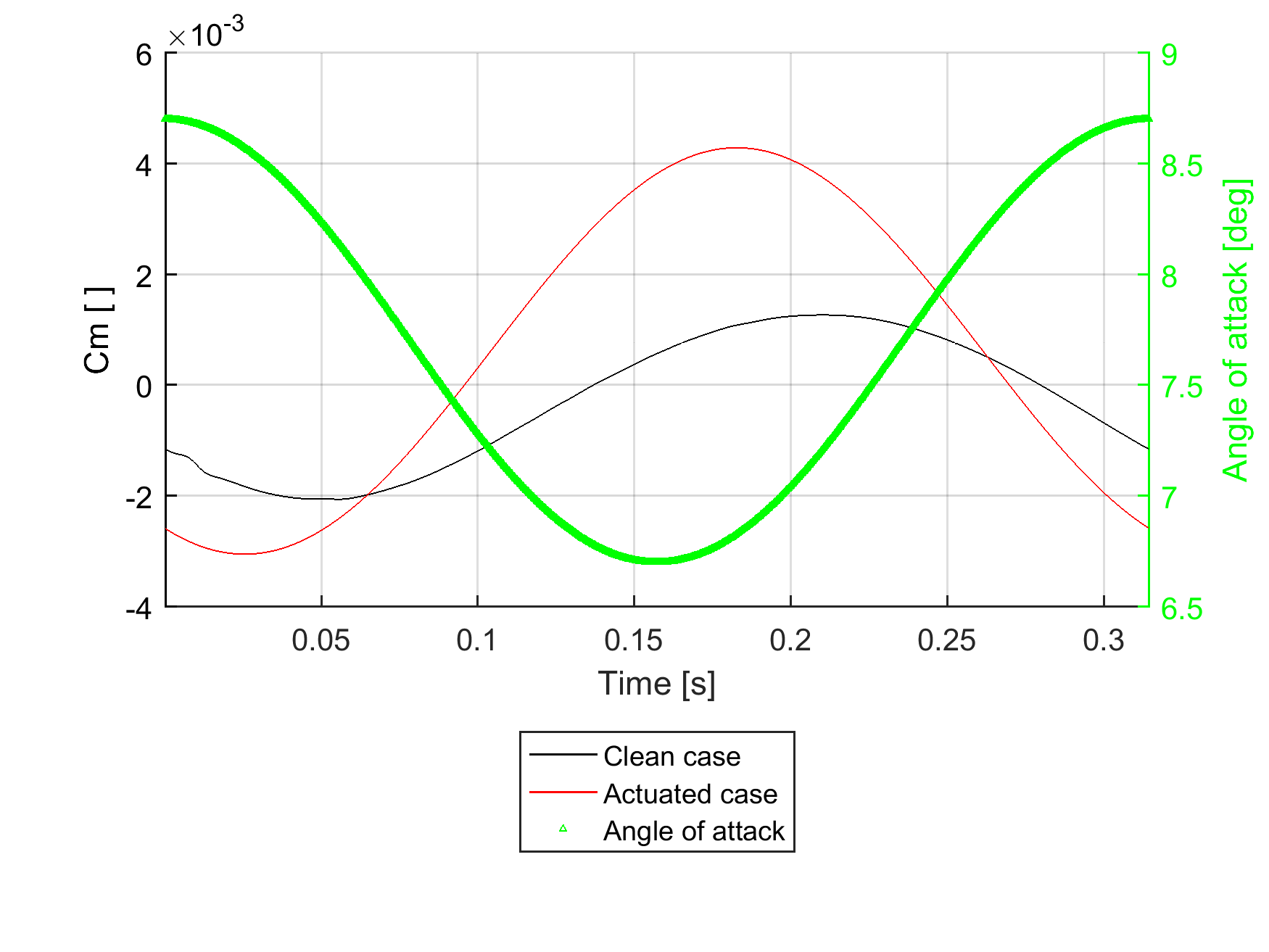

Supplement: Multimedia component 1 [file mmc1.zip › Allegati/w20_a1/Force_w20_a1_0/Moment Coefficient comparison.png]

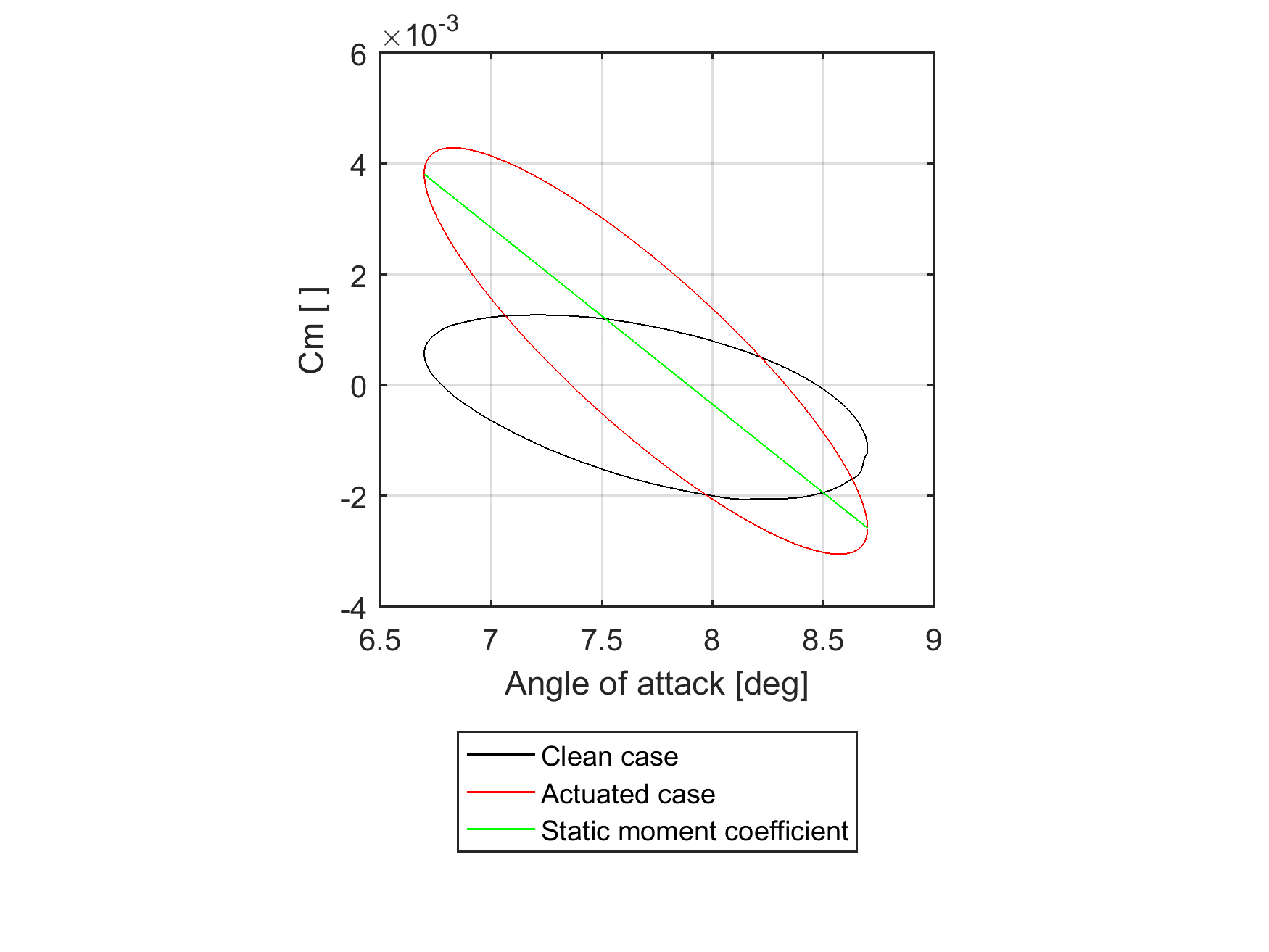

Supplement: Multimedia component 1 [file mmc1.zip › Allegati/w20_a1/Force_w20_a1_0/Moment Coefficient Hysteresis curve.png]

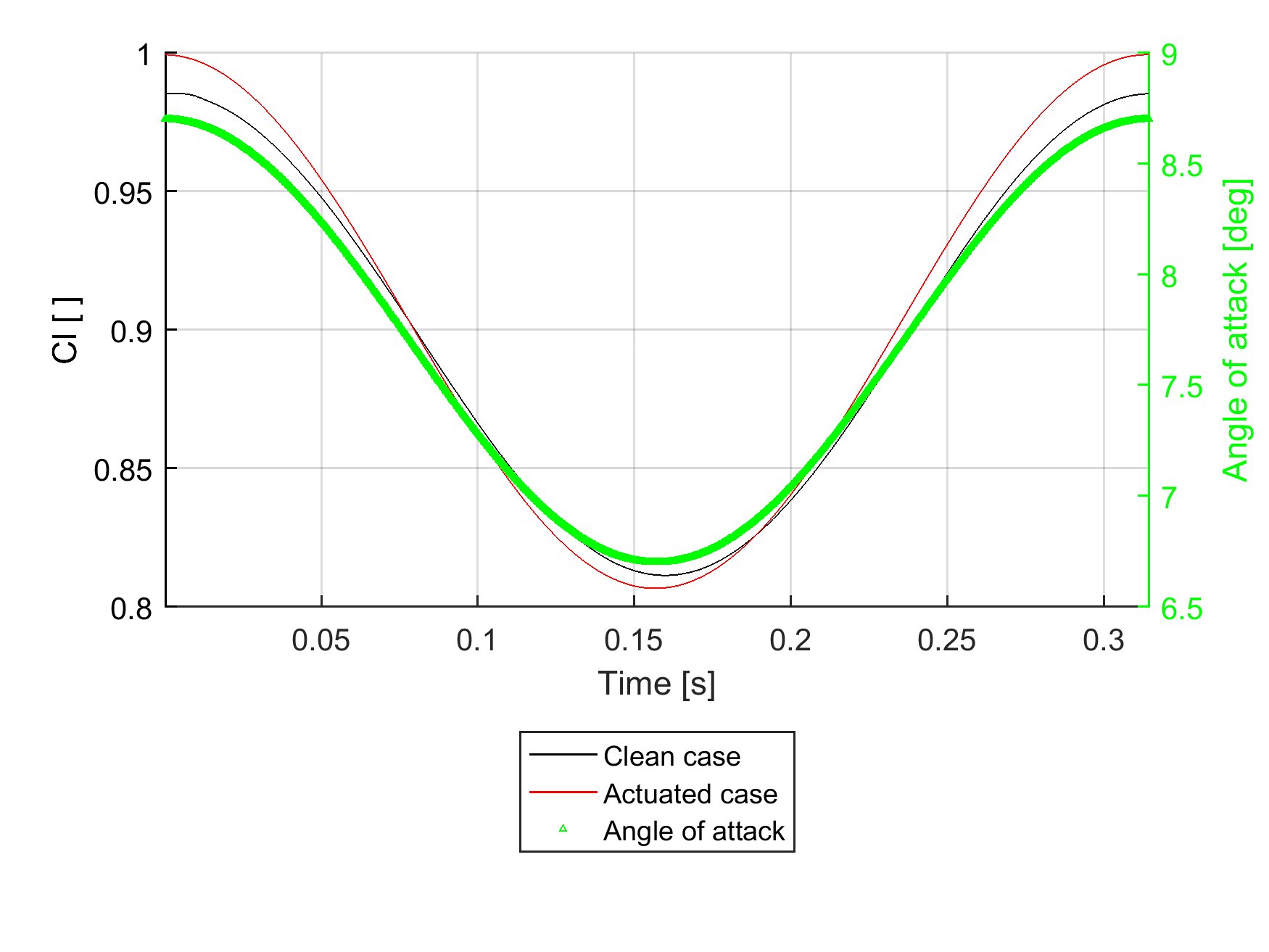

Supplement: Multimedia component 1 [file mmc1.zip › Allegati/w20_a1/Force_w20_a1_135/Lift Coefficient comparison.png]

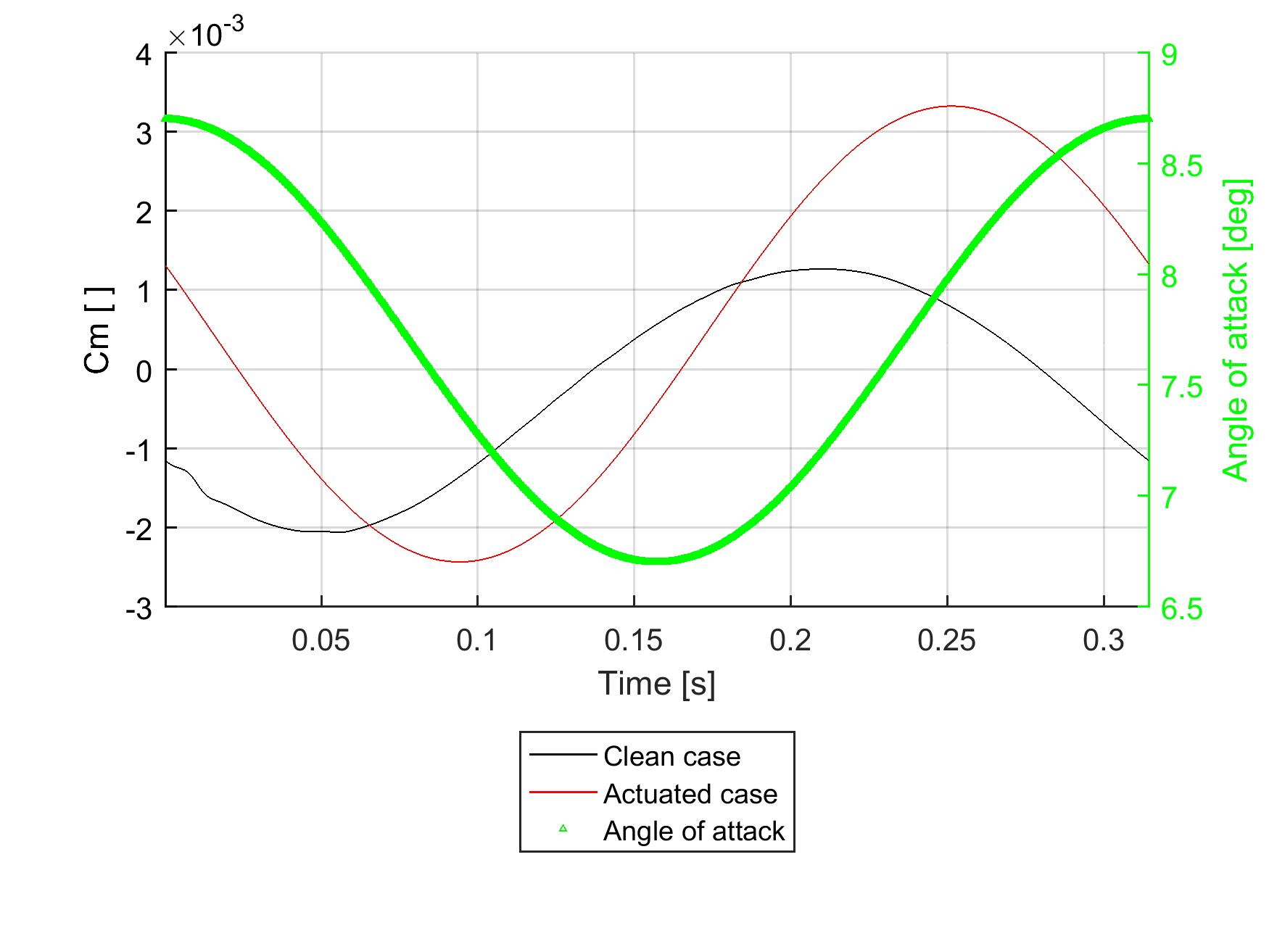

Supplement: Multimedia component 1 [file mmc1.zip › Allegati/w20_a1/Force_w20_a1_135/Moment Coefficient comparison.png]

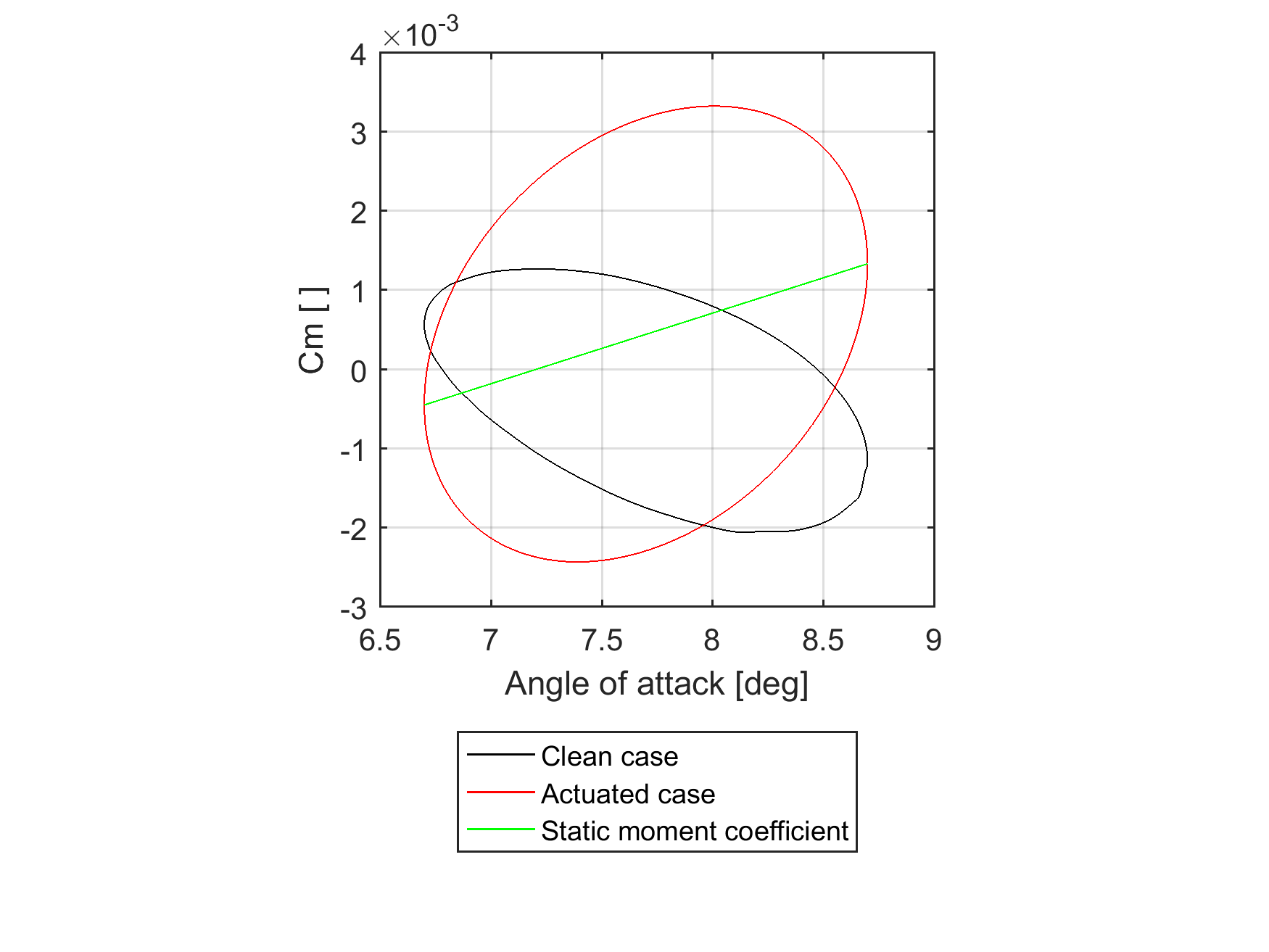

Supplement: Multimedia component 1 [file mmc1.zip › Allegati/w20_a1/Force_w20_a1_135/Moment Coefficient Hysteresis curve.png]

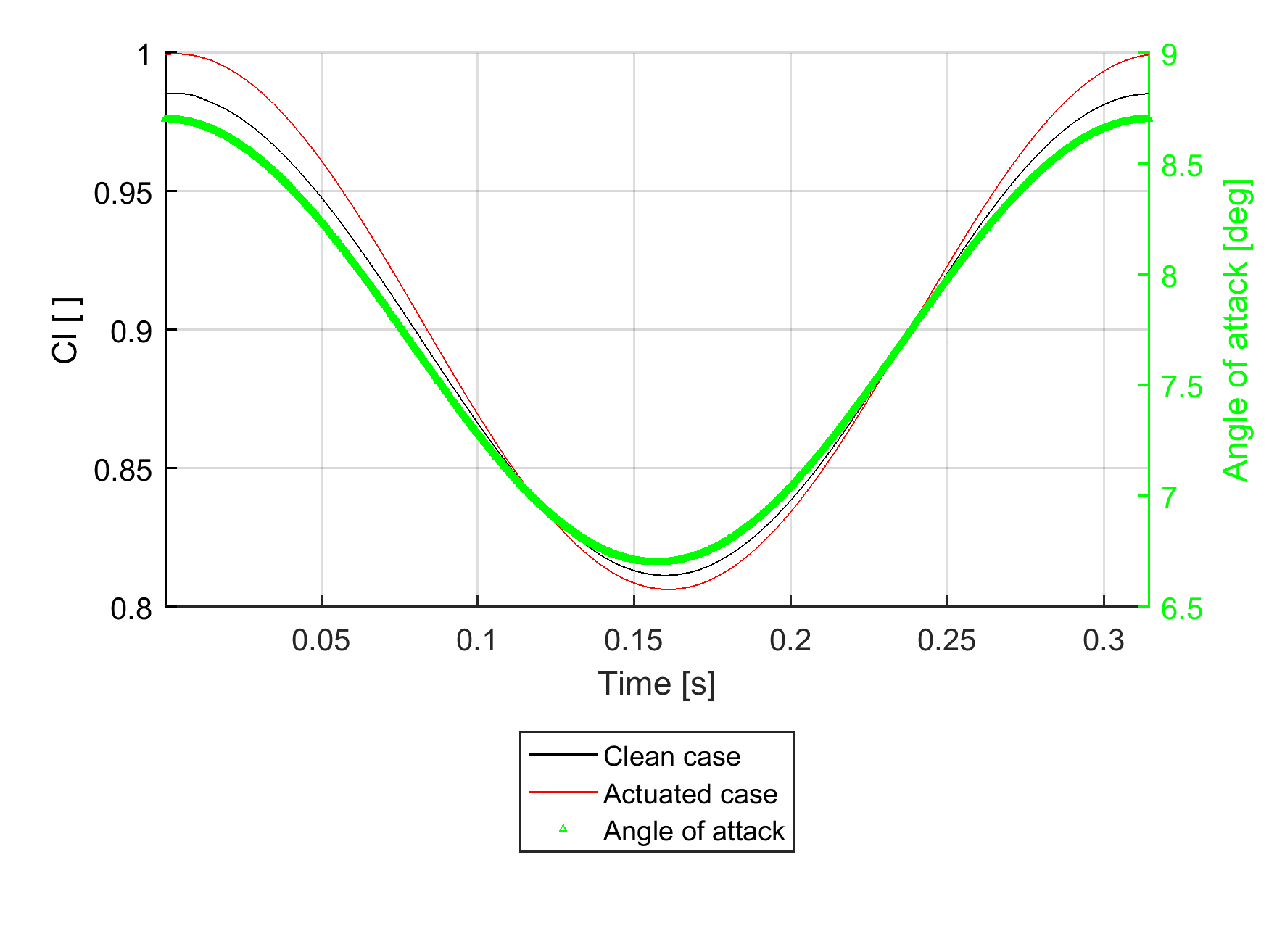

Supplement: Multimedia component 1 [file mmc1.zip › Allegati/w20_a1/Force_w20_a1_180/Lift Coefficient comparison.png]

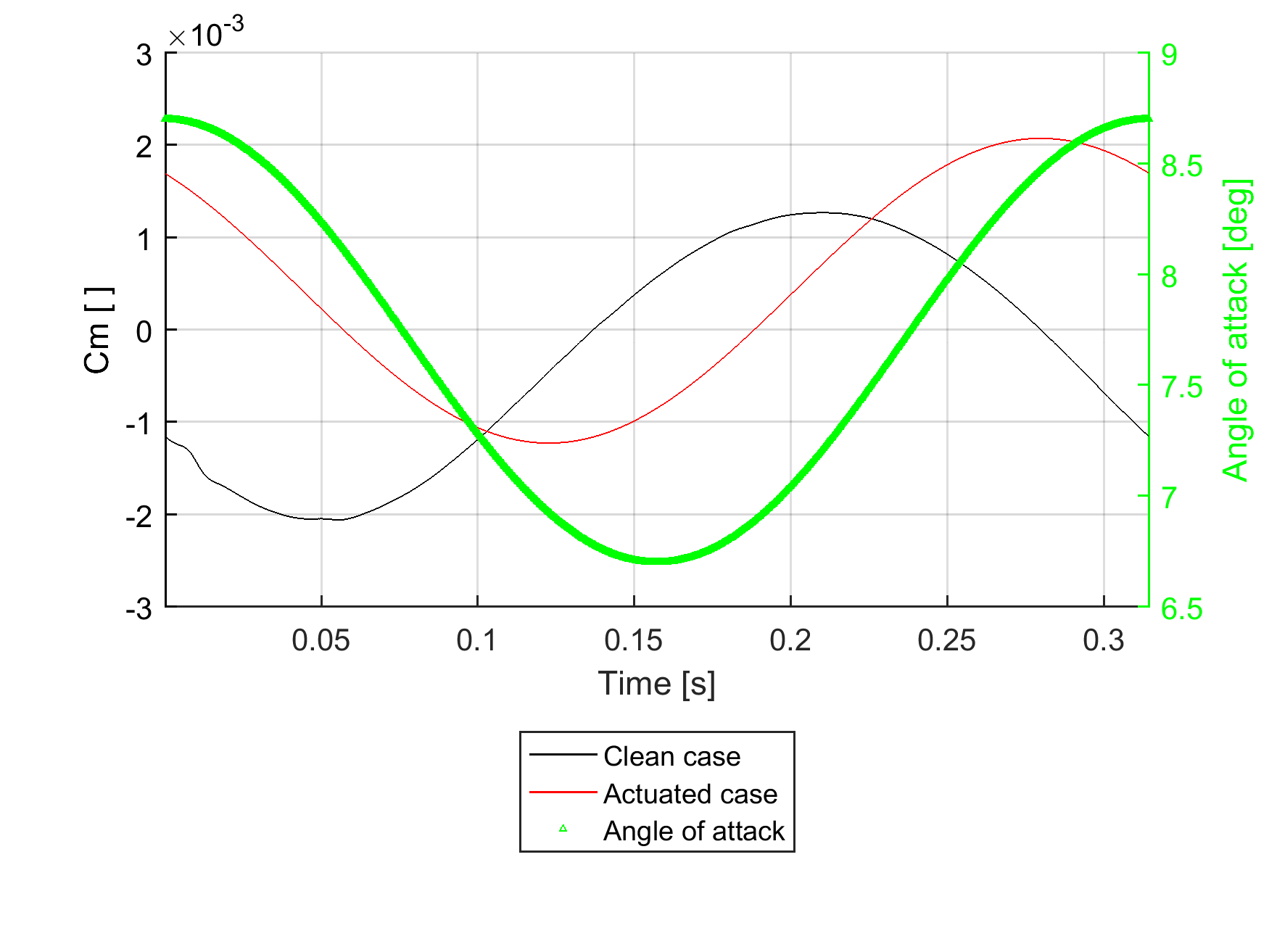

Supplement: Multimedia component 1 [file mmc1.zip › Allegati/w20_a1/Force_w20_a1_180/Moment Coefficient comparison.png]

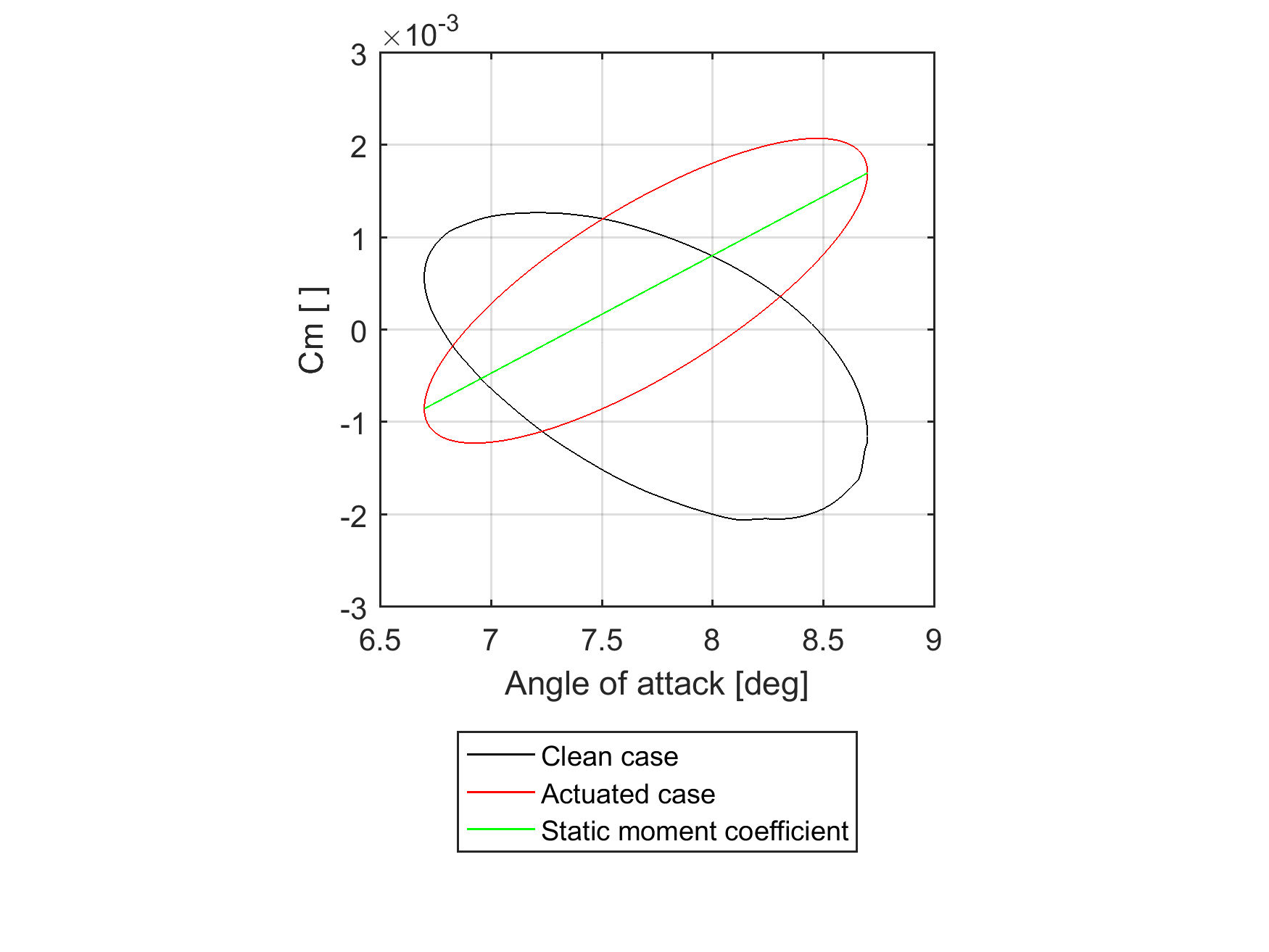

Supplement: Multimedia component 1 [file mmc1.zip › Allegati/w20_a1/Force_w20_a1_180/Moment Coefficient Hysteresis curve.png]

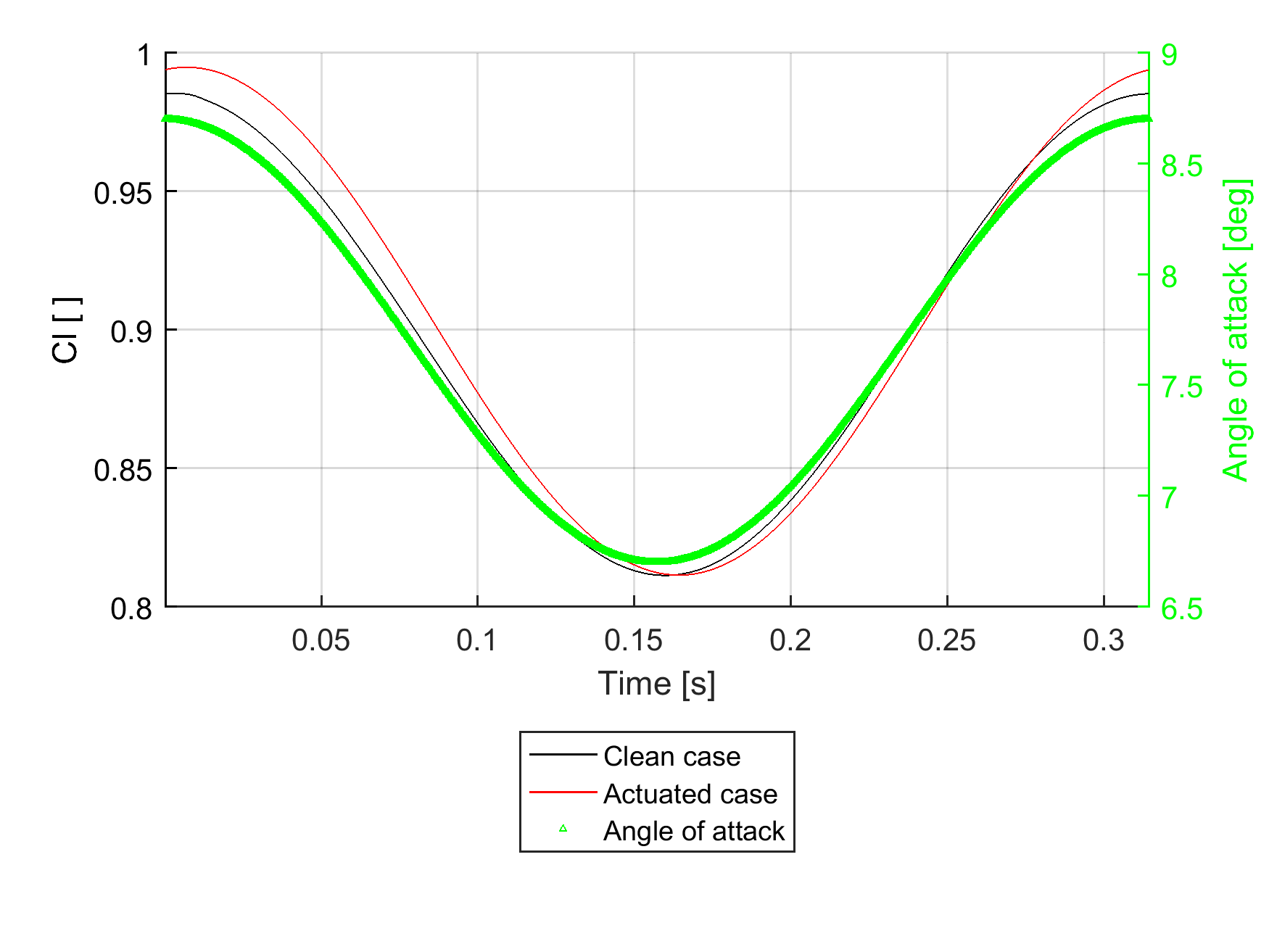

Supplement: Multimedia component 1 [file mmc1.zip › Allegati/w20_a1/Force_w20_a1_225/Lift Coefficient comparison.png]

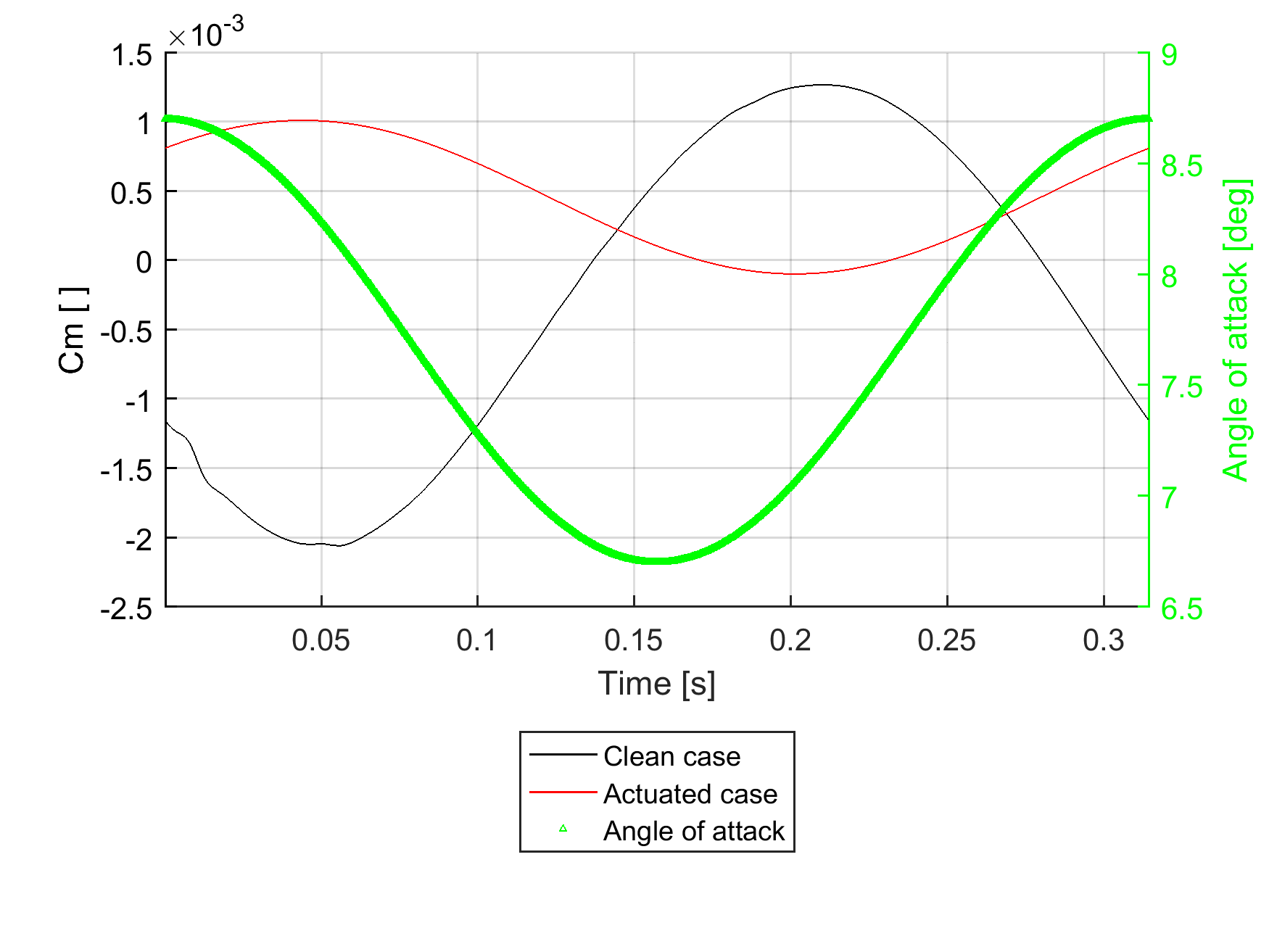

Supplement: Multimedia component 1 [file mmc1.zip › Allegati/w20_a1/Force_w20_a1_225/Moment Coefficient comparison.png]

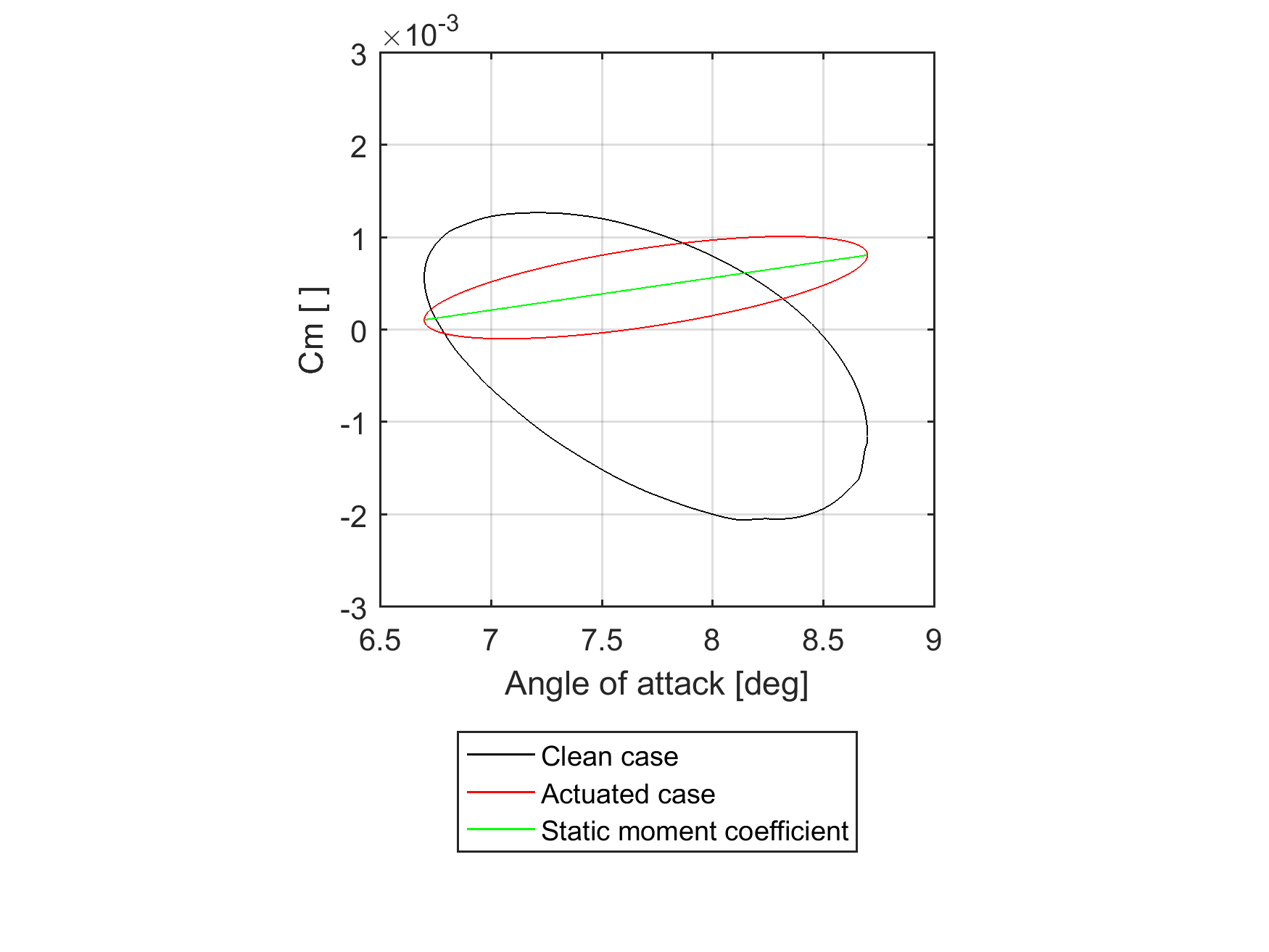

Supplement: Multimedia component 1 [file mmc1.zip › Allegati/w20_a1/Force_w20_a1_225/Moment Coefficient Hysteresis curve.png]

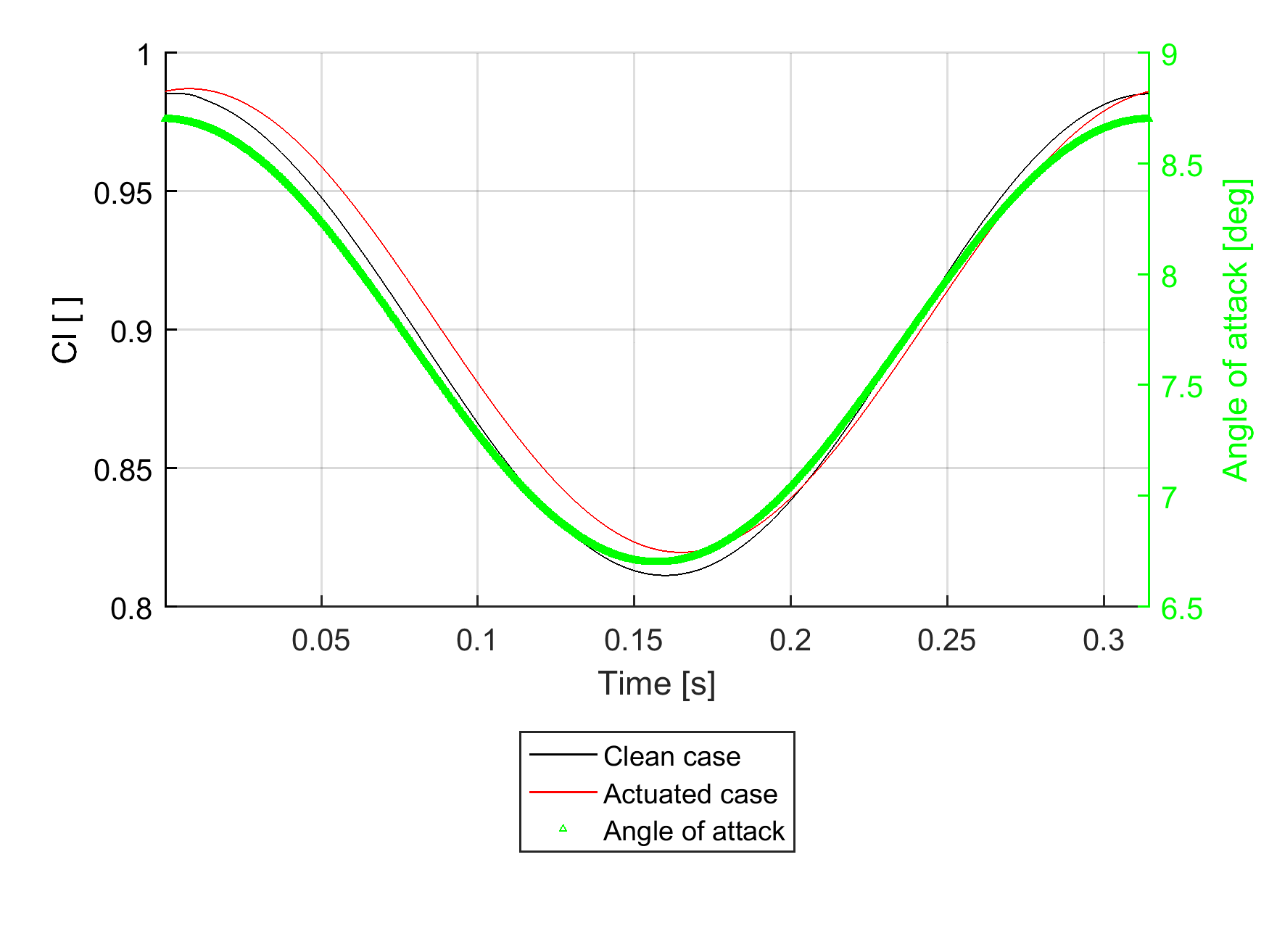

Supplement: Multimedia component 1 [file mmc1.zip › Allegati/w20_a1/Force_w20_a1_270/Lift Coefficient comparison.png]

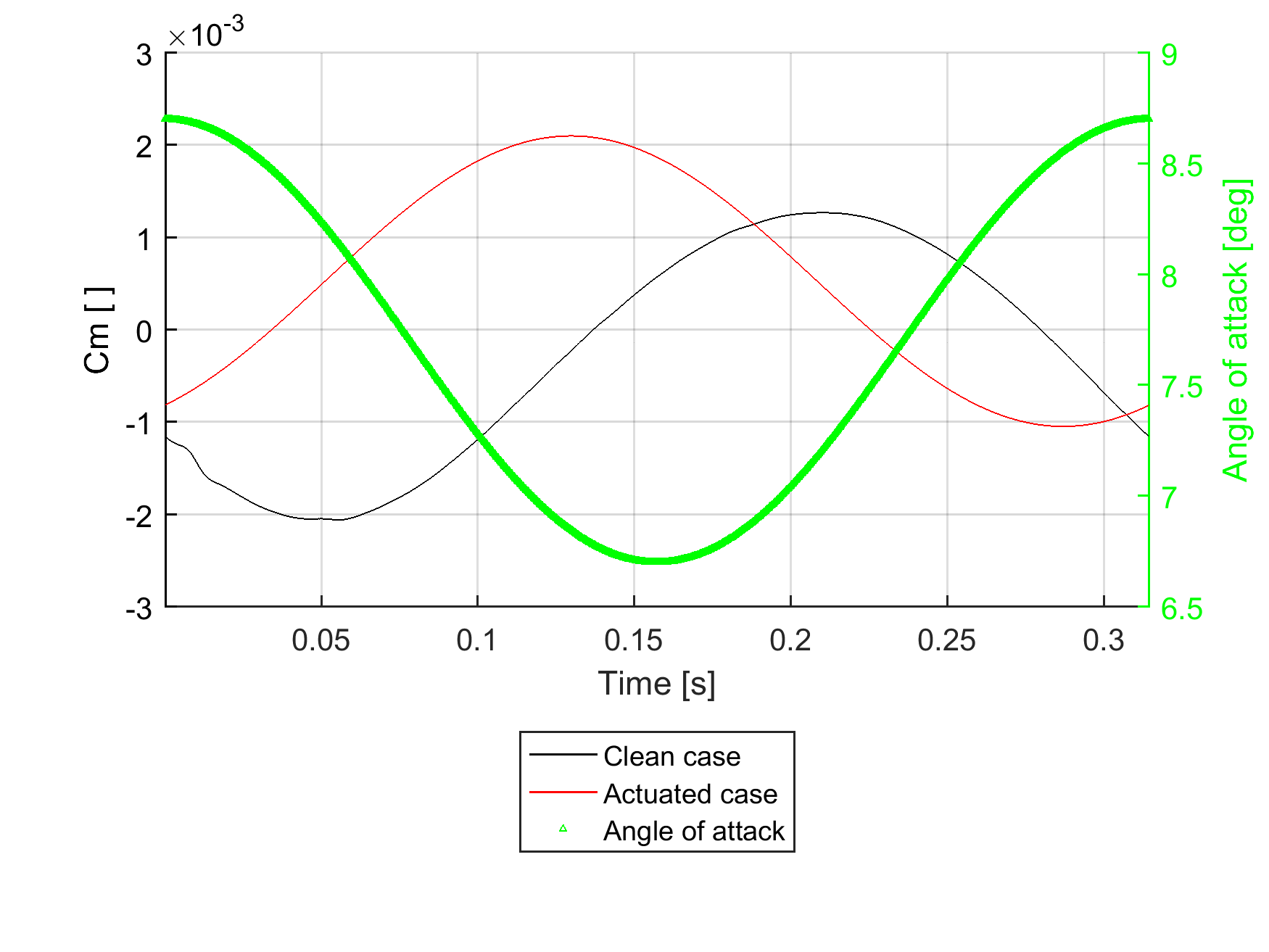

Supplement: Multimedia component 1 [file mmc1.zip › Allegati/w20_a1/Force_w20_a1_270/Moment Coefficient comparison.png]

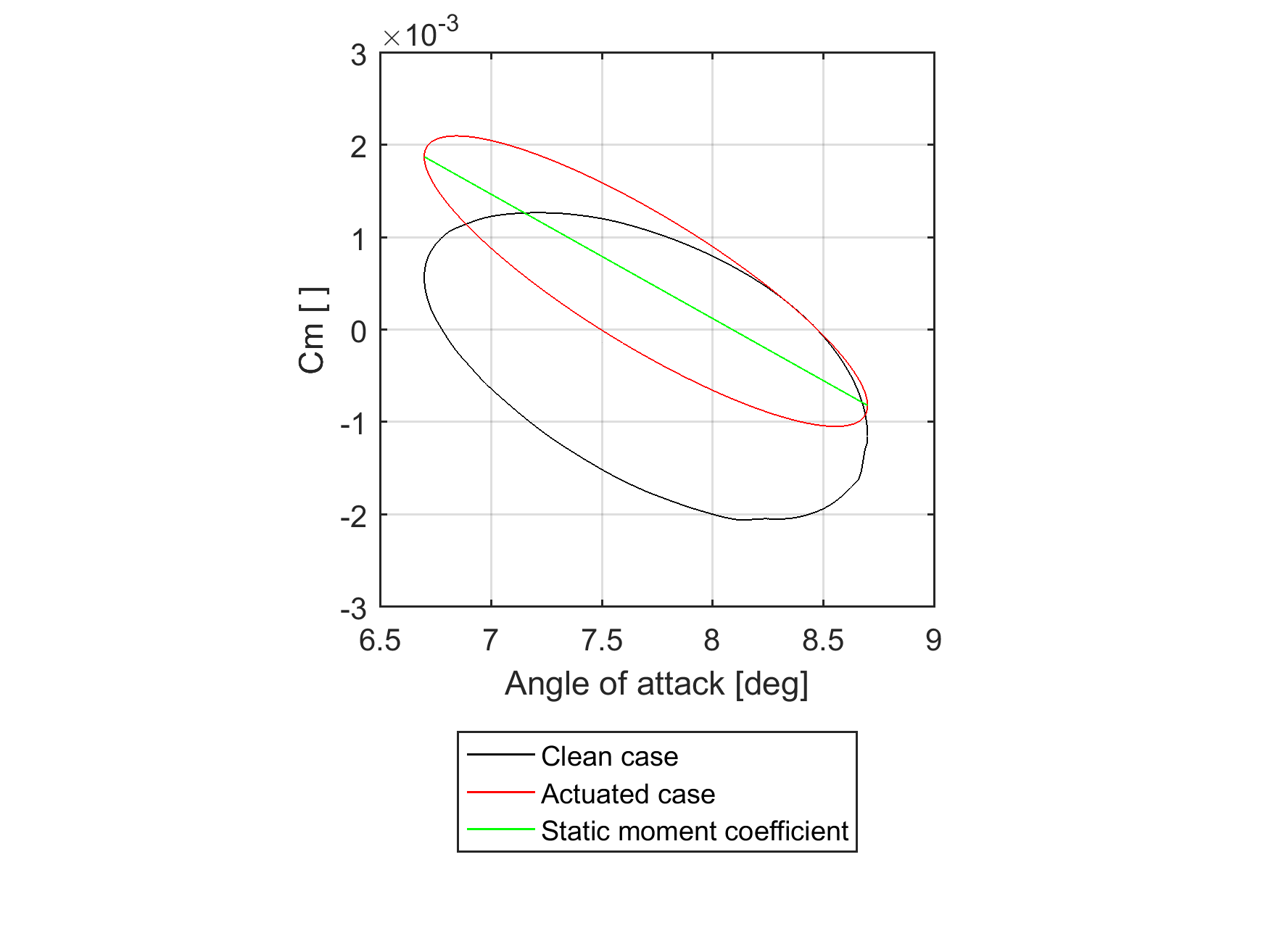

Supplement: Multimedia component 1 [file mmc1.zip › Allegati/w20_a1/Force_w20_a1_270/Moment Coefficient Hysteresis curve.png]

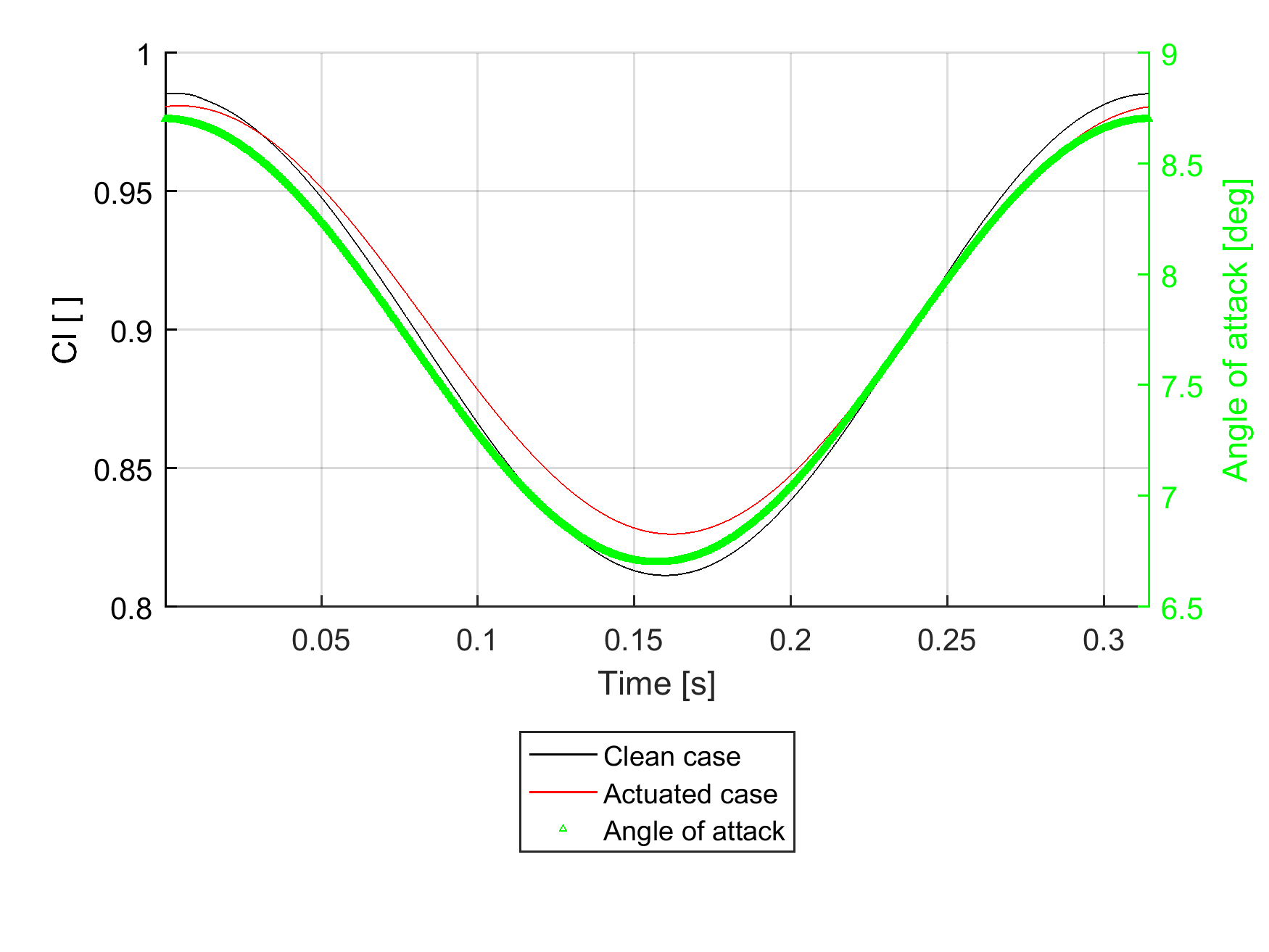

Supplement: Multimedia component 1 [file mmc1.zip › Allegati/w20_a1/Force_w20_a1_315/Lift Coefficient comparison.png]

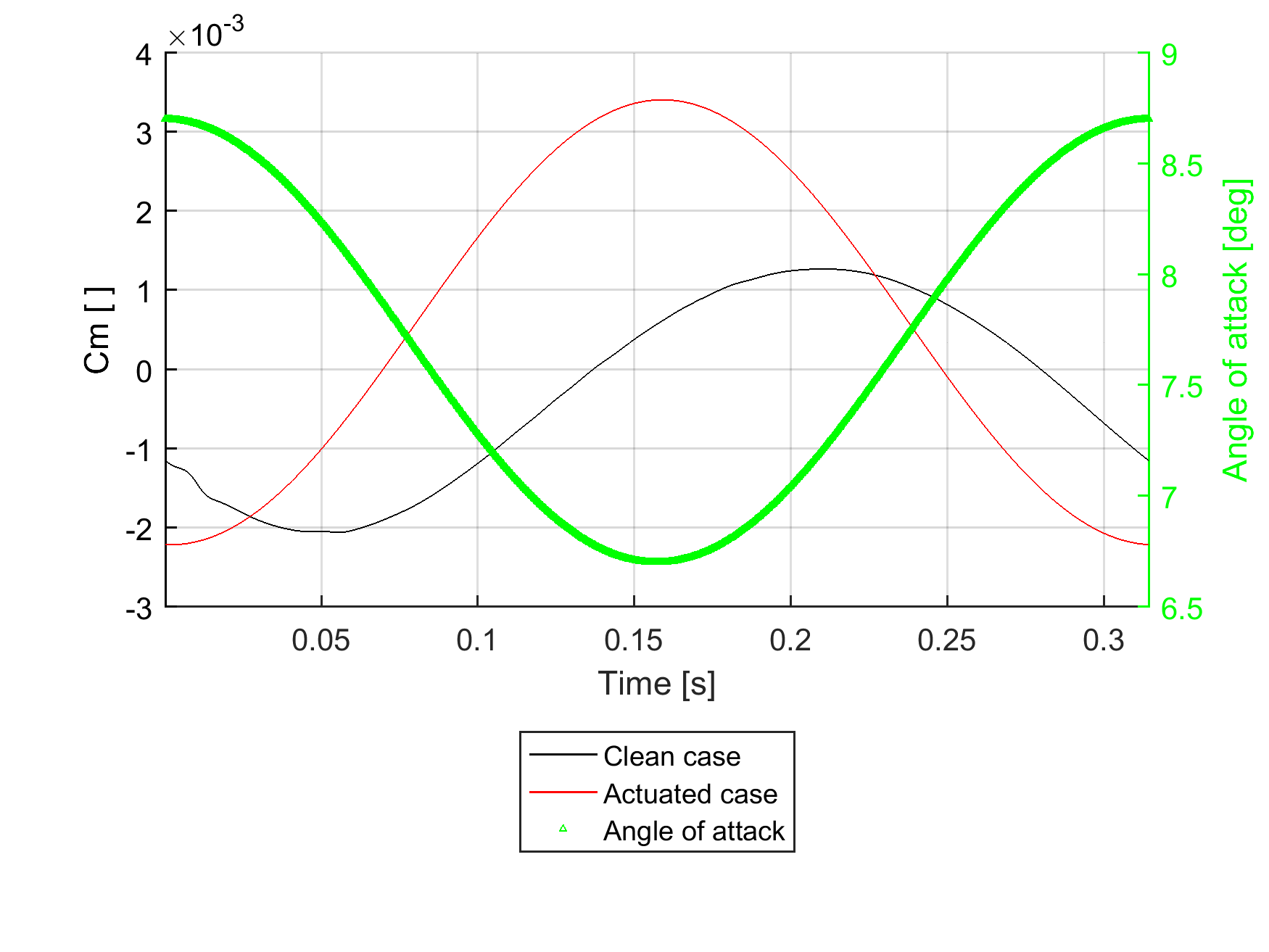

Supplement: Multimedia component 1 [file mmc1.zip › Allegati/w20_a1/Force_w20_a1_315/Moment Coefficient comparison.png]

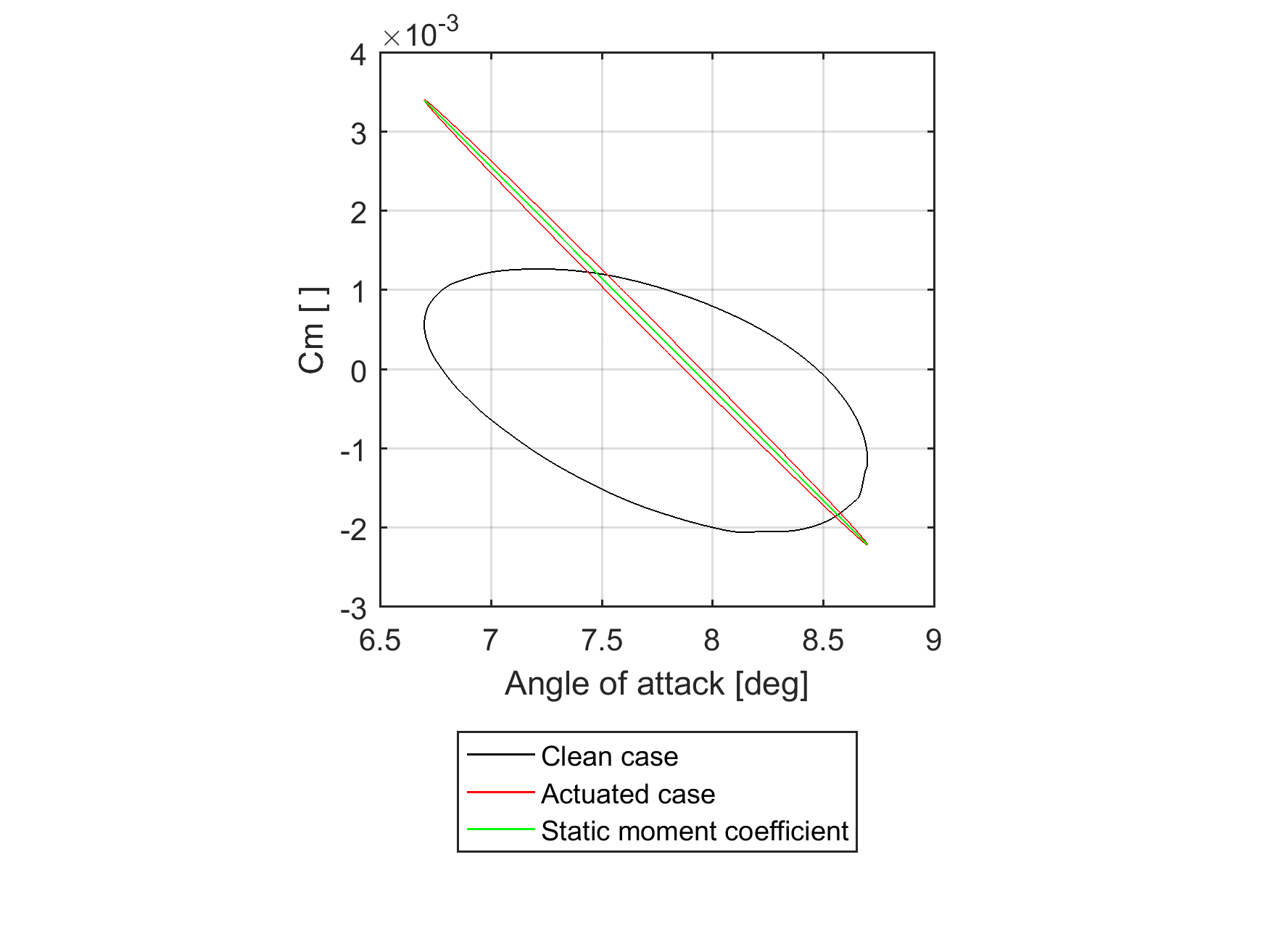

Supplement: Multimedia component 1 [file mmc1.zip › Allegati/w20_a1/Force_w20_a1_315/Moment Coefficient Hysteresis curve.png]

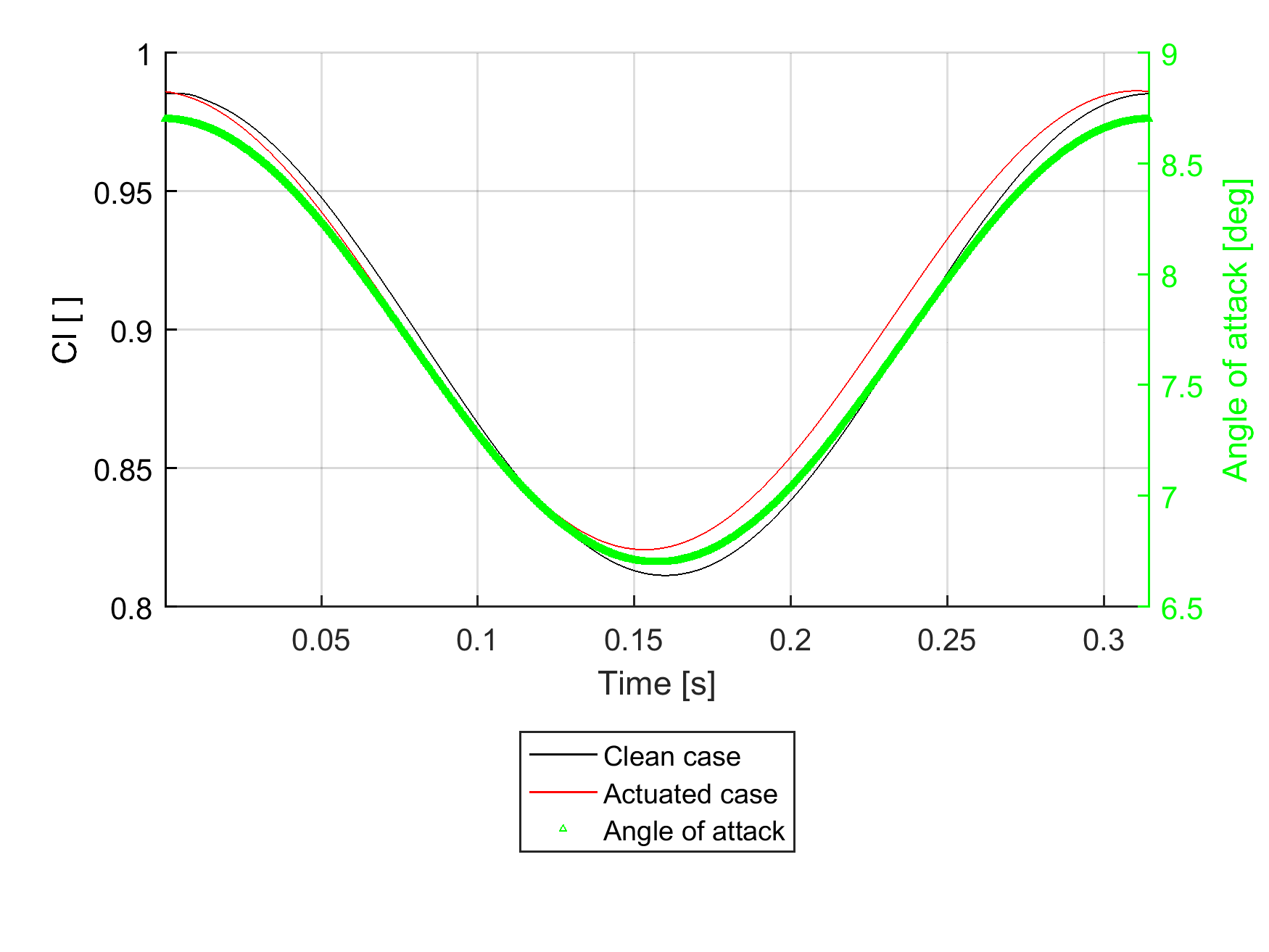

Supplement: Multimedia component 1 [file mmc1.zip › Allegati/w20_a1/Force_w20_a1_45/Lift Coefficient comparison.png]

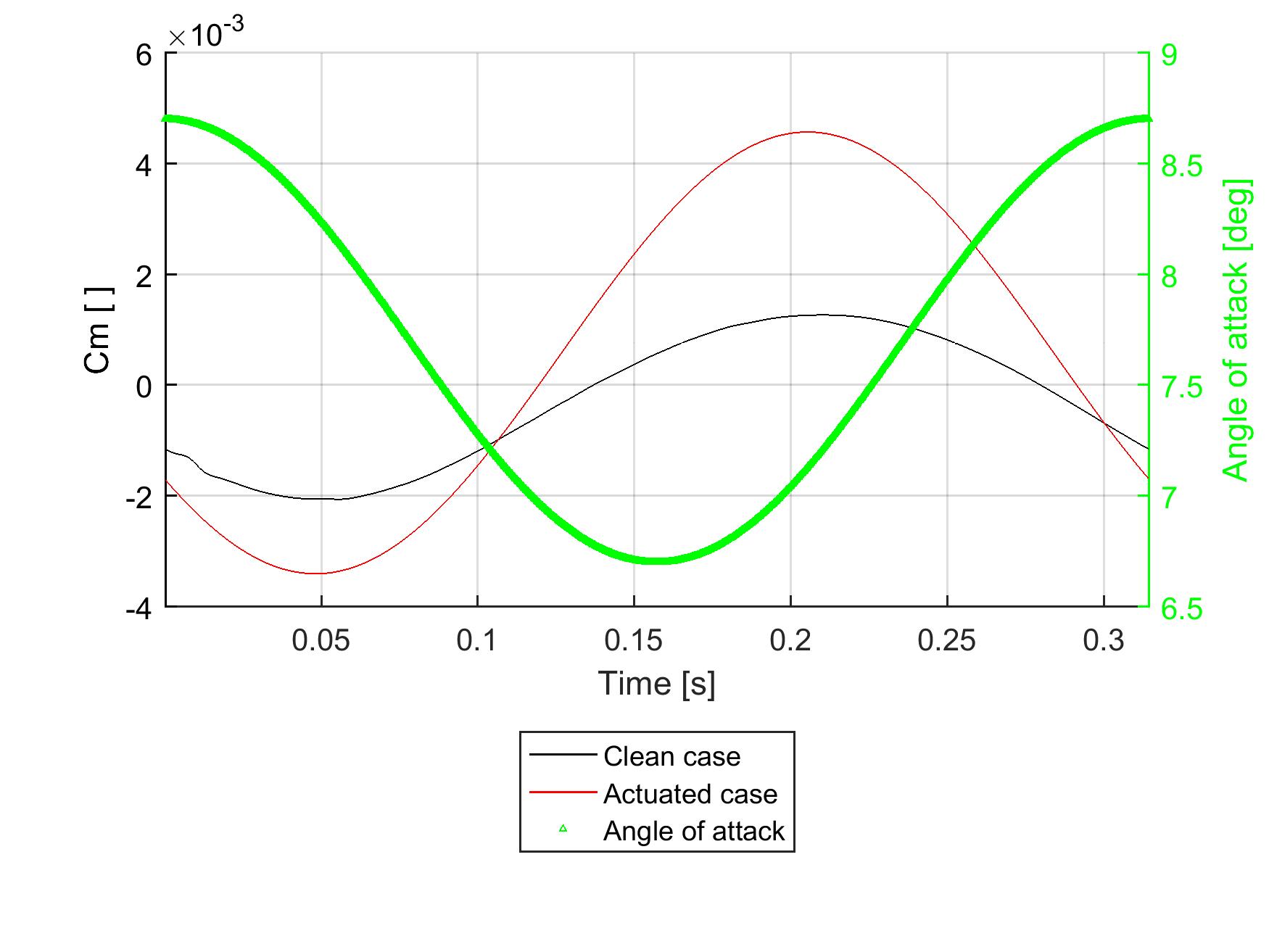

Supplement: Multimedia component 1 [file mmc1.zip › Allegati/w20_a1/Force_w20_a1_45/Moment Coefficient comparison.png]

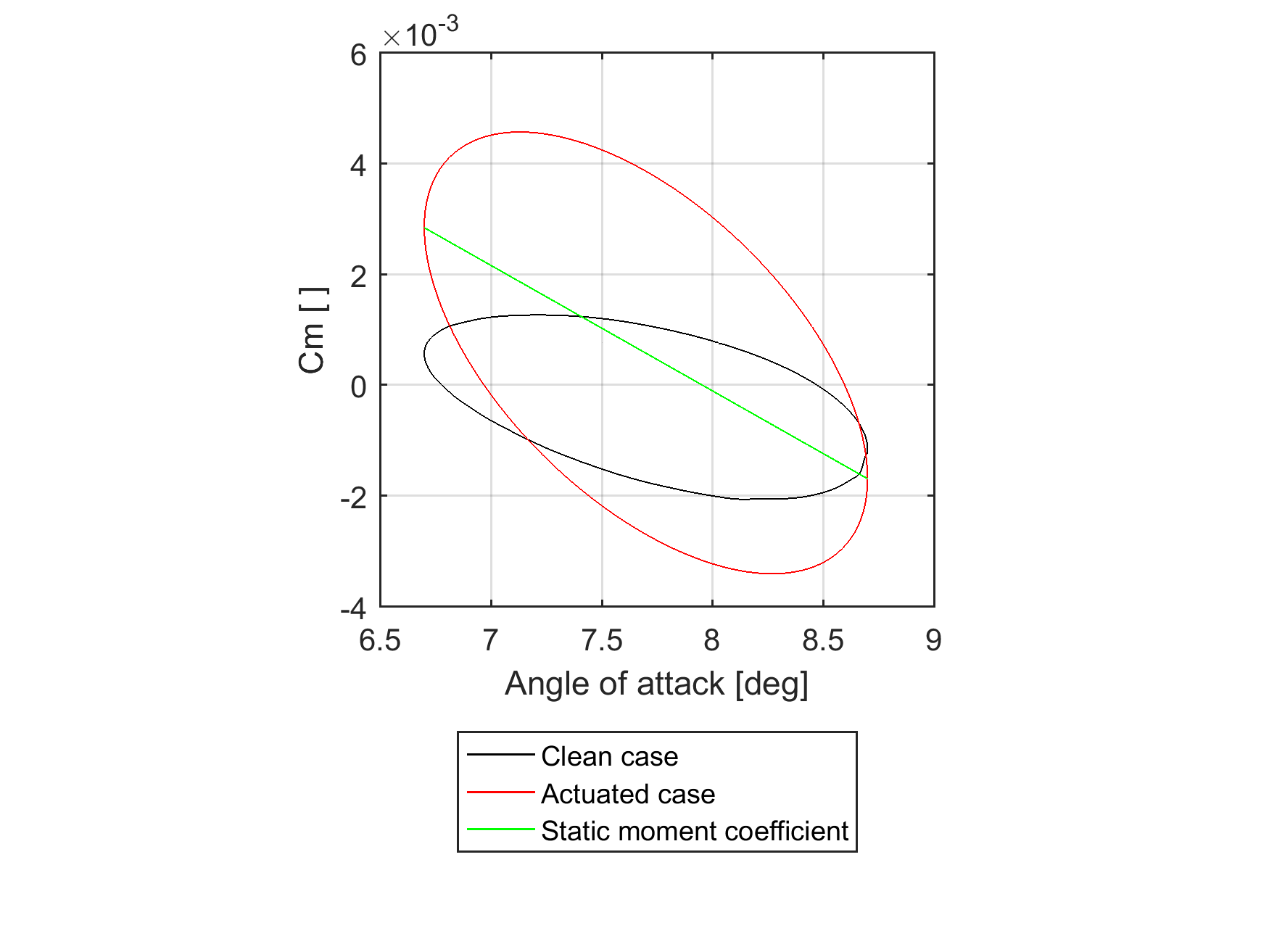

Supplement: Multimedia component 1 [file mmc1.zip › Allegati/w20_a1/Force_w20_a1_45/Moment Coefficient Hysteresis curve.png]

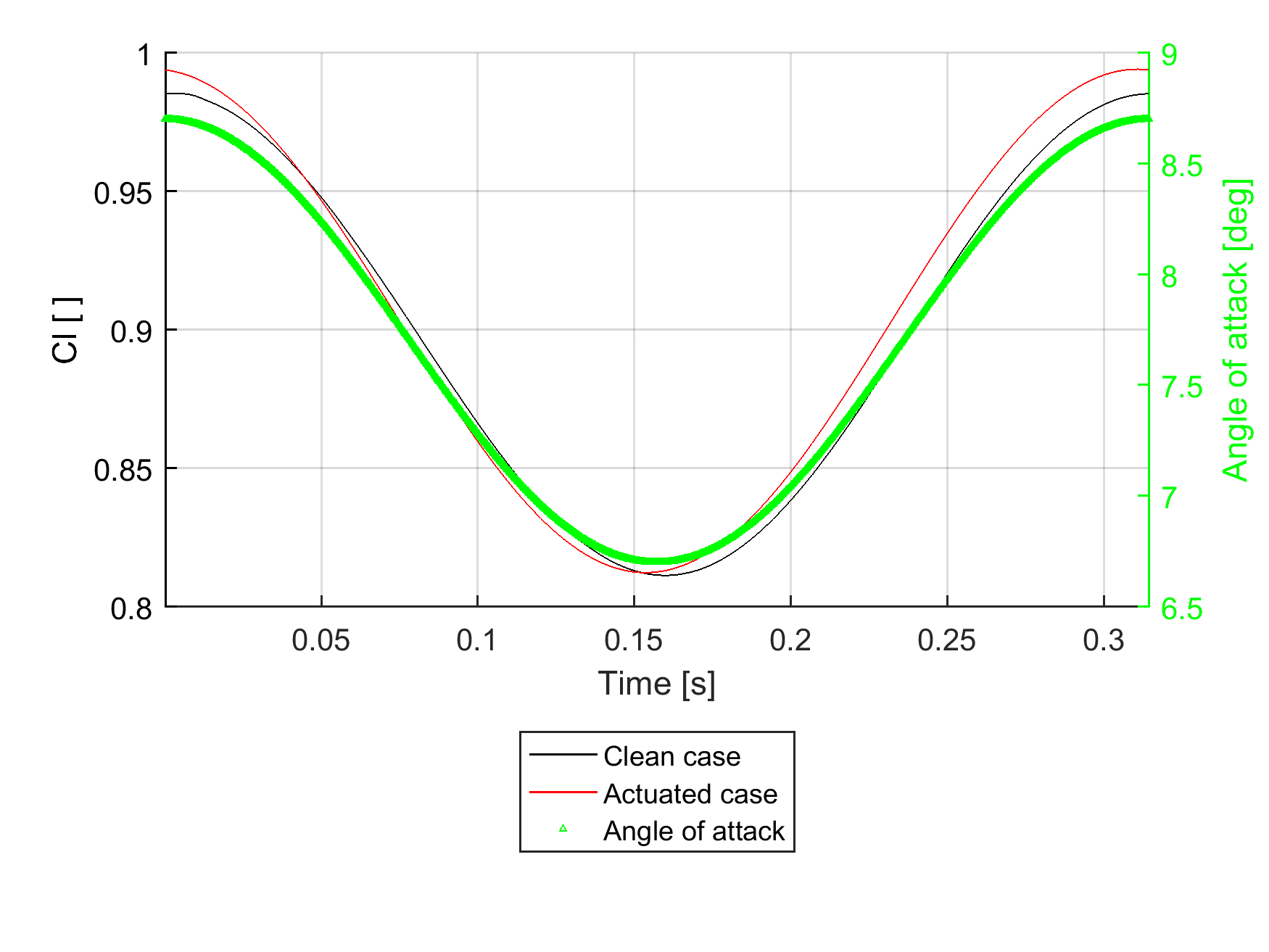

Supplement: Multimedia component 1 [file mmc1.zip › Allegati/w20_a1/Force_w20_a1_90/Lift Coefficient comparison.png]

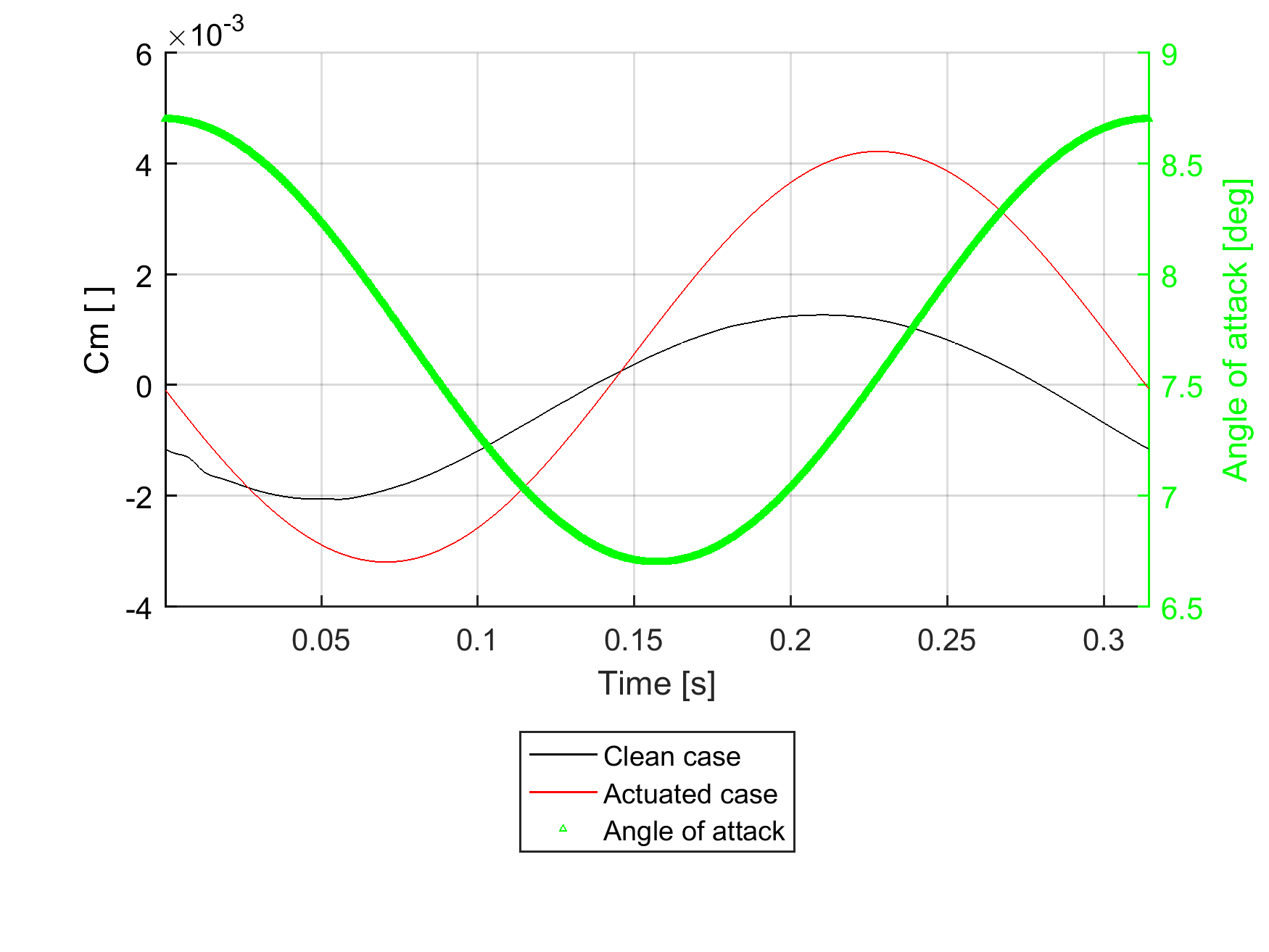

Supplement: Multimedia component 1 [file mmc1.zip › Allegati/w20_a1/Force_w20_a1_90/Moment Coefficient comparison.png]

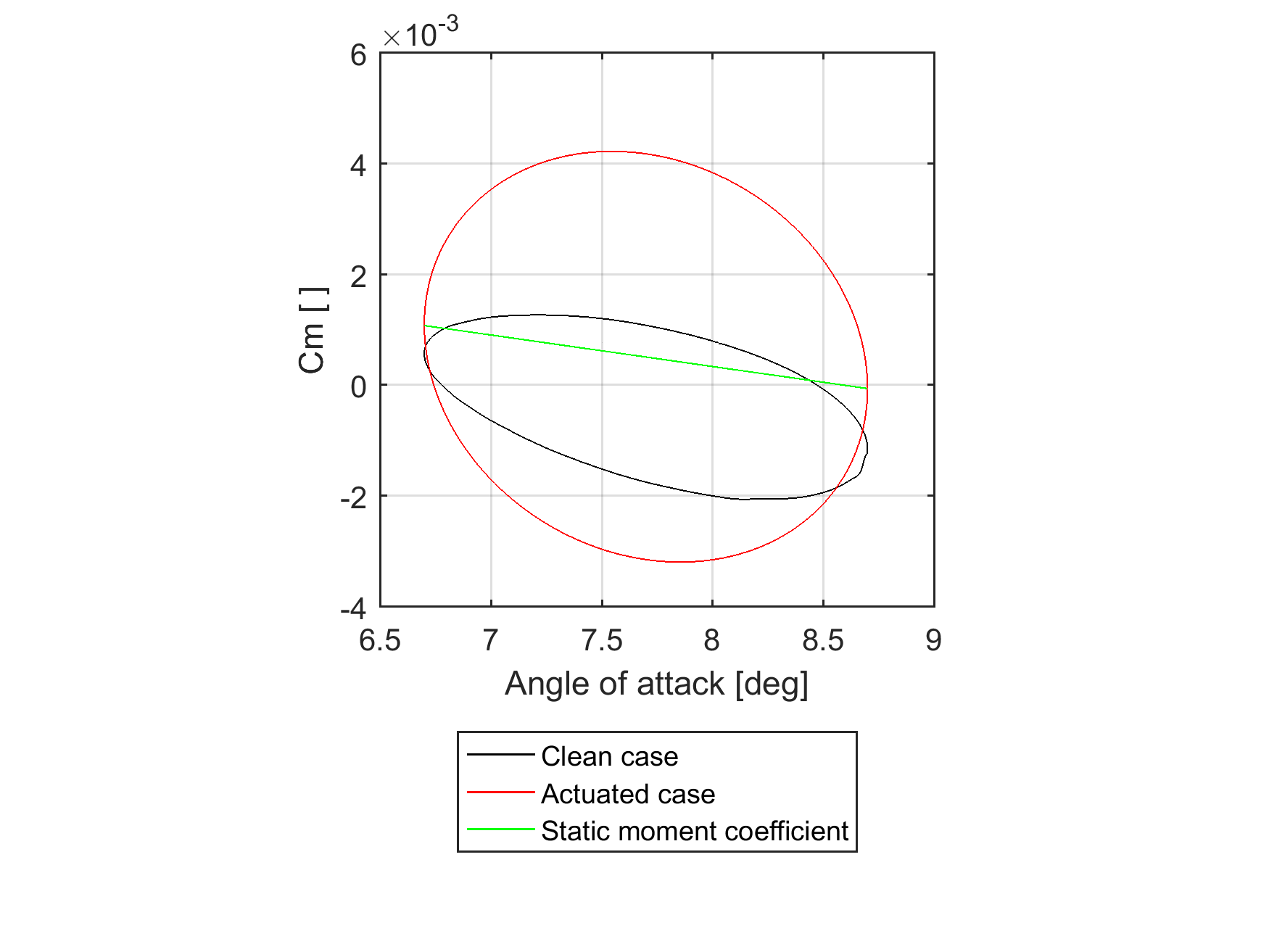

Supplement: Multimedia component 1 [file mmc1.zip › Allegati/w20_a1/Force_w20_a1_90/Moment Coefficient Hysteresis curve.png]

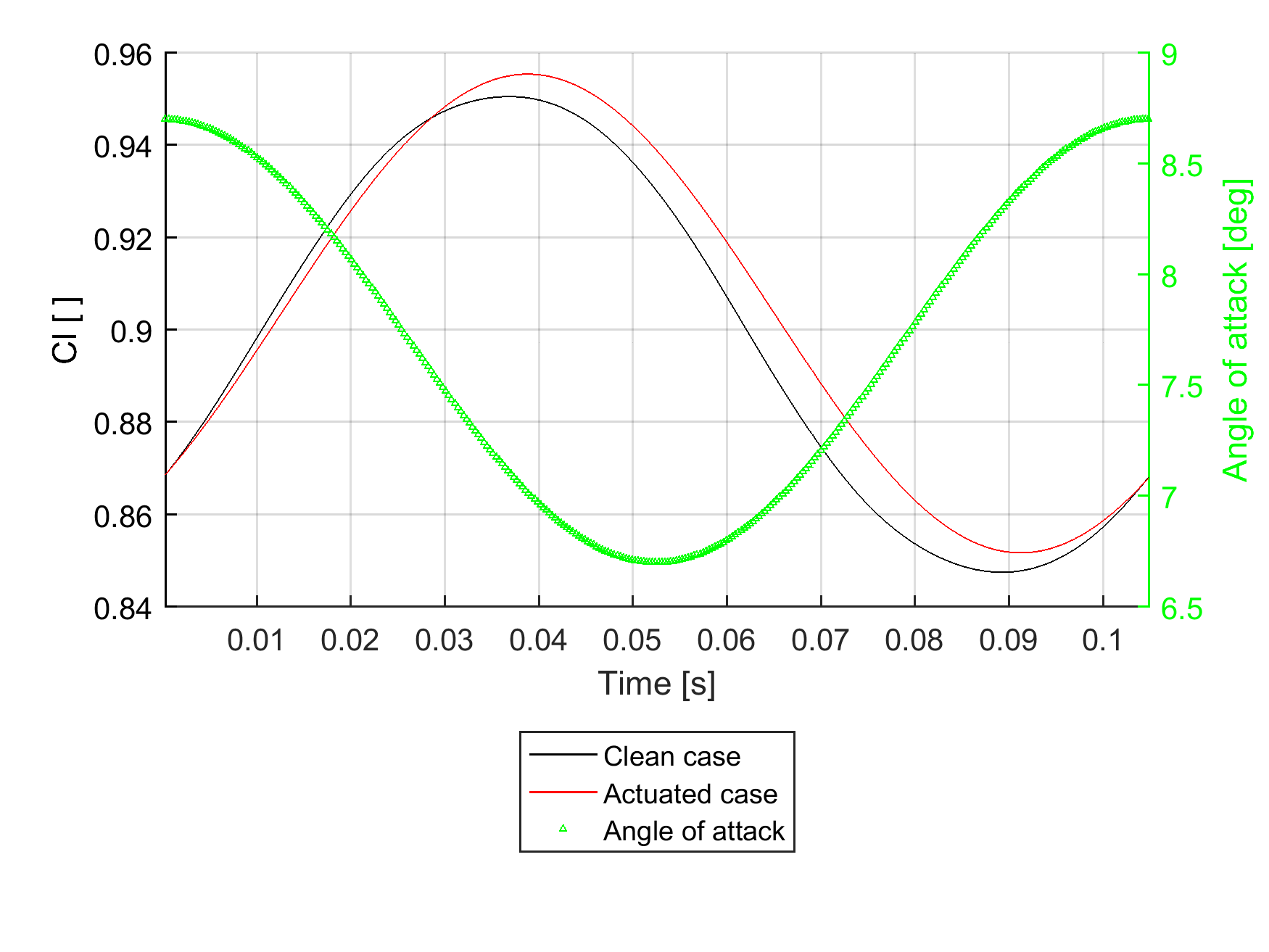

Supplement: Multimedia component 1 [file mmc1.zip › Allegati/w60_a1/Force_w60_a1_0/Lift Coefficient comparison.png]

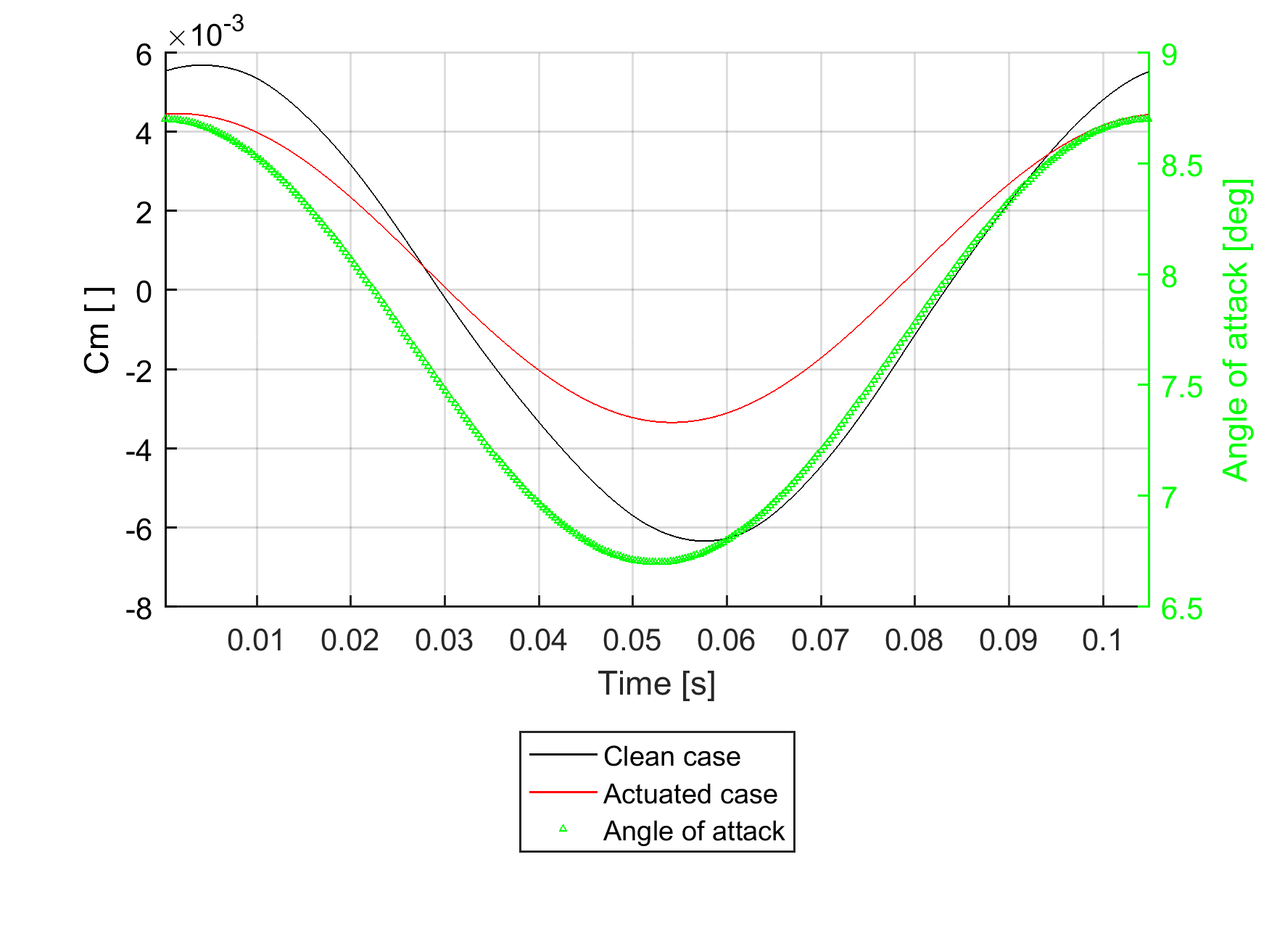

Supplement: Multimedia component 1 [file mmc1.zip › Allegati/w60_a1/Force_w60_a1_0/Moment Coefficient comparison.png]

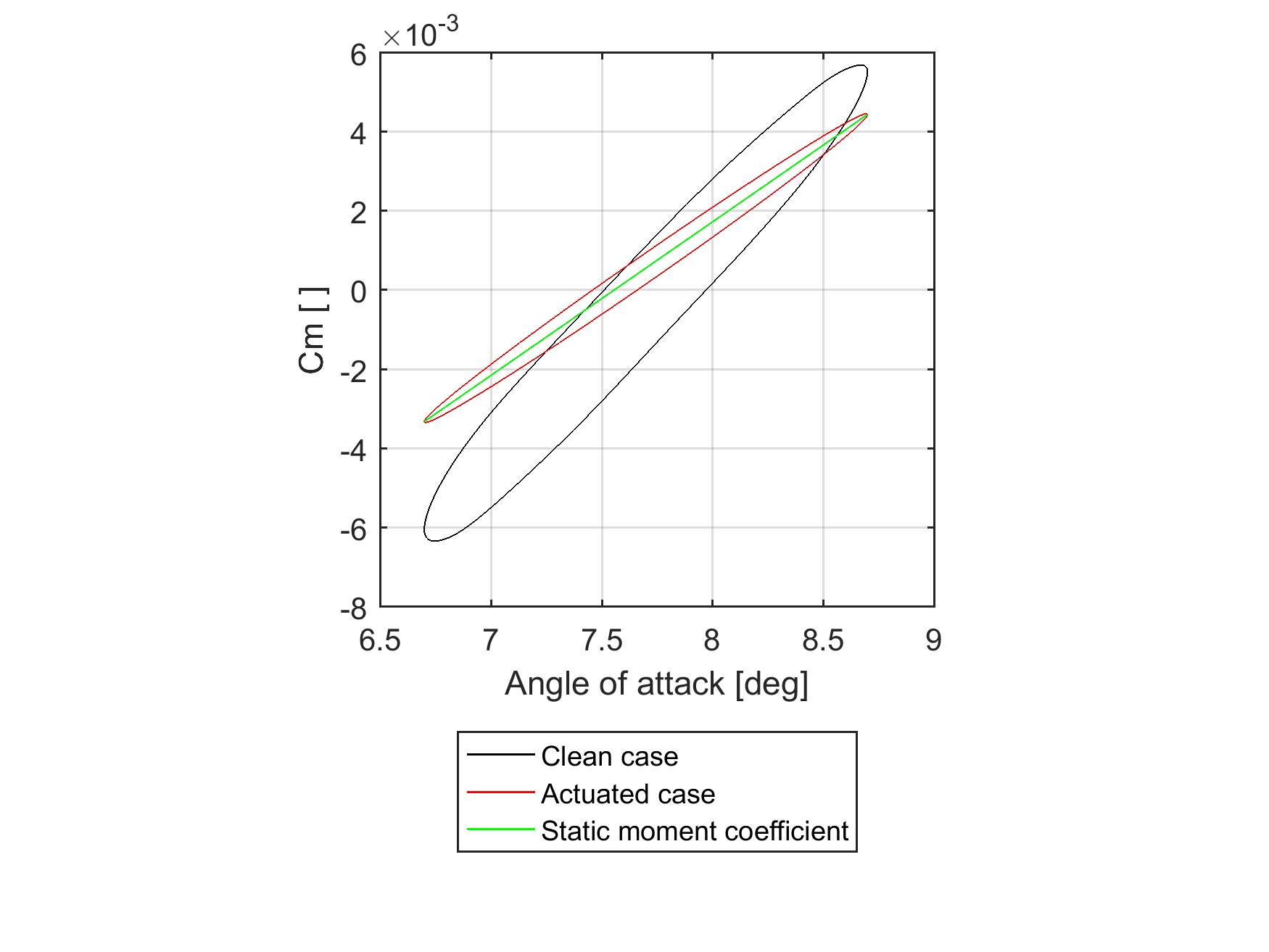

Supplement: Multimedia component 1 [file mmc1.zip › Allegati/w60_a1/Force_w60_a1_0/Moment Coefficient Hysteresis curve.png]

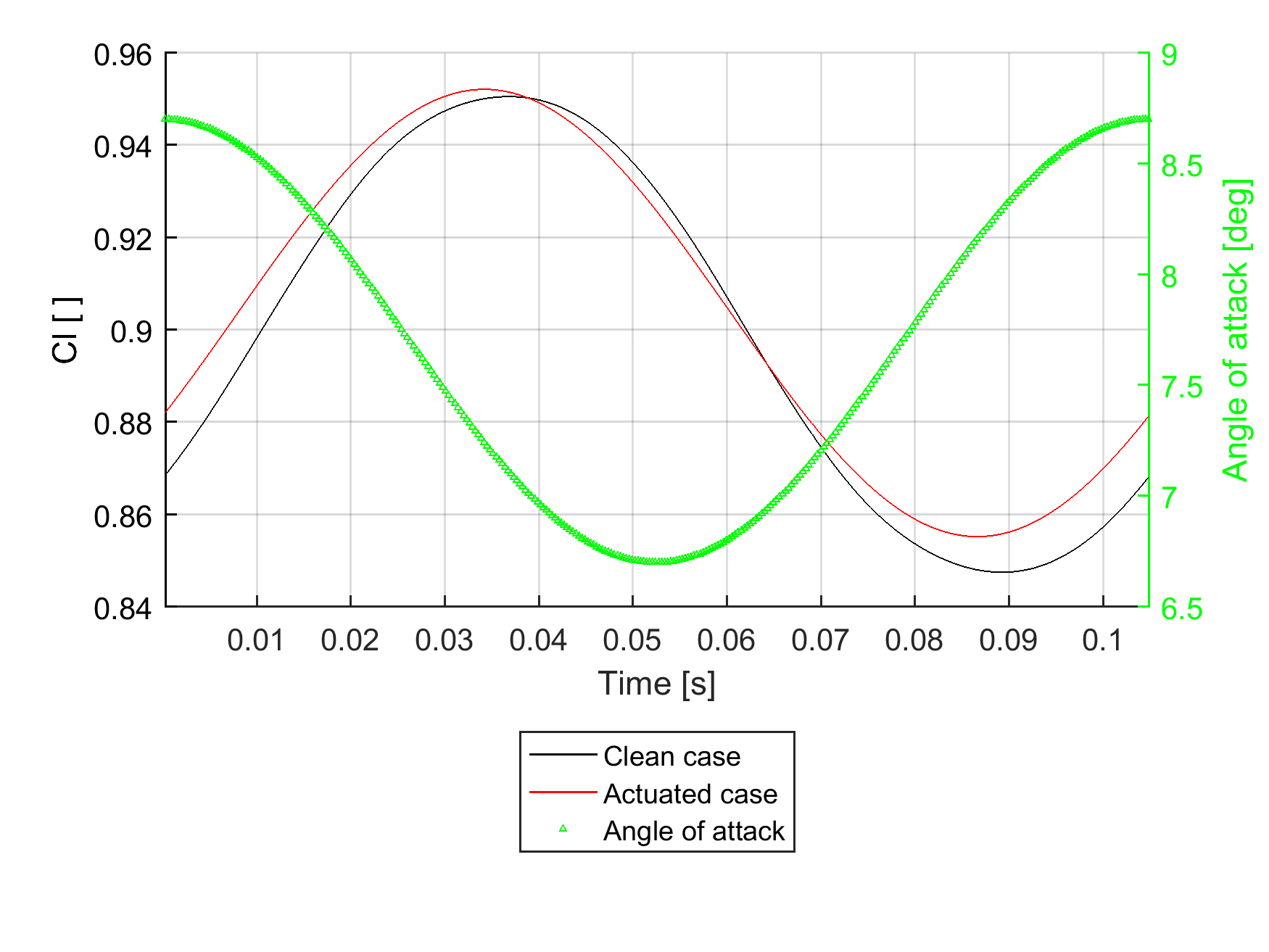

Supplement: Multimedia component 1 [file mmc1.zip › Allegati/w60_a1/Force_w60_a1_135/Lift Coefficient comparison.png]

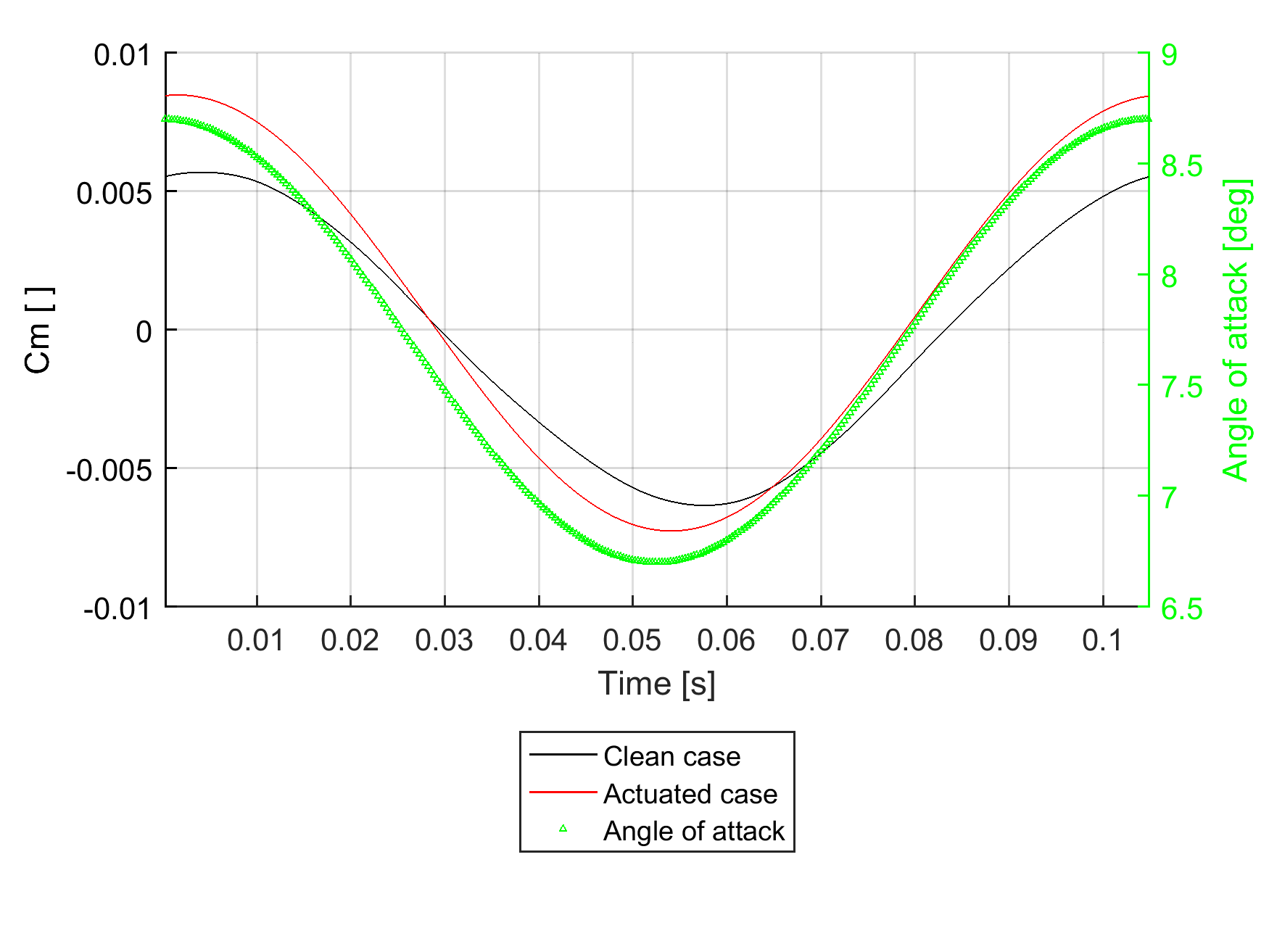

Supplement: Multimedia component 1 [file mmc1.zip › Allegati/w60_a1/Force_w60_a1_135/Moment Coefficient comparison.png]

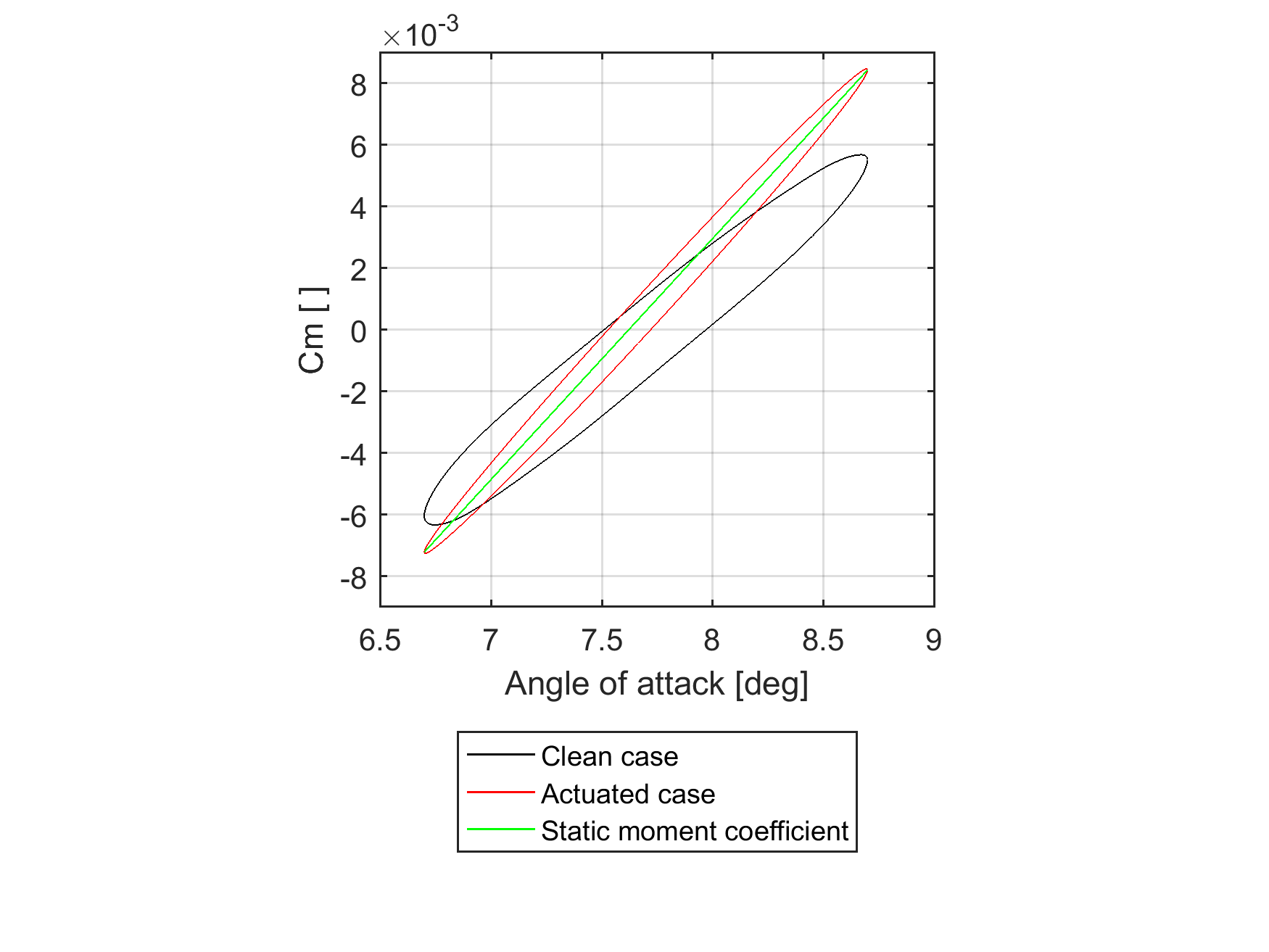

Supplement: Multimedia component 1 [file mmc1.zip › Allegati/w60_a1/Force_w60_a1_135/Moment Coefficient Hysteresis curve.png]

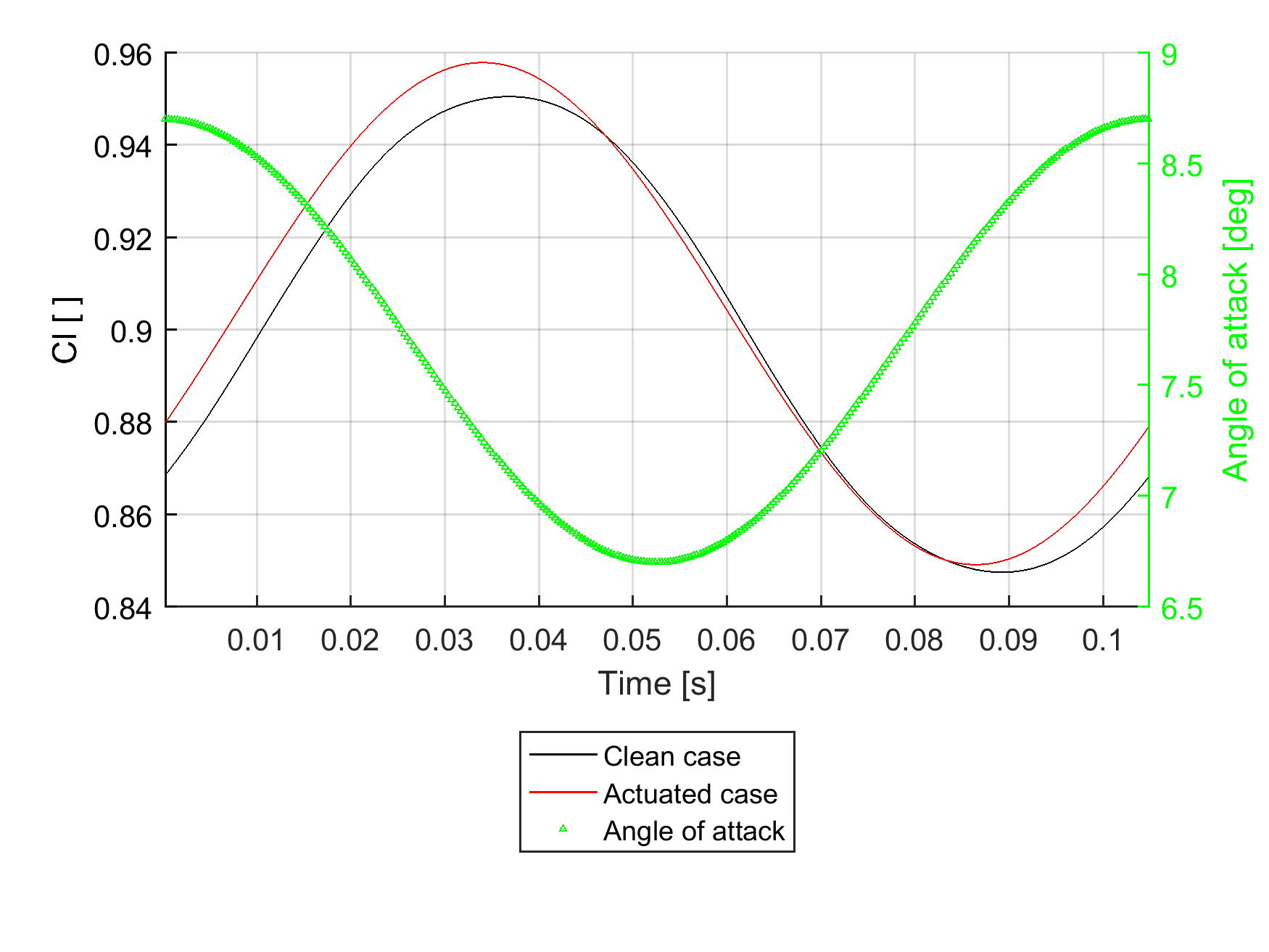

Supplement: Multimedia component 1 [file mmc1.zip › Allegati/w60_a1/Force_w60_a1_180/Lift Coefficient comparison.png]

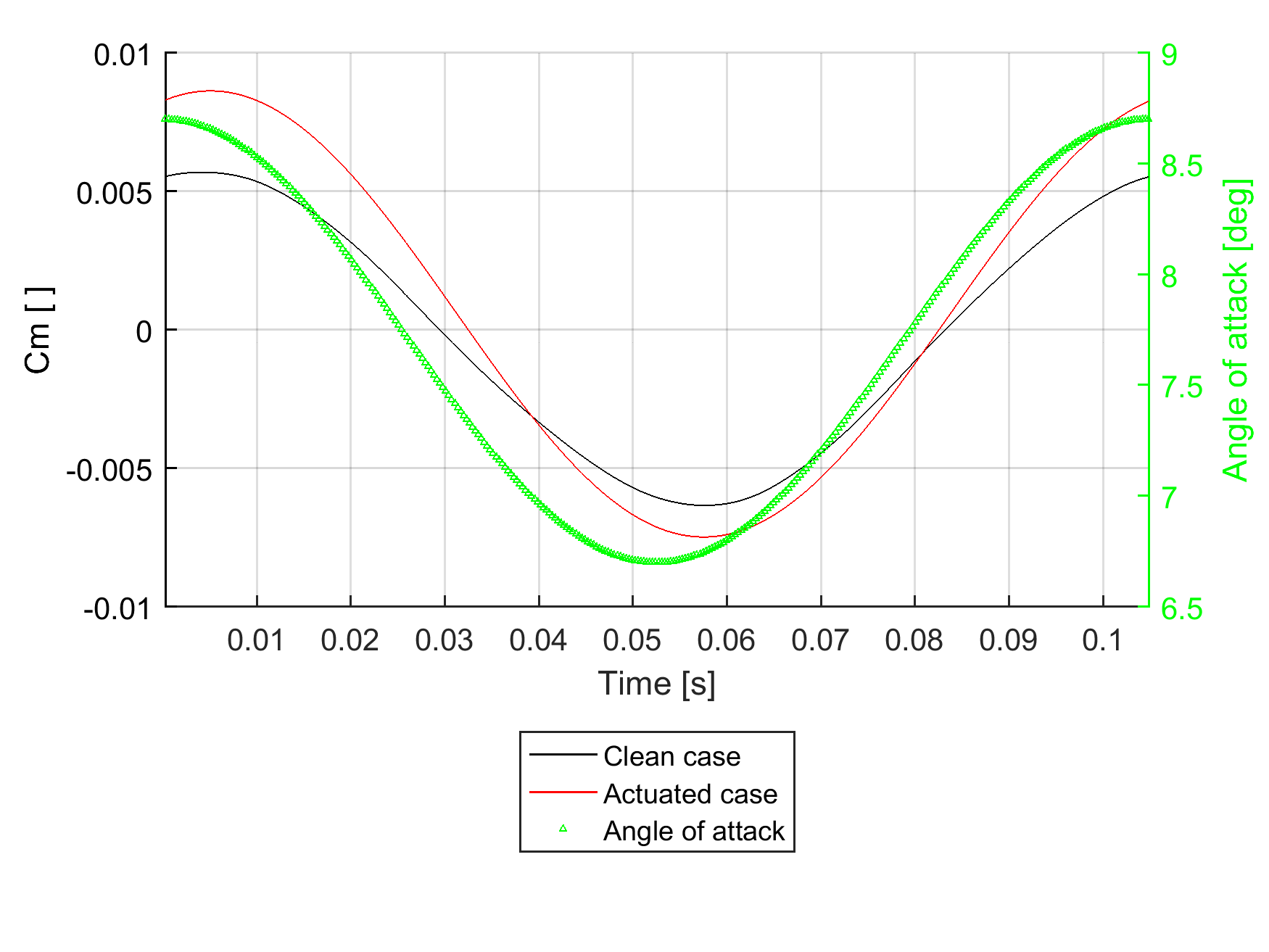

Supplement: Multimedia component 1 [file mmc1.zip › Allegati/w60_a1/Force_w60_a1_180/Moment Coefficient comparison.png]

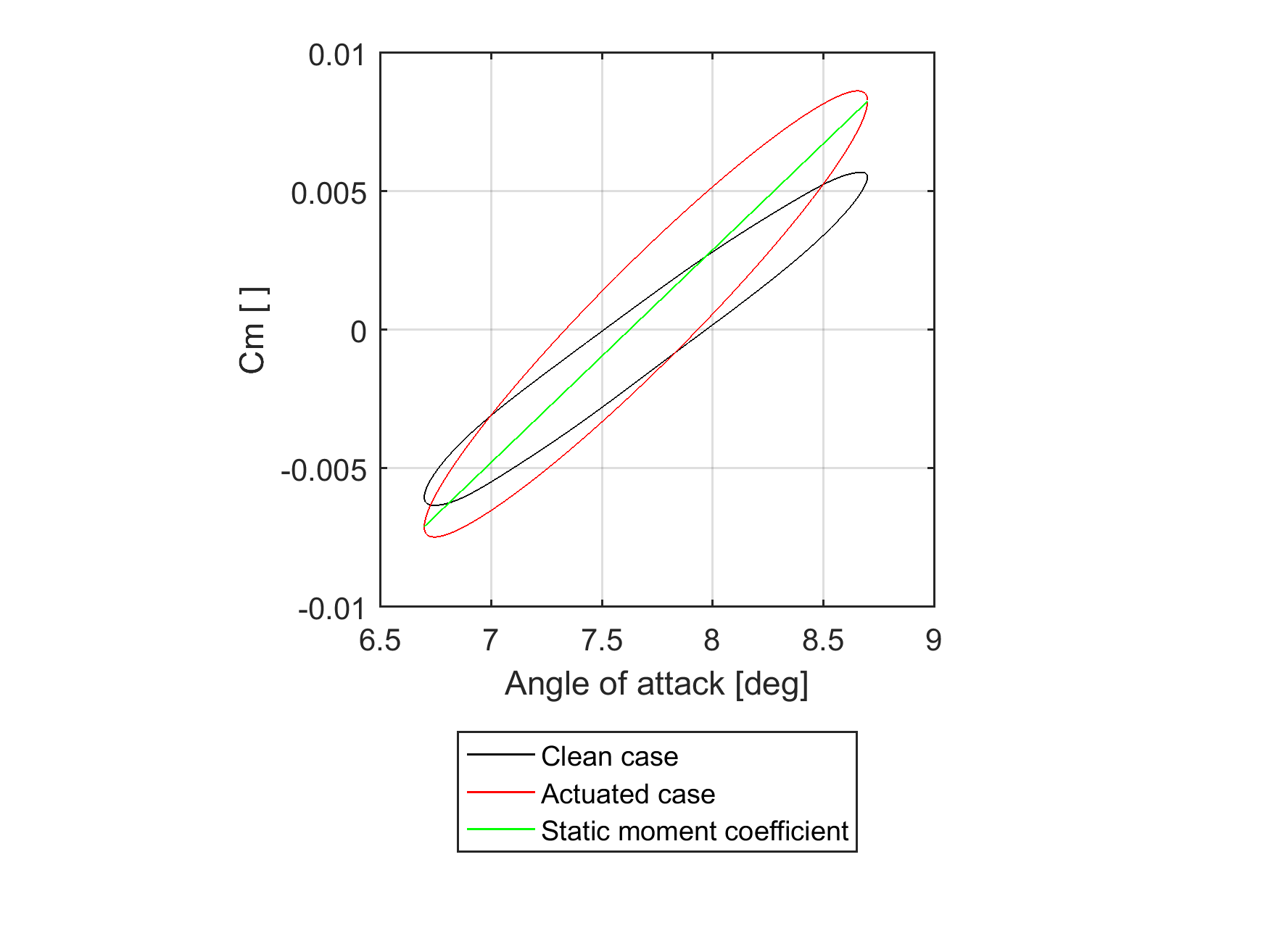

Supplement: Multimedia component 1 [file mmc1.zip › Allegati/w60_a1/Force_w60_a1_180/Moment Coefficient Hysteresis curve.png]

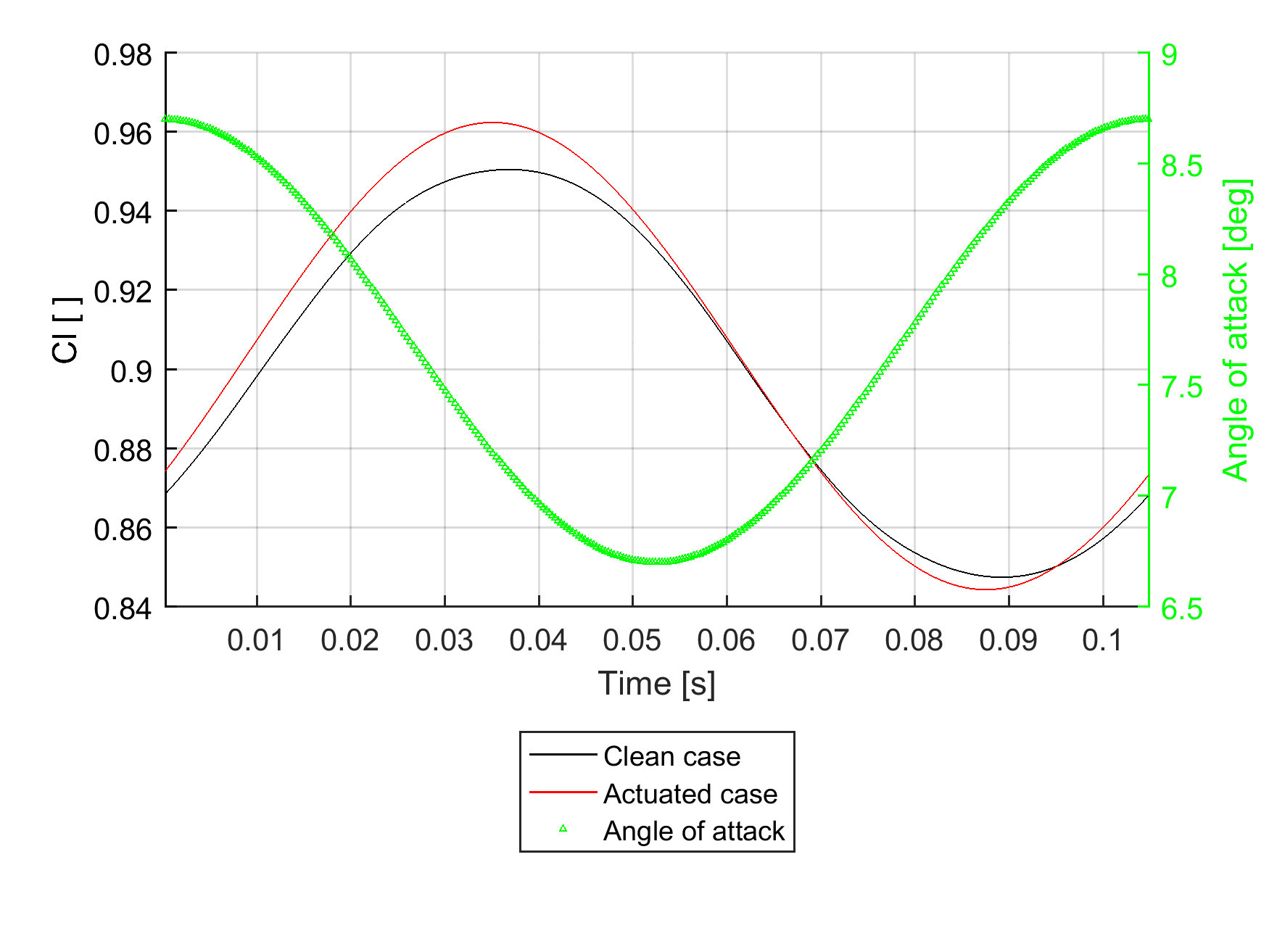

Supplement: Multimedia component 1 [file mmc1.zip › Allegati/w60_a1/Force_w60_a1_225/Lift Coefficient comparison.png]

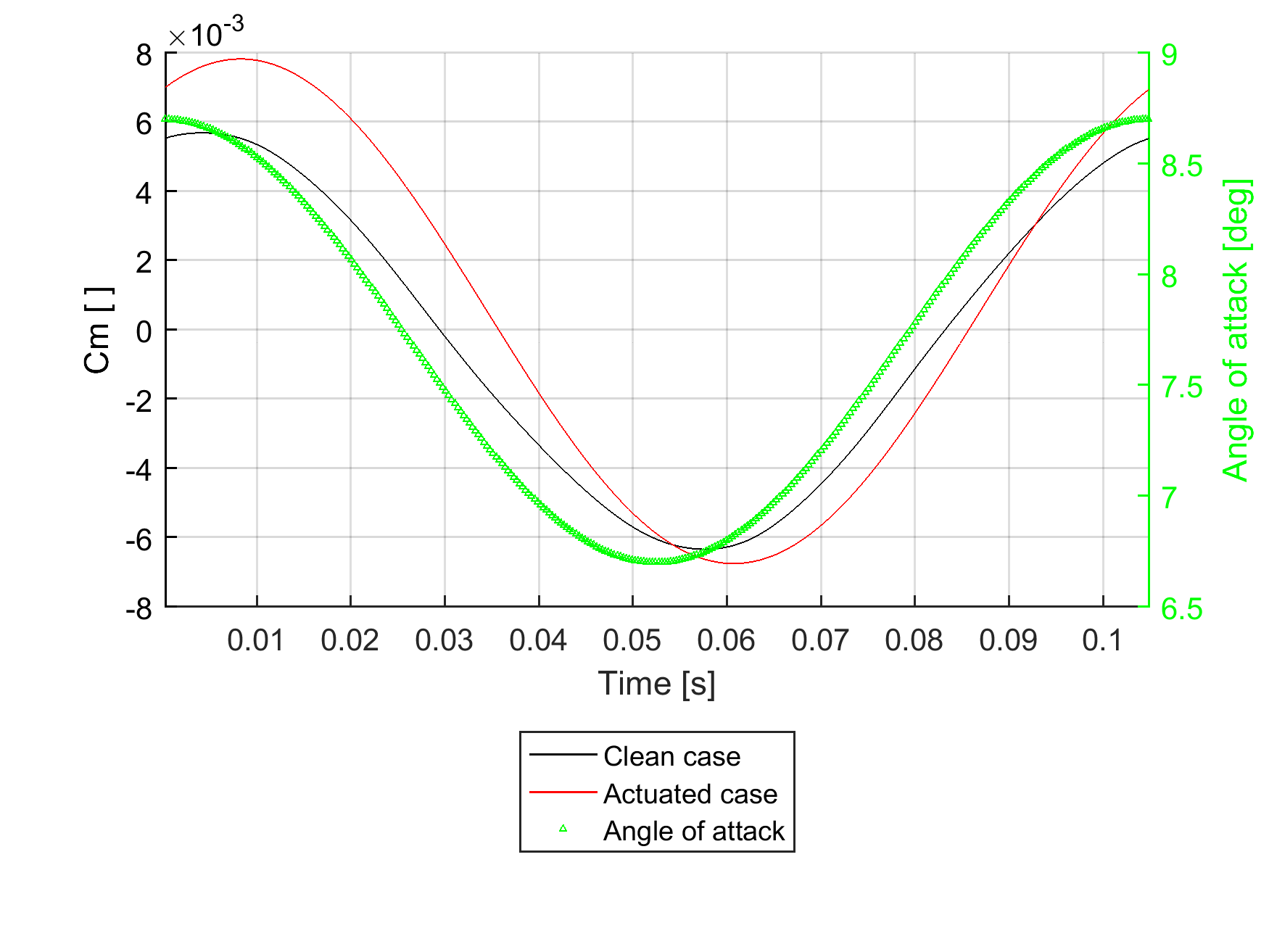

Supplement: Multimedia component 1 [file mmc1.zip › Allegati/w60_a1/Force_w60_a1_225/Moment Coefficient comparison.png]

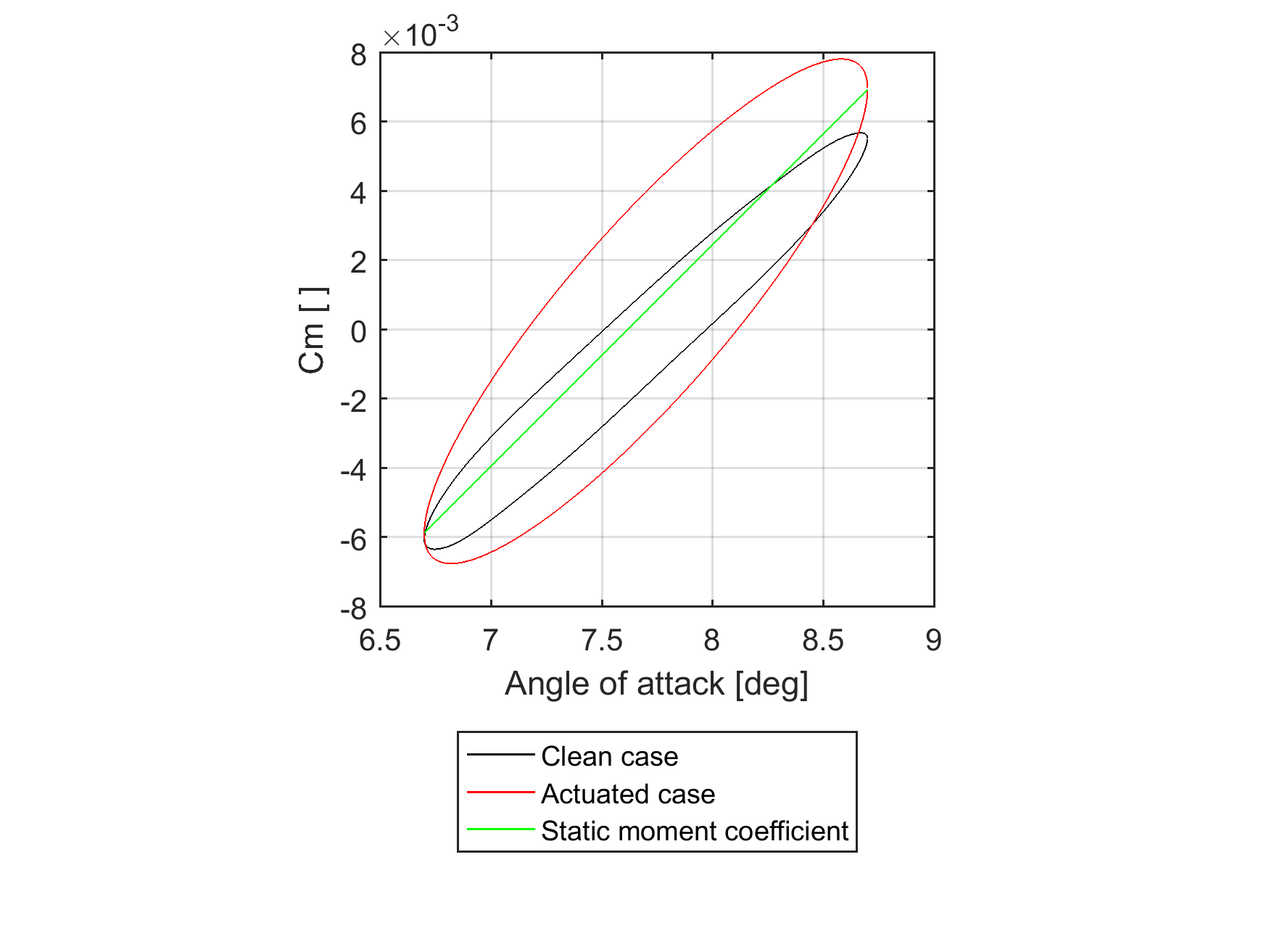

Supplement: Multimedia component 1 [file mmc1.zip › Allegati/w60_a1/Force_w60_a1_225/Moment Coefficient Hysteresis curve.png]

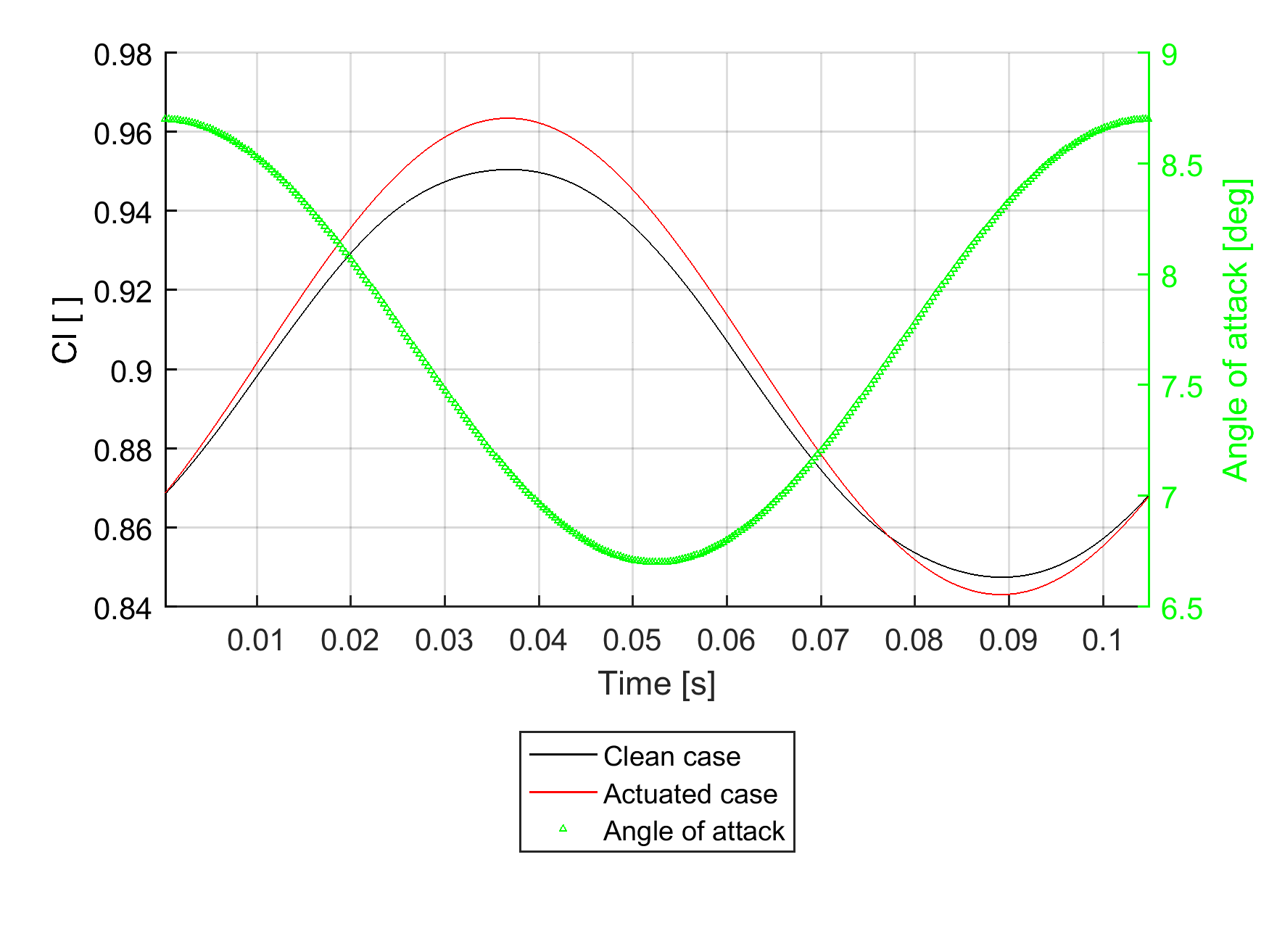

Supplement: Multimedia component 1 [file mmc1.zip › Allegati/w60_a1/Force_w60_a1_270/Lift Coefficient comparison.png]

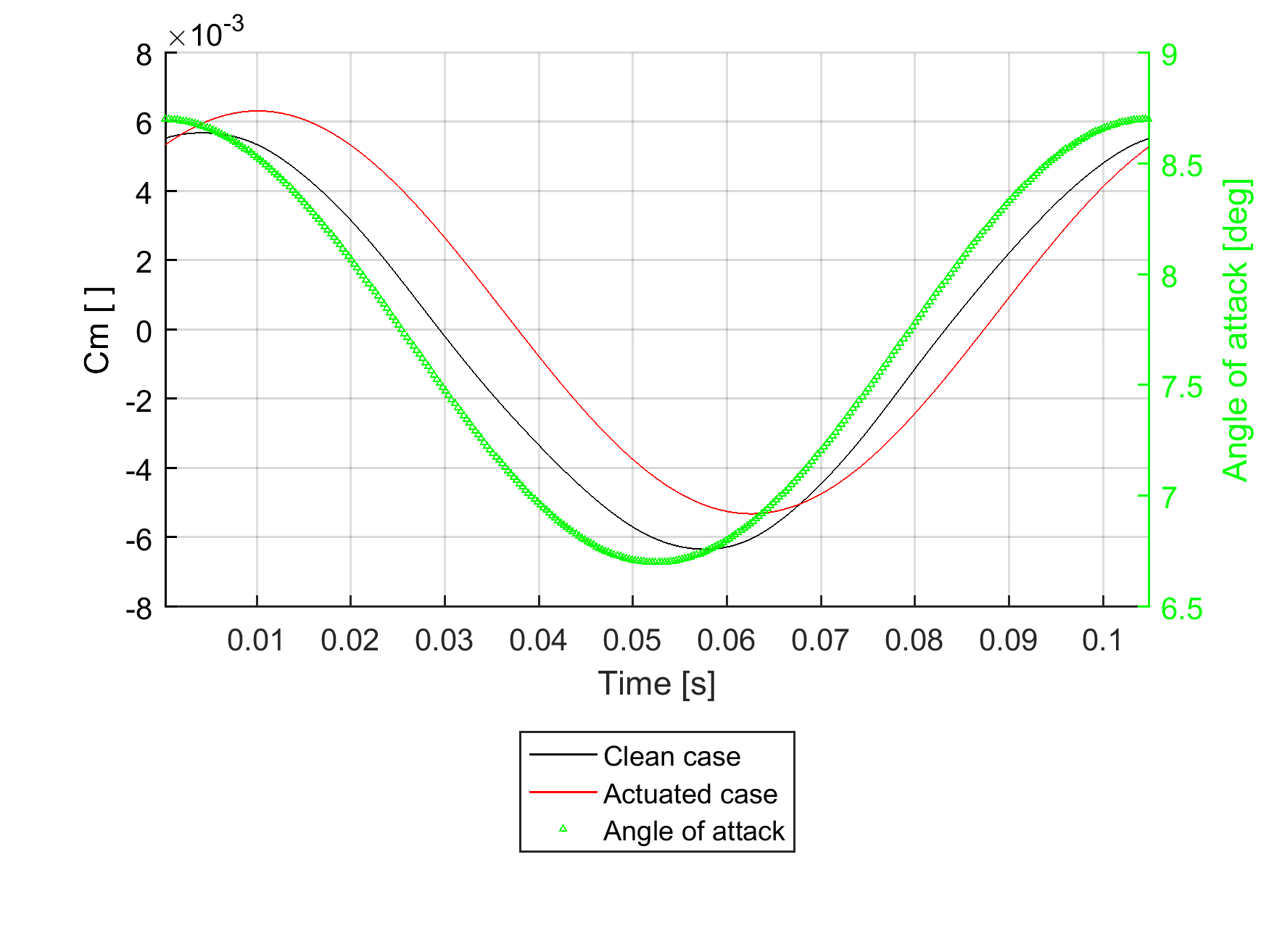

Supplement: Multimedia component 1 [file mmc1.zip › Allegati/w60_a1/Force_w60_a1_270/Moment Coefficient comparison.png]

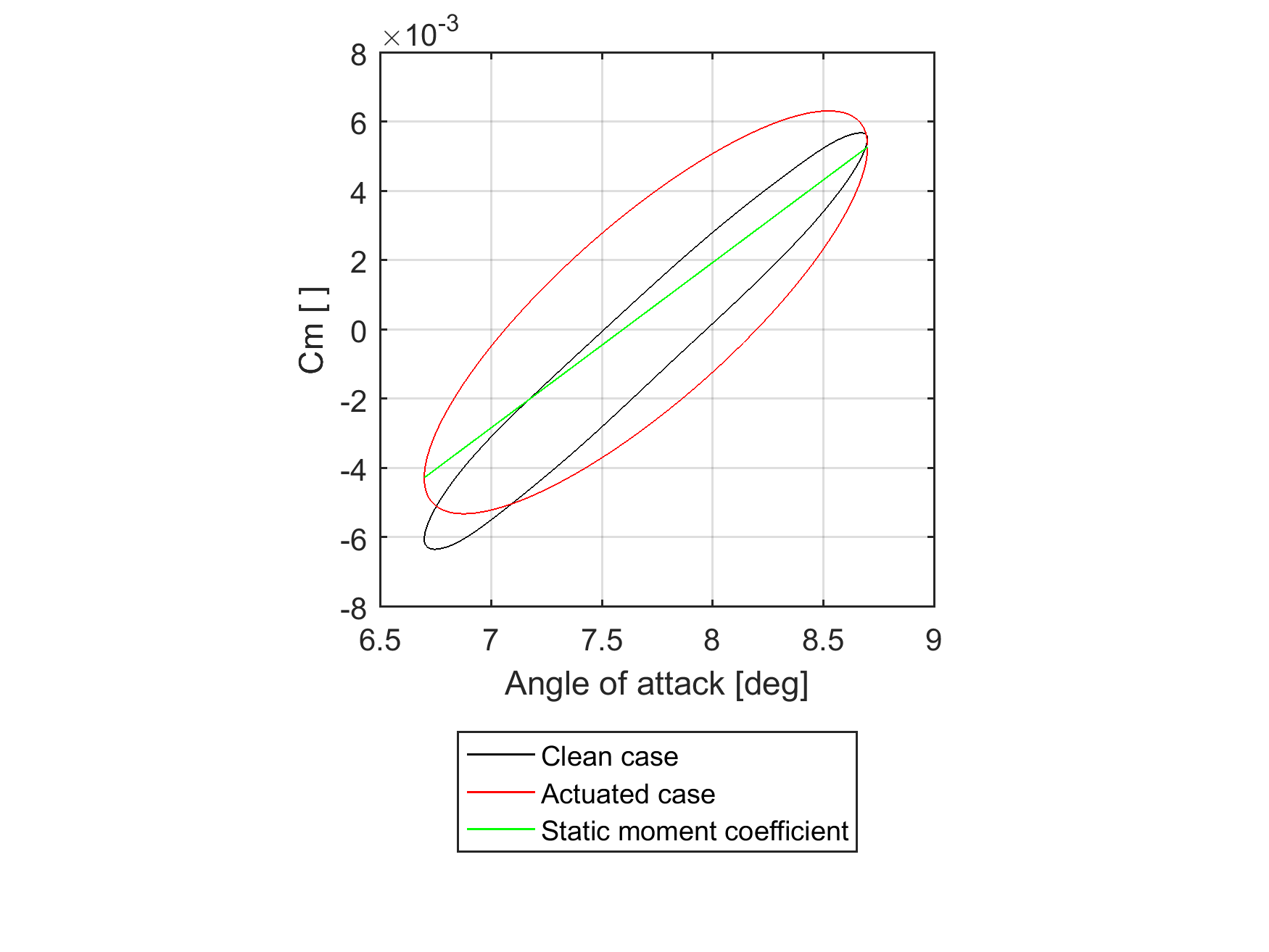

Supplement: Multimedia component 1 [file mmc1.zip › Allegati/w60_a1/Force_w60_a1_270/Moment Coefficient Hysteresis curve.png]

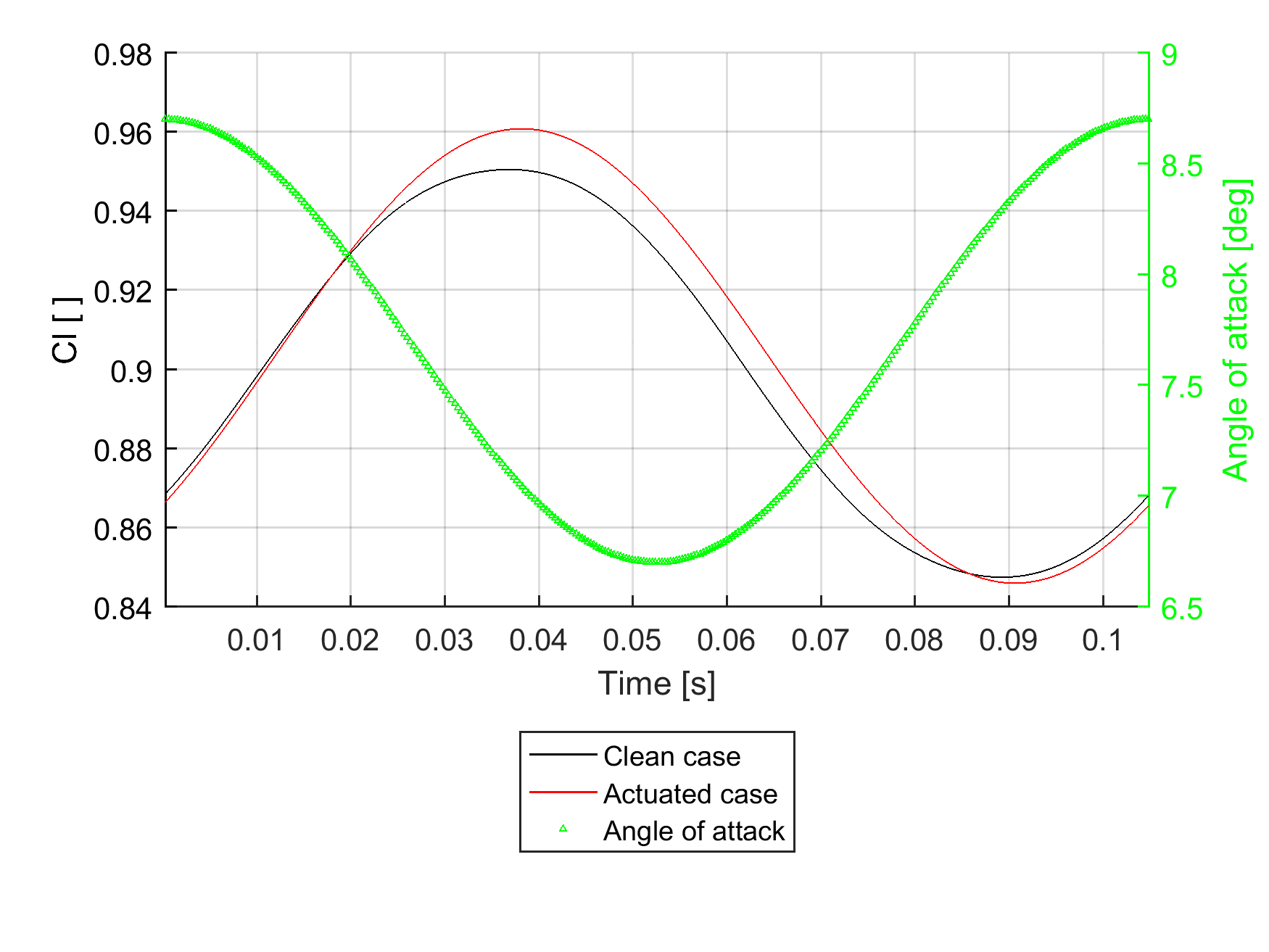

Supplement: Multimedia component 1 [file mmc1.zip › Allegati/w60_a1/Force_w60_a1_315/Lift Coefficient comparison.png]

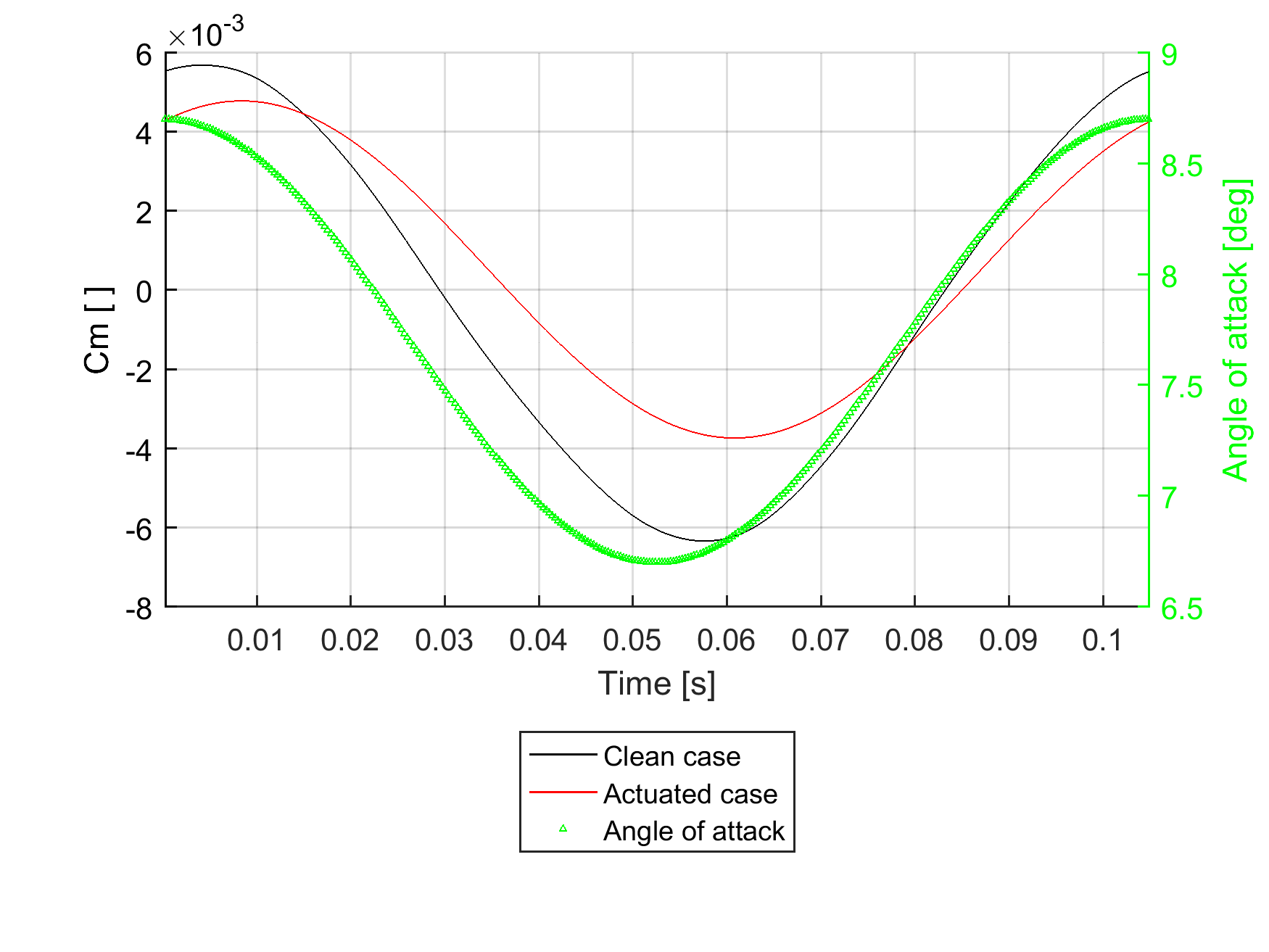

Supplement: Multimedia component 1 [file mmc1.zip › Allegati/w60_a1/Force_w60_a1_315/Moment Coefficient comparison.png]

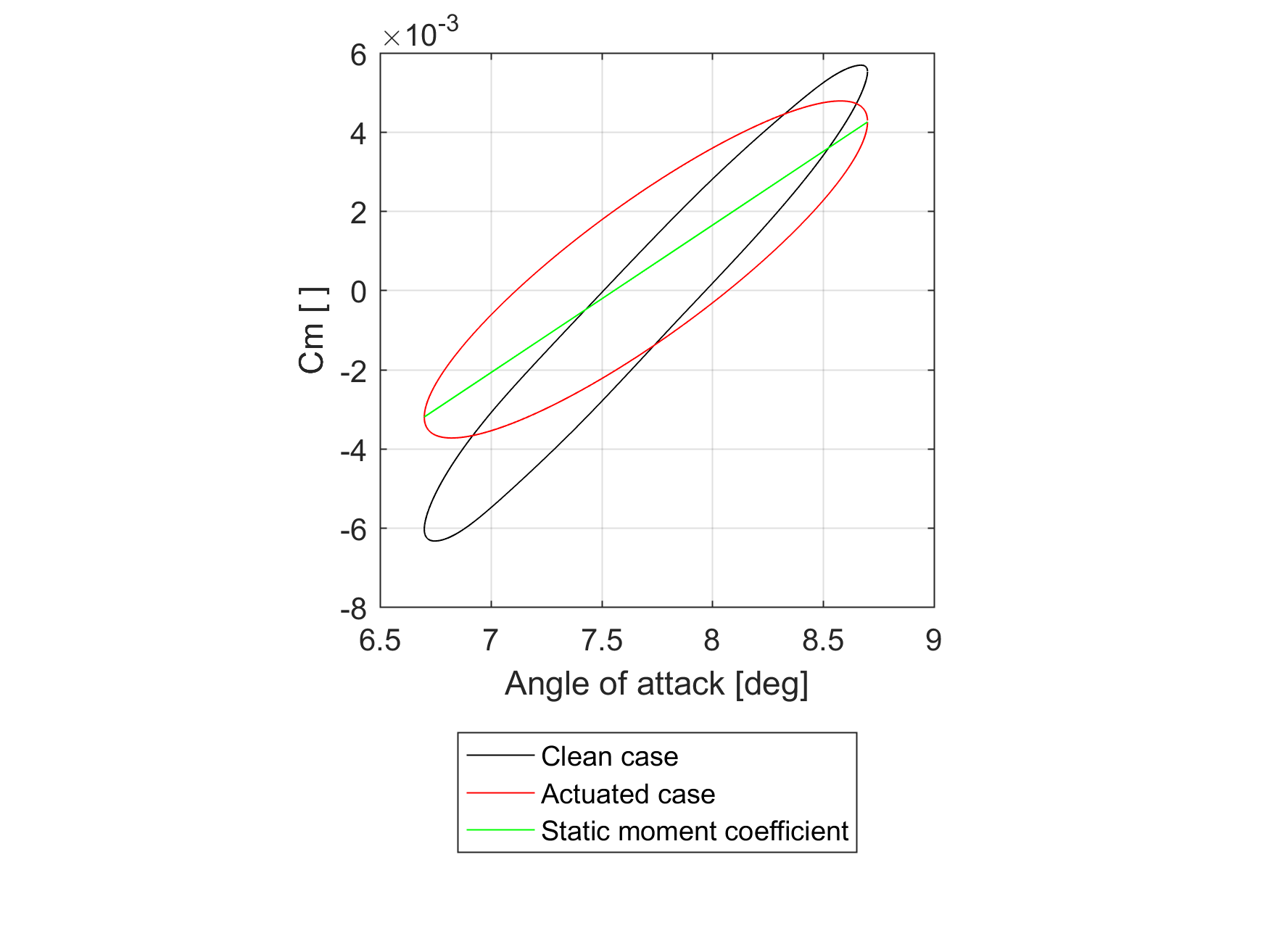

Supplement: Multimedia component 1 [file mmc1.zip › Allegati/w60_a1/Force_w60_a1_315/Moment Coefficient Hysteresis curve.png]

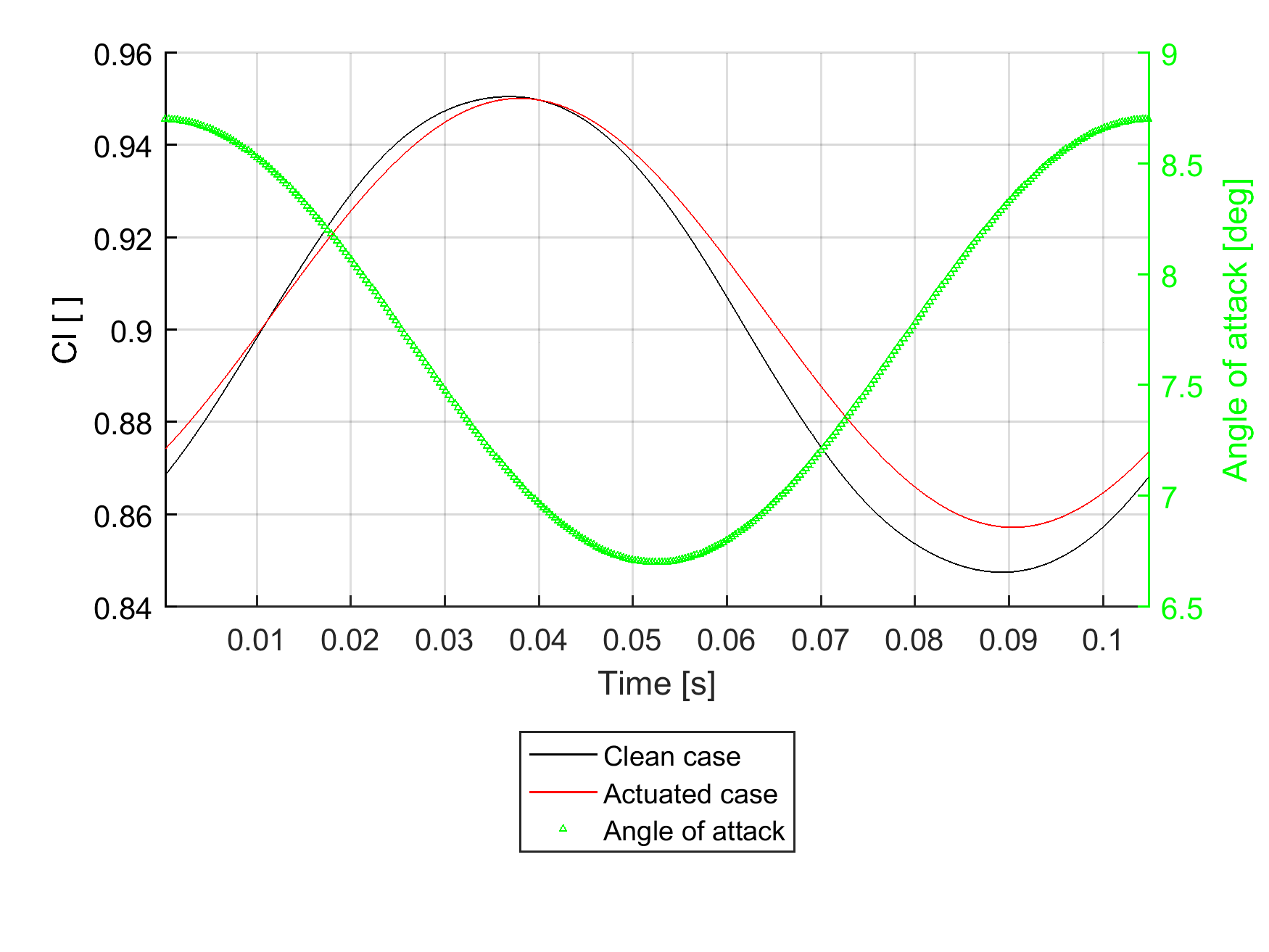

Supplement: Multimedia component 1 [file mmc1.zip › Allegati/w60_a1/Force_w60_a1_45/Lift Coefficient comparison.png]

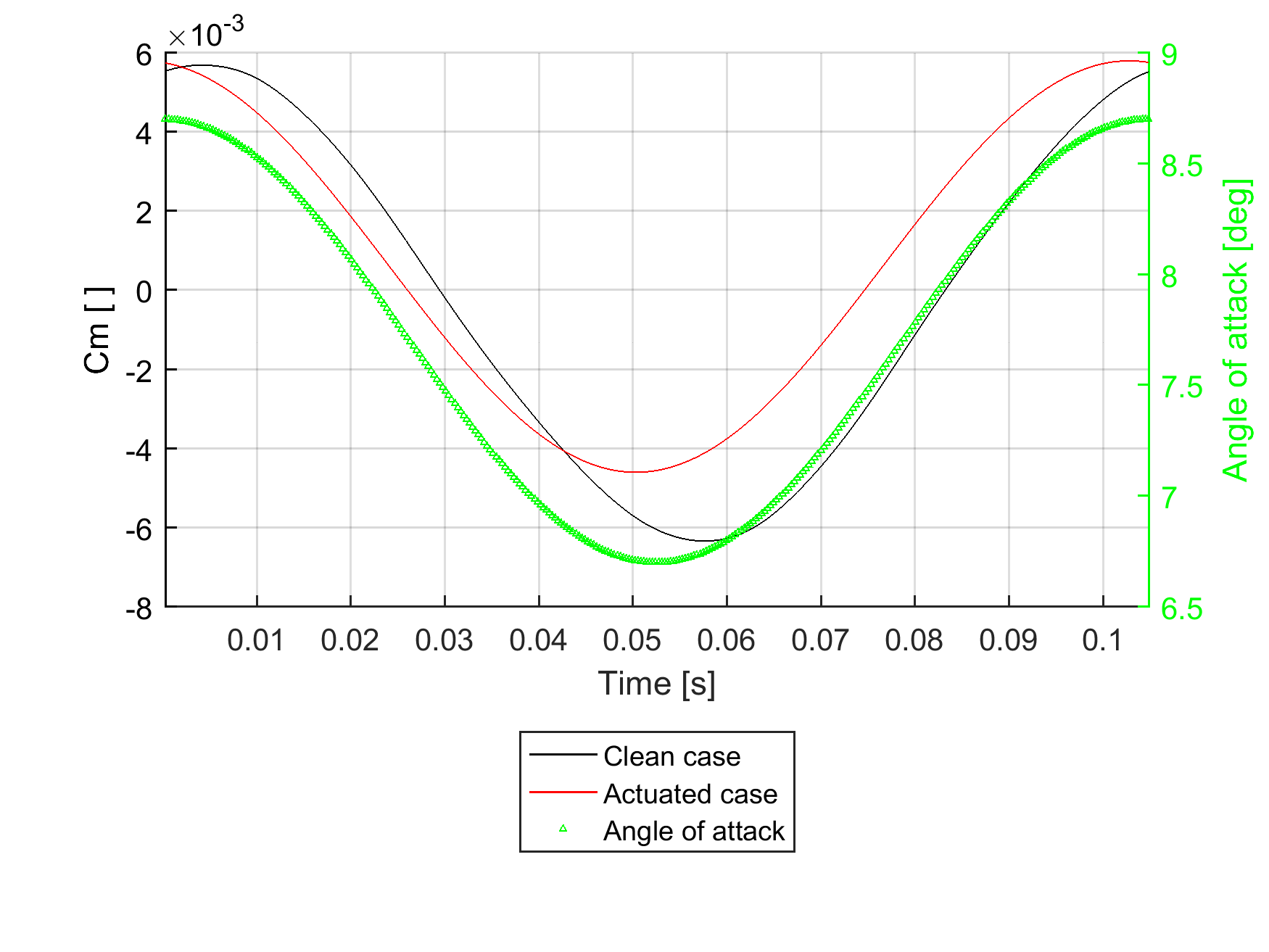

Supplement: Multimedia component 1 [file mmc1.zip › Allegati/w60_a1/Force_w60_a1_45/Moment Coefficient comparison.png]

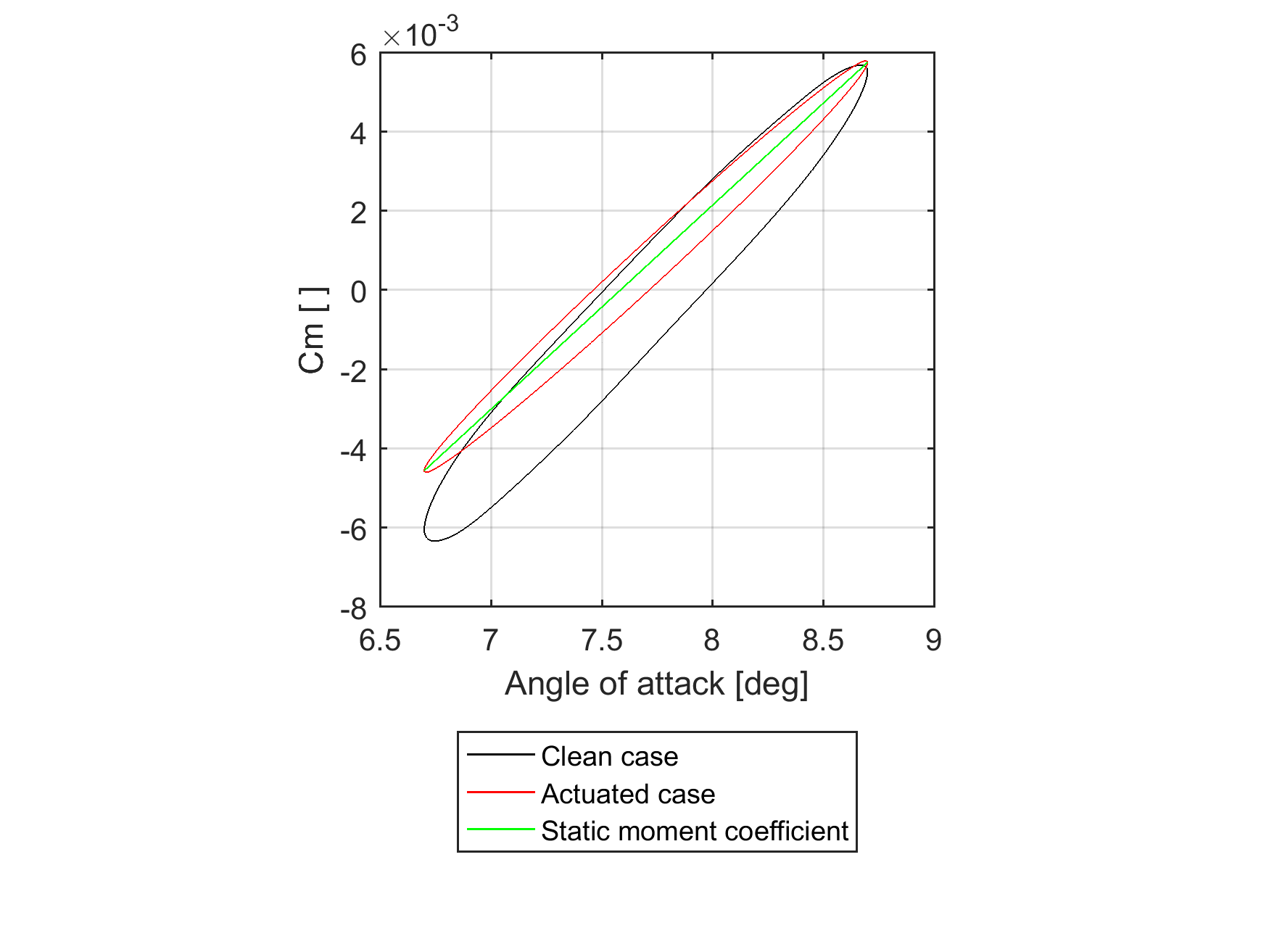

Supplement: Multimedia component 1 [file mmc1.zip › Allegati/w60_a1/Force_w60_a1_45/Moment Coefficient Hysteresis curve.png]

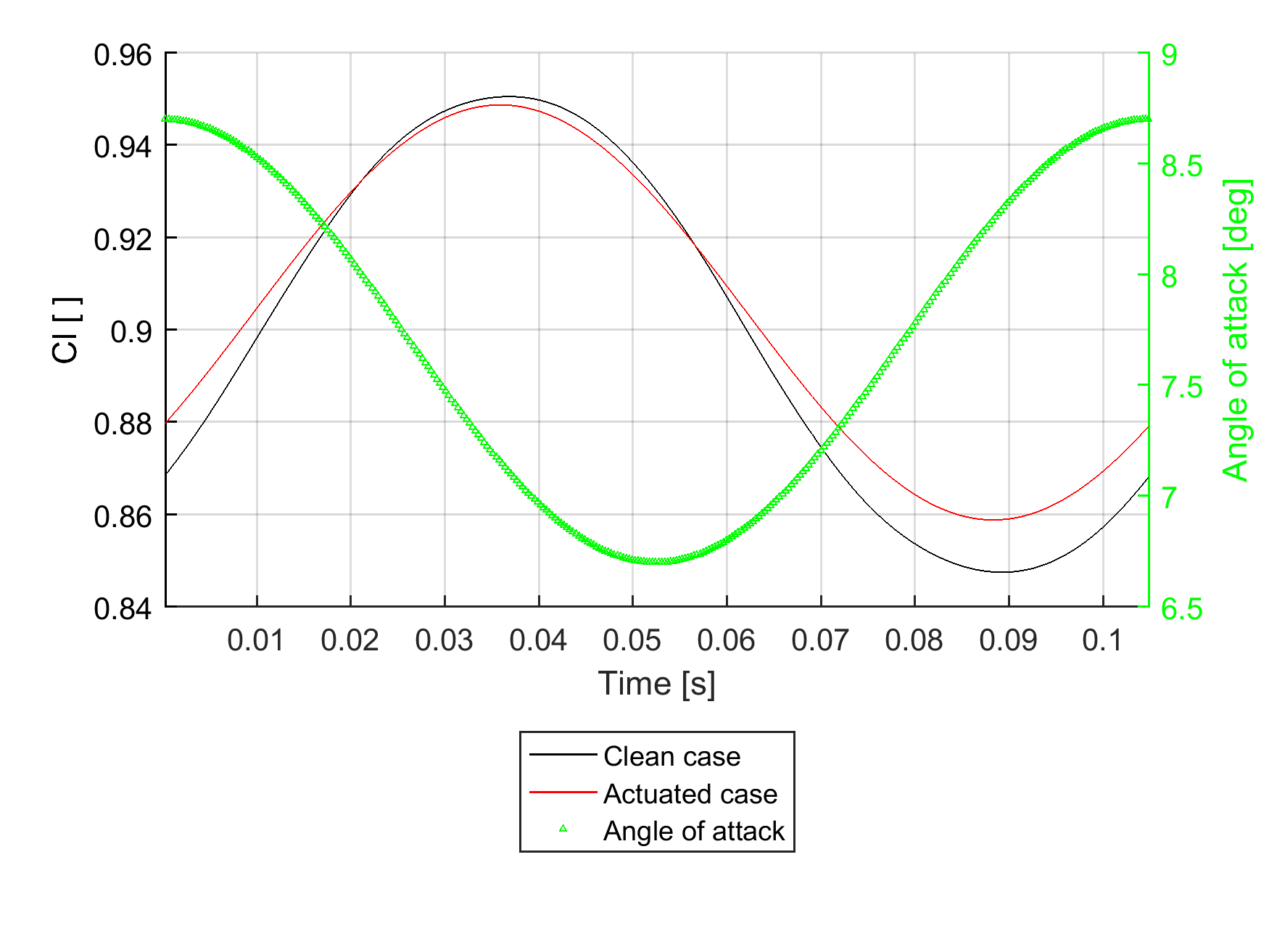

Supplement: Multimedia component 1 [file mmc1.zip › Allegati/w60_a1/Force_w60_a1_90/Lift Coefficient comparison.png]

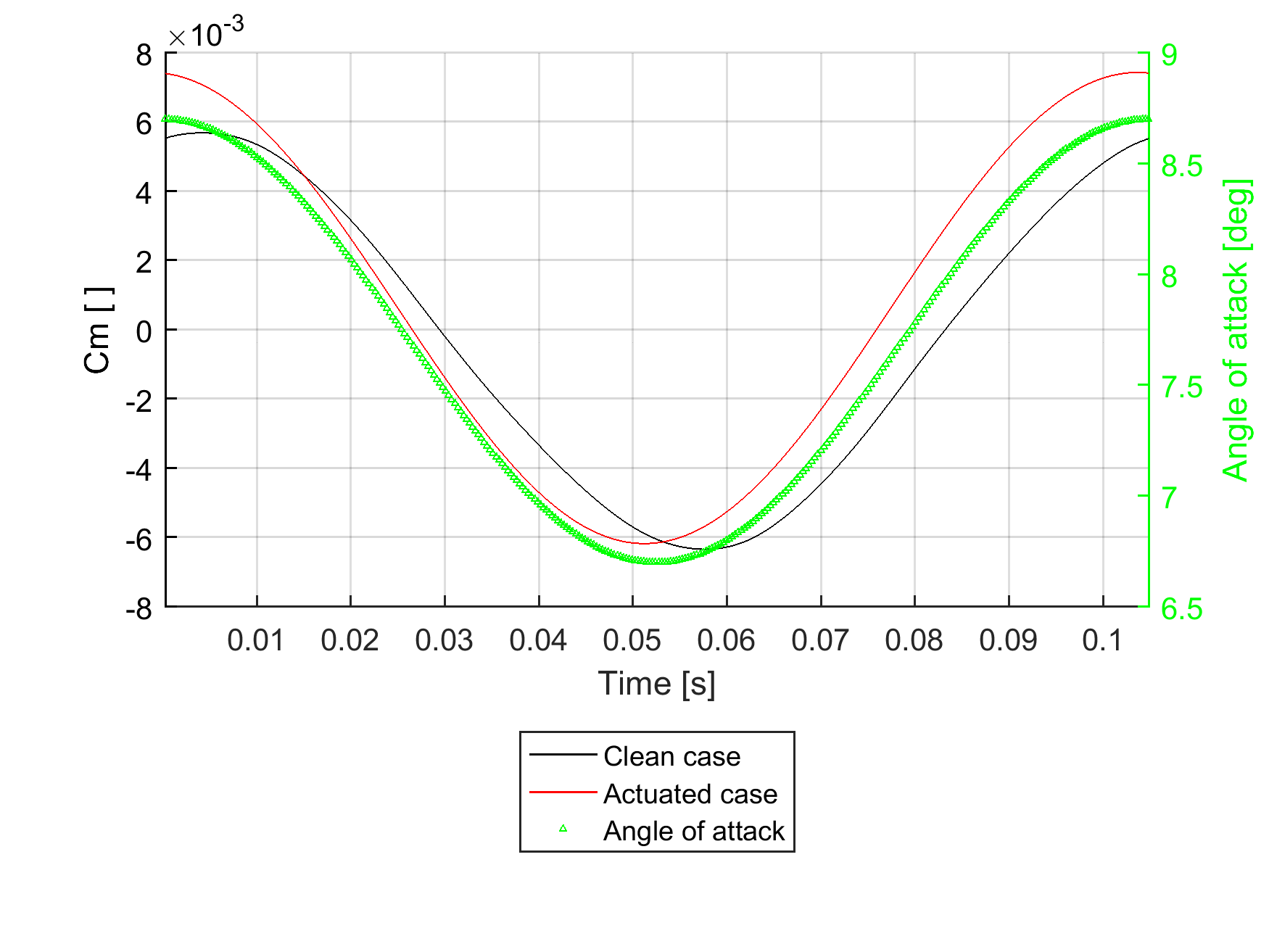

Supplement: Multimedia component 1 [file mmc1.zip › Allegati/w60_a1/Force_w60_a1_90/Moment Coefficient comparison.png]

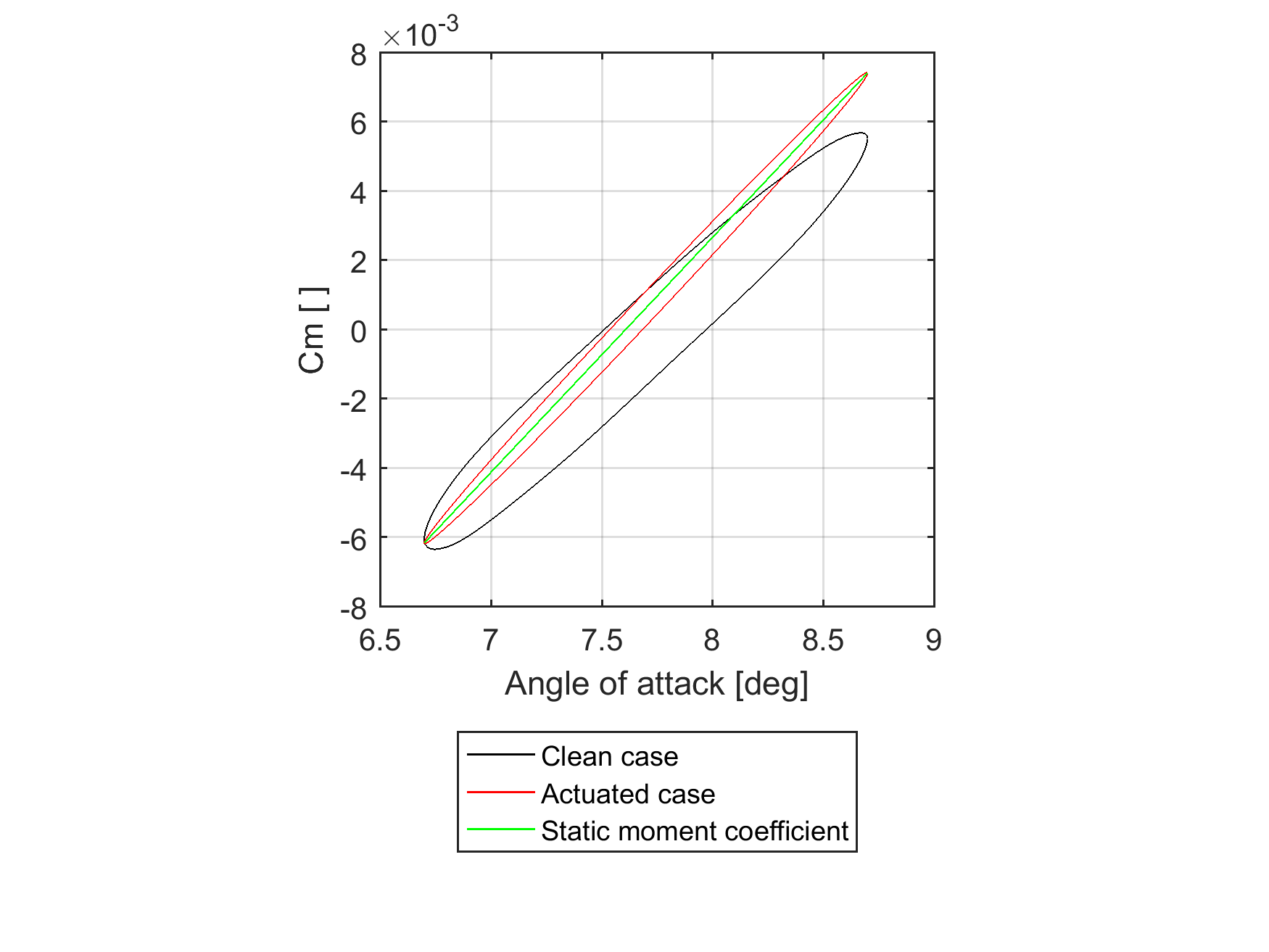

Supplement: Multimedia component 1 [file mmc1.zip › Allegati/w60_a1/Force_w60_a1_90/Moment Coefficient Hysteresis curve.png]
